# Classification of the treble clef zinc finger: noteworthy lessons for structure and function evolution

**DOI:** 10.1038/srep32070

**Published:** 2016-08-26

**Authors:** Gurmeet Kaur, Srikrishna Subramanian

**Affiliations:** 1 CSIR-Institute of Microbial Technology (IMTECH), Sector 39-A, Chandigarh, 160036, India

## Abstract

Treble clef (TC) zinc fingers constitute a large fold-group of structural zinc-binding protein domains that mediate numerous cellular functions. We have analysed the sequence, structure, and function relationships among all TCs in the Protein Data Bank. This led to the identification of novel TCs, such as lsr2, YggX and TFIIIC τ 60 kDa subunit, and prediction of a nuclease-like function for the DUF1364 family. The structural malleability of TCs is evident from the many examples with variations to the core structural elements of the fold. We observe domains wherein the structural core of the TC fold is circularly permuted, and also some examples where the overall fold resembles both the TC motif and another unrelated fold. All extant TC families do not share a monophyletic origin, as several TC proteins are known to have been present in the last universal common ancestor and the last eukaryotic common ancestor. We identify several TCs where the zinc-chelating site and residues are not merely responsible for structure stabilization but also perform other functions, such as being redox active in C1B domain of protein kinase C, a nucleophilic acceptor in Ada and catalytic in organomercurial lyase, MerB.

Zinc fingers (ZFs) are conventionally defined as short protein domains whose tertiary structure (or fold) is primarily stabilized by a structural zinc ion that serves as a surrogate for a strong hydrophobic core^1^,^2^,^3^,^4^. ZFs either as independent entities or as a part of larger proteins are known to play a role in almost all major cellular processes^4^,^5^,^6^. ZFs predominantly function as interaction modules for binding nucleic acids, proteins and other small molecules, such as lipids^3^,^4^,^7^,^8^,^9^. Although rare, examples of enzymatic ZFs have also been documented^3^,^4^. As with functions, ZFs also display diversity in the three-dimensional (3D) structures that they adopt^4^. The zinc chelating residues (mostly Cys and His) may be contributed by different secondary structure elements (SSEs) that may arrange and connect differently, resulting in distinct folds. Based on their 3D structure, ZFs were previously classified into eight-fold groups, of which the C2H2-like, treble clef (TC) and zinc ribbon are the most common^4^.

The TC fold group contains protein domains that perform highly varied functions from acting as molecular scaffolds to being transcription regulators and nucleases, facilitating ubiquitination, trafficking, resistance and sensing of heavy metals, and interacting with membrane lipids, etc.^3^,^10^,^11^,^12^. TCs of the nuclear receptor family of proteins have also been used for *de novo* protein evolution studies^13^,^14^. The core TC fold consists of a zinc knuckle, a loop, a β-hairpin and a α-helix from the N- to the C-terminal^3^,^4^ (Fig. 1A). The core β-hairpin is also referred to as the primary β-hairpin. The zinc knuckle, with a consensus ‘CPXCG’ sequence motif, is commonly seen to occur as a short tight turn of a β-hairpin^3^. The zinc knuckle and the N-terminal region of the α-helix provide two residues each to tetrahedrally chelate a zinc ion.

In the previous classification of ZFs that was released more than a decade ago, the 160 identified TC ZFs from the structurally characterised proteins at the Protein Data Bank (PDB) were grouped into 10 families of evolutionary-related domains^4^. The structural classification of TC ZFs helped to enumerate and understand the various sequence variations and structural modifications that the core of this fold could tolerate and the functions it could perform, and also demonstrated the ability of this fold to bind metal ion(s) at different locations^3^,^4^. Analysis has revealed that TC is one of the ancient protein folds with some members, like the ribosomal protein S14 and L24, HNH/EndoVII-like nucleases, and, at least, one binuclear TC, likely to be present at the time of last universal common ancestor (LUCA)^3^,^10^. Further, evidence for the convergent evolution of short domains such as TCs have also been substantiated^15^.

In anticipation of deriving novel evolutionary inferences, we initialized a survey of sequence-, structure- and function-relationships among all the structurally characterised TCs in the PDB. This led to the identification of many new TCs that had not been identified and annotated by the commonly referred structure- and sequence-based classification repositories, such as Structural Classification of Proteins (SCOP)^16^, Class Architecture Topology Homology (CATH)^17^, and Pfam^18^. We observe an approximately ten-fold increase in the total number of structurally characterised TC ZFs, and many of these do not belong to the previously defined families, prompting us to update the previous structural classification of TC ZFs^3^,^4^. Our analysis has also aided in the rectification of evolutionary grouping of some TC domains that were previously classified into different families. We observe some novel fold topologies and fold overlaps, and many more circularly permuted TCs as compared to what were known previously. Most importantly, this exercise has helped shed light on the alternate functions that could be performed by the zinc-binding site besides structure stabilization.

## Results and Discussion

Using the approaches described in the methods, 608 TC ZFs from 1278 PDB structures of 503 sequentially non-identical proteins were identified. These domains have been classified into 40 families (Supplementary File 1). Considering the classification criteria used previously^3^,^4^, we have grouped many protein domains with the pre-existing families based on inferred evolutionary relationships and others have been placed in novel families that appear to be evolutionarily unrelated to the previous ones. While proteins co-classified within families are likely to share homologous relationships, proteins from different families may or may not be evolutionarily related. TCs being short protein domains with few SSEs, it is likely that some of these families might have emerged independently and share analogous relationships^3^,^4^,^10^,^15^. The most-populated and experimentally well characterised families remain the same, *viz.*, Really Interesting New Gene (RING)-like, His-Me finger, phosphatidylinositol-3-phosphate binding domain-like, and nuclear receptor-like; although the RING-like family in the current classification includes related ZFs of the protein kinase cysteine-rich domain family (for reasons discussed below).

Most newly introduced families are sparsely populated with evolutionarily related zinc-binding domains (ZBDs) from one to few proteins, though, one of them, *viz.* Trafficking, Resistance and Sensing of Heavy metals (TRASH), groups ZFs from many different proteins (Supplementary File 2). The updated classification tabulated in Supplementary File 1 enlists all the member proteins, corresponding PDB identifier(s) (PDBid) and UniProt identifier (UniProtId), Pfam and SCOP identifiers (SCOPid), domain ranges, and PDBid(s) of the protein(s) in complex with other moieties (if any) for each family. The detailed description of each family, including the reasons for specifically grouping some proteins, sequence alignments, structural features and functions performed by members of each family, is provided in Supplementary File 2.

Some important features of the present classification and inferences derived about the TC fold group, in general, are elaborated below.

### Variations to the structural core, fold peculiarities and circular permutations

A characteristic arrangement of SSEs that resembles the ‘treble clef’ musical note, constitutes the core tertiary structure of a mononuclear TC domain^3^,^4^. Variations to this conserved core, including both the loss and presence of additional SSEs, are commonly observed^3^ (Supplementary Figs S1–S3). A tandem non-overlapping pair of TCs is seen in LIM domains where the α-helix of the first TC is often reduced to a short turn. The nuclear receptor DNA-binding domain has a characteristic arrangement of tandem TCs that have plausibly evolved by duplication^3^. The core of the TC fold is extended in binuclear TCs that chelate two zinc ions, of which the first is located at the canonical site of mononuclear TCs and the second is bound by zinc-chelating residues contributed from the turn of the primary β-hairpin and the secondary structure extension to the structural core. In such binuclear TCs, the metal chelating half-sites for the two zinc ions are interleaved or cross-braced, i.e. occur alternatively along the sequence, and the structural core of the second metal-binding site overlaps with the core of the first metal-binding site. This fold topology is characteristic of the RING-like domains, HIT domains, myeloid, Nervy, and DEAF-1 (MYND) domains, Plant Homeo Domain (PHD), AN1, and Fab, YOTB, Vac 1 and EEA1 (FYVE) domains (Supplementary Figs S1–S3). Besides these TCs, a unique binuclear zinc-binding topology is observed in lysine-specific histone demethylase KDM1b (PDBid 4FWE_A)^19^. In KDM1b, a part of the second zinc ion-binding site is contributed by a structurally non-superimposable extended region at the N-terminal of the TC and not from the C-terminal as seen in other cross-braced TCs (Supplementary Fig. S1).

**Figure 1:**
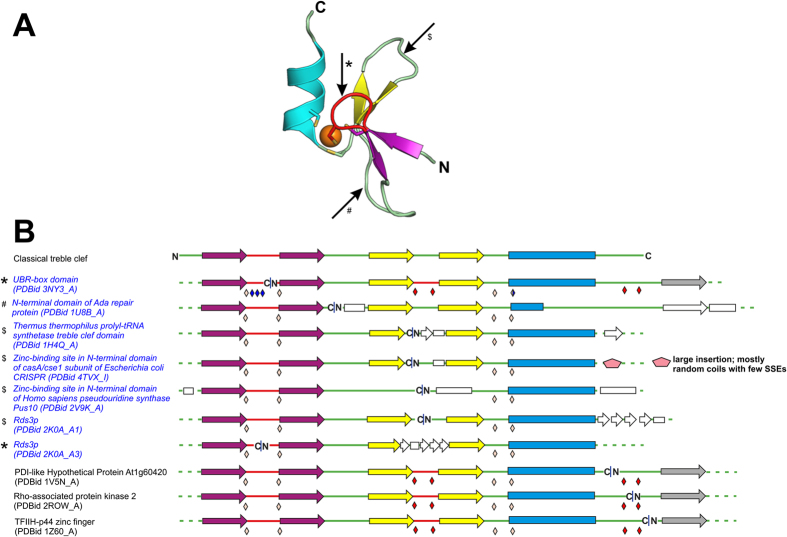
The commonly observed structural core of a treble clef zinc finger fold and its circular permutations. (**A**) Ribbon diagram of the tertiary structure of a TC ZF (PDBid 2DAS_A). (**B**) Linear arrangement of the core SSEs of a classical TC and its circular permutations. In (**A**), the zinc ion and the side-chains of the zinc-chelating residues are shown as sphere and sticks, respectively. In (**B**), the β-strands are shown as arrows and the α-helix as a rectangle, and the location of the zinc-chelating residues is indicated using beige diamonds for the zinc of TC, using red diamonds for the second zinc ion in binuclear TCs and any additional zinc-chelating residues are indicated by blue diamonds. The shared zinc-chelating residue diamond in the UBR-box is double coloured. Labels of the domains with circular permutations in the core of TC fold are in blue. Regions of circular permutations are indicated by a blue pipe ‘**|**’. These observed regions with circular permutation are marked in (**A**) by an arrow and the accompanying symbols (*, $, #) point to proteins in (**B**). Colouring scheme: zinc knuckle: red, primary β-hairpin: yellow, α-helix: cyan, zinc knuckle-containing β-hairpin: purple, zinc ion: orange, SSE insertions and extension: white, conserved additional β-strand in RING-like domains: gray. N- and C-termini of the domain are labeled ‘N’ and ‘C’, respectively.

Unlike the only previously documented example of a circularly permuted TC in the C-terminal domain of *Thermus thermophilus* prolyl-tRNA synthetase (prolyl-RS; PDBid 1H4Q_A)^4^, we now observe many ZBDs whose folds resemble a canonical TC but only after considering a circular permutation. These include the ones seen in pre-mRNA splicing factor Rds3p (PDBid 2K0A_A), CasA/Cse1 subunit of *Escherichia coli* CRISPR (PDBid 4TVX_I), *Homo sapiens* pseudouridine synthase Pus10 (PDBid 2V9K_A), DNA-lesion repair protein Ada (PDBid 1U8B_A) and the UBR-box (PDBid 3NIH_A) (Fig. 1B, Supplementary Fig. S1). The circular permutations to the TC fold are observed in the zinc knuckle, the loop connecting the zinc knuckle with the primary β-hairpin and the loop connecting the β-strands of the primary β-hairpin. Rds3p has a unique knotted fold topology with three GATA-like TCs, one at each vertex of the roughly triangular structure, of which two are circularly permuted as compared to the third one that has a regular TC fold^20^. The UBR-box domain, which has a split zinc knuckle as a consequence of circular permutation, is seen to be additionally stabilized by chelating a third zinc ion via extensions to the conserved RING-like TC core^21^. It is important to mention here that though some of these circular permutations, such as those seen in Rds3p and UBR-box, are likely to represent an evolutionary divergence from bonafide TCs, others might have converged to a similar 3D structure and have been consequently classified as independent families in our present classification. Thus, the term circular permutation used herein is not always indicative of an associated evolutionary event but merely describes the best structural relationship between the domains under comparison^4^. Besides these permuted variants of the TC fold, many domains with a RING-like TC fold exhibit circular permutations in regions outside of the core of the mononuclear TCs. We record a novel topological variant of the binuclear RING-like fold in PDI-like hypothetical protein At1g60420 (PDBid 1V5N_A) that is distinct from the C1 domain (PDBid 2ROW_A) and TFIIH-p44 ZF (PDBid 1Z60_A) that were documented earlier^3^,^4^ (Fig. 1, Supplementary Fig. S1).

### Evolutionary fold change and loss of zinc chelation

Novel structural topologies emerge in proteins via phenomena such as circular permutations, β-strand- and 3D domain-swaps, β-strand and β-hairpin invasions, duplication and fusion^22^,^23^,^24^. ZFs are one of the ancient protein folds, with TCs suggested to have evolved before the emergence of the LUCA and last eukaryotic common ancestor (LECA)^3^,^10^,^25^. Our survey and analysis of members of the TC fold revealed unanticipated similarities to some protein domains that were hitherto considered as novel folds. We have shown that the unusual fold (SCOPid 160386) seen in the nitrous oxide reductase pathway protein NosL and the catalytic domain of organomercurial lyase, MerB, emerged by duplication and fusion of TRASH-like TCs^26^. Duplication of the ZF accompanied by loss of zinc-binding and gain of additional SSEs and stabilizing forces, such as the formation of hydrogen bonds within extended β-sheets, plausibly led to redefining the core of the resulting scaffold. Likewise, the UBR-box domain (PDBid 3NIJ_A) was previously proposed to have a novel three-zinc stabilized heart-shaped structure with two α-helices, two antiparallel β-strands and long ordered loops with no relation to any other protein folds^27^. Our analysis, on the contrary, identified this fold to have emerged in eukaryotes from a RING-like binuclear TC ancestor by a circular permutation event that likely aided the formation of a novel peptide binding interface^21^.

A distinct zinc-binding fold is seen in the CW domain (PDBid 4GUS_A), the zinc-binding region of E7 oncoprotein (PDBid 2EWL_A) and the ZF of Ash2L protein (PDBid 3S32_A), where a single zinc ion is chelated by aminoacids located at the turn of an N-terminal β-hairpin and the start of a C-terminal α-helix (Supplementary File 2: Supplementary Fig. vi). This zinc-binding topology is unlike other ZF folds but shares structure similarity with the second zinc-binding site of PHD ZFs^28^,^29^,^30^,^31^,^32^ that is indicative of their plausible evolutionary relatedness^28^. Further, though the zinc knuckle is disordered in the structurally characterised ZF of human Ash2L that also lacks zinc-chelating residues corresponding to the first zinc ion of the PHDs, we are able to retrieve several Ash2L homologs (such as from *Drosophila melanogaster,* UniProtId Q94545 and *Schizosaccharomyces pombe,* UniProtId O60070) during our sequence analysis that possess metal-chelating residues corresponding to both the zinc-chelating sites and are likely to adopt a cross-braced binuclear TC structure. Thus, Ash2L ZF serves as an evident example in support of evolutionary fold transition and demonstrates a plausible pathway for the emergence of a unique fold in consequence to change in zinc-binding properties.

Such structural modifications and sequences changes, including loss of zinc-chelating residues, has hindered the detection of similarities among several other members of this fold group, for example, MH1 domain of Smad1^33^. Many members of the His-Me finger family^3^,^33^, YlxR-like hypothetical cytosolic protein (PDBid 1G2R_A) family^4^ and the U-box domain family^34^ are also seen to have variable loss of the zinc-chelating residues although they possess the conserved TC fold. Our analysis has helped discover many novel TCs whose structurally characterised homologs lack a zinc ion that hampered their identification and annotation. The RING-like domain of TFIIIC τ 60 KDa subunit (PDBid 2J04_A^35^; see description for the RING-like family in Supplementary File 2), α-COP (PDBid 3MKR_B; detailed description in^36^), the duplicated and fused TCs in the NosL/MerB-like fold (PDBids 2HPU_A, 3F0P_A; detailed description in^26^), conserved Gram-negative bacterial protein YggX (PDBid 1YHD_A^37^; see description in (Supplementary File 2), and conserved actinobacterial protein lsr2 (PDBid 4E1P_A^38^; see description in (Supplementary File 2) are the novel TCs discovered during our analysis.

### Zinc-chelating residues of the structural zinc site are reactive in some proteins and catalytically active in a particular instance

**Figure 2:**
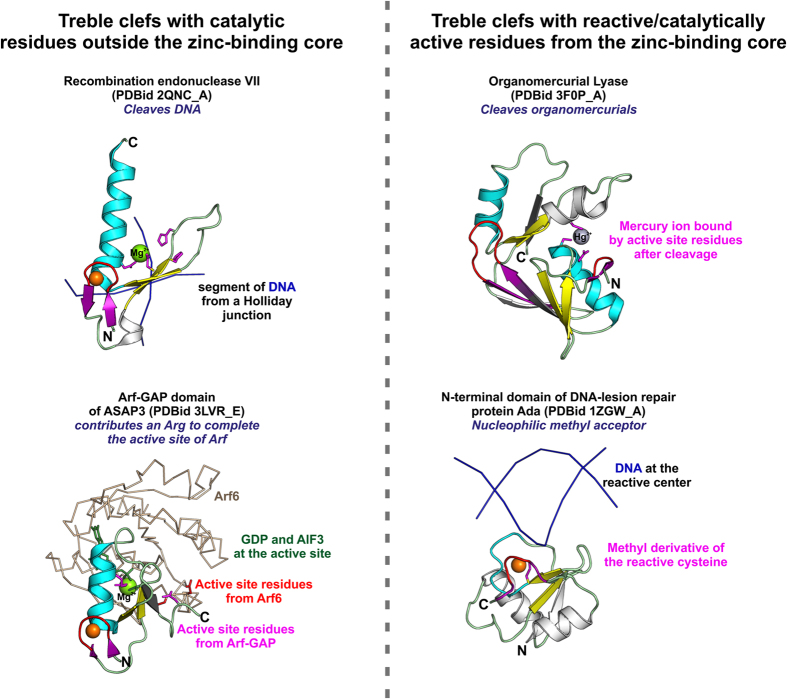
Comparison of all catalytic treble clefs. The protein structures on the left are of catalytic TCs whose active site resides outside the zinc-binding core and those on right are of TCs whose reactive/active-site residues are from the zinc-binding core. The basic colouring scheme follows Fig. 1; side-chains of active-site/reactive residues are coloured magenta. TCs are shown as ribbons and the DNA/protein partners are shown as backbone trace.

The ability of TC ZFs to mediate catalysis was known for proteins of the His-Me family^3^ and some zinc binding *de novo* synthetic peptides^15^,^39^,^40^ (Fig. 2). We include the TC ZF of Arf-GAP in the list of catalytic TCs as a conserved Arg, the “arginine finger”, from the α-helix of Arf-GAP TC is known to complete the active site of the interacting Arf (PDBid 3LVR_E)^41^,^42^. In all these proteins the enzymatic function is performed by residues in the regions (SSEs) outside the zinc-binding site, similar to the non-catalytic functional sites of TCs. Interestingly, we have observed several TCs during our analysis where the structural zinc-binding core itself is also reactive.

As discussed above, the NosL/MerB-like fold emerged from a TC domain. The structural core of the NosL/MerB-like fold is no longer zinc-stabilized, and the duplication and fusion have perhaps relaxed the evolutionary pressure on the metal-binding site to serve a structure-stabilizing role^26^. This, accompanied by the structural changes such as the flipping of the knuckle-β-hairpin, plausibly allowed the exaptation of the metal-chelating site to bind larger organometal moieties by MerB. In MerB, the ancestral structure-stabilizing metal-binding site constitutes a part of the active site in the extant protein and the structural metal chelating residues of the ancestral fold are catalytically active in mediating cleavage of organometals (Fig. 2).

Similarly, one of the four metal-chelating cysteines of the N-terminal ZBD of Ada is reactive and acts as a nucleophilic acceptor for the suicidal transfer of methyl groups from damaged DNA onto itself^43^,^44^. This zinc is also essential for proper folding of the domain whose tertiary structure consists of a four-stranded β-sheet surrounded by two α-helices and a single zinc ion^43^,^45^,^46^. However, based on the then prevalent notion of a strict structural role for the zinc-binding site in ZFs, the ZBD of Ada was not considered to be a conventional ZF and consequently excluded from the previous classification^4^. During our literature survey, we have become aware of reports of several other ZFs, such as the C1B domain of protein kinase Cα (member of the RING-like family) and the C-terminal ZBD of Hsp33 (with a fold related to the zinc ribbons), where the residues involved in zinc binding at the core of the fold are shown to be reactive (redox active in these proteins)^47^,^48^. Thus, given the possibility of residues involved in structural zinc chelation to exhibit reactivity, we have included all such domains in the present classification.

The fold of the ZF of Ada resembles a circularly permuted TC but lacks a well-defined α-helix (details in (Supplementary File 2: (Supplementary Fig. v). In all the examples of TC ZFs with reactive or catalytic metal-chelating residues enlisted here, we observe the presence of an additional β-strand after the α-helix that forms an antiparallel β-sheet with the primary β-hairpin. In the case of C1 domains, this extension is similar to that seen in other members of the RING-like family^3^,^21^. This β-strand has been suggested to be important for providing additional stability to the domain in the absence of zinc-binding^34^. Thus, it is plausible that the compensatory-stability provided by the appendages to the core ZF fold and/or by domain duplication and fusion, as seen in the NosL/MerB-like fold, may aid the emergence of additional functions at the structure stabilizing zinc binding site.

### Overlap of the TC fold with other protein folds in some proteins

Protein domains are usually defined by referring to a distinct fold with discrete structural boundaries and may combine in different ways to give rise to new proteins and functions. Concatenated and nested domains are commonly observed but overlapping (interlaced) protein domains are rare^49^. Interlaced domains have an alternating arrangement of SSEs of two folds along a single polypeptide. Thus, the overall structure of such domains may be defined by referring to only one of the folds and considering additional insertions and extensions to its core, or one may define two domains with distinct folds but with overlapping core SSEs. Overlapping domains, thus, may be thought as connecting points among folds occupying different regions in the global protein structural space and emphasize the continuity of protein fold space.

**Figure 3:**
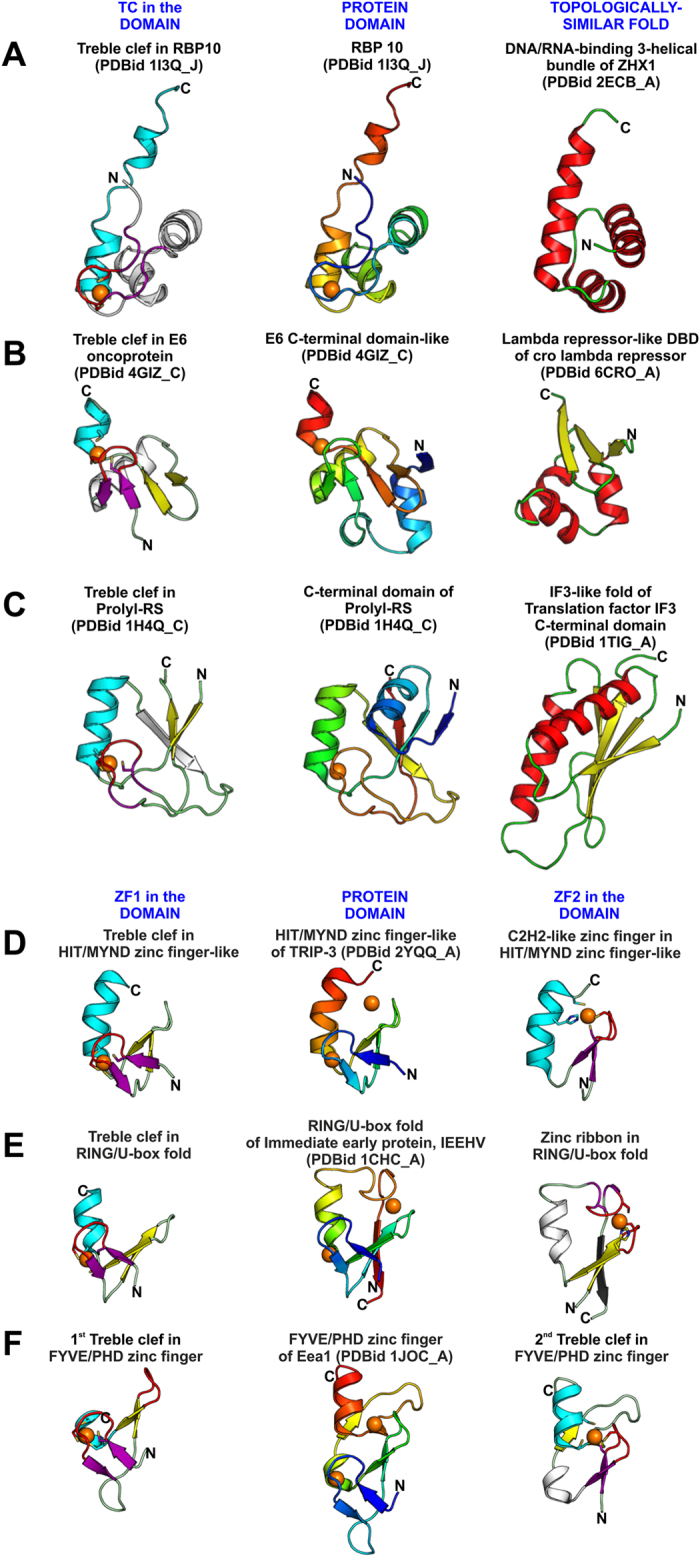
Treble clef zinc fingers that overlap with other protein folds. (**A**–**C**) The TC ZFs shown on the left are contained within the fold of the protein domains shown in the middle. However, this fold is also similar to other bonafide protein folds that are shown on the right. **(D**–**F**) The ZFs on the left and right are contained within the fold of the protein domains shown in the middle. Colouring scheme: for TCs follows that used in Fig. 1; other ZFs are coloured as per their standard colouring in^4^; structures in the middle: coloured in a gradient of blue to red from the N to C termini; structures on the left are coloured with α-helices-red, β-strands-yellow, loops-green.

During our analysis, we have identified several interlaced domains that bind a structural zinc ion. The zinc-binding site, defined by the placement of the metal-chelating residues and the SSEs which contribute them, resembles a bonafide ZF, while the tertiary structure of the complete domain discounting the zinc ion could be related to other protein folds. Domains in which the zinc-binding site resembles a TC motif include the C-terminal domain of *T. thermophilus* prolyl-RS (PDBid 1H4Q_A), *Methanothermobacter thermautotrophicus* RNA polymerase subunit RPB10 (PDBid 1EF4_A) and each of the duplicated domain in human papillomavirus E6 oncoprotein (PDBid 4GIZ_C) (Fig. 3A–C). The C-terminal domain of prolyl-RS resembles an IF3-like fold (SCOPid 64586), that of RNA polymerase subunit RPB10 resembles a DNA/RNA-binding 3-helical bundle (SCOPid 46924), and the ZF of E6 oncoprotein shares topological similarities with the lambda cro protein fold (SCOPid 161228). However, in all these cases the sequence similarity of the domain in question with bonafide members of the TC fold group or the other protein fold is only marginal and is suggestive of convergence. Hence, we refer to each of these as separate families that may or may not be evolutionarily related to other TC families, or, for that matter, families of the structurally related protein fold. Likewise, the binuclear cross-braced TCs appear to consist of two overlapping ZFs. For example, the second zinc-binding site in the HIT/MYND ZF-like, the RING/U-box-like, and the FYVE/PHD ZFs resembles a C2H2-like ZF, a circularly-permuted zinc ribbon, and a TC-like ZF, respectively (Fig. 3D–F). Thus, the composite domains are seen to have interlaced zinc-binding sites and overlapping SSEs that form the core of two different ZF folds.

### Predicted function for DUF1364

Prophage-derived ybcO (PDBid 3G27_A) is a protein of unknown function (DUF1364; PF07102) with a TC fold. Our sequence-based analysis can relate ybcO to bonafide members of the His-Me finger family. For example, FFAS search with ybcO (PDBid 3G27_A) retrieves matches to recombination enhancement function protein (PDBid 3PLW_A, Score = −24.7) and putative HNHc nuclease (PF16784, Score = −18.7). YbcO has a histidine on the primary β-hairpin of the TC that superimposes on the catalytic histidine of His-Me nucleases ((Supplementary File 2: (Supplementary Fig. viii). This histidine is well conserved in members of the DUF1364 family. Thus, we predict that ybcO is likely to function as a nuclease.

### Regrouping of previously misclassified families

#### B-box domain has been moved from the zinc ribbon fold group to the RING-like TCs family

B-box was placed in the zinc ribbon fold group in the previous ZF classification based on the only available structure of nuclear factor XNF7 (PDBid 1FRE_A)^4^. This structure consisted of two β-strands, two α-helical turns, three loop regions, and a single zinc ion, although other conserved potential metal-chelating residues were present in the sequence that could likely chelate a second zinc ion. This structure failed most of the PROCHECK quality checks and was classified as a zinc ribbon with a cautionary note^4^. Based on the currently available sequences and structures of the B-box domains ((Supplementary Files 1 and 2), it has been placed in the RING-like family of the TC fold group. B-box in all structures but that of XNF7 possesses a RING-like cross-braced binuclear TC fold. These domains lack the typical ‘squiggle’ and instead have an extended region after the core α-helix that contributes residues to bind the second metal ion. Dali structure similarity search with B-box (PDBid 3Q1D_A) could retrieve RING domain of Baculoviral IAP repeat-containing protein 7 (PDBid 4AUQ_B; Z-score = 3.6, RMSD = 2.0 Å, nali = 39) and FFAS sequence similarity search with B-box (PDBid 2DJA_A) could retrieve UBR-box of E3 ubiquitin-protein ligase UBR1 (PDBid 3NIT_A; Score = −11.4) and RING domain of zinc finger protein 183-like 1 (PDBid 2CSY_A; Score = −10.1).

#### The ZFs from the protein kinase cysteine-rich domain family and the RING family are grouped together

The binuclear TCs of the protein kinase C (PKC) C1 domain and TFIIH-p44 subunit cysteine-rich domain (CRD) were previously placed in a separate family, but their structures were identified to be related to the RING-like fold by distinct circular permutations and plausible evolutionary relatedness was also suggested^4^. Analysis of the available structures reveals that CRD of At1g60420 (PDBid 1V5N_A) could also be related to the RING-like core by yet another distinct circular permutation at the second zinc-binding half-site of the second zinc ion (Fig. 1, (Supplementary File 2: (Supplementary Fig. vi). Sequence-based searches reveal the similarity of At1g60420 CRD (PDBid 1V5N_A) to the C1 domains (HHpred finds PDBid 1RFH_A; E-value = 2.8E-06) and ZZ domains (HHpred finds PDBid 1TOT_A; E-value = 4.6E-05). ZZ domains are cross-braced binuclear TCs that share sequence similarity with other RING-like domains, such as UBR-box (HHpred finds PDBid 3NY3_A; E-value = 0.0081) and B-box (HHpred finds PDBid 2MVW_A; E-value = 0.054). Thus, based on the current sequences and structures, a common evolutionary origin for the RING-like domains, PKC C1, CRD of TFIIH-p44 and At1g60420, and ZZ domains seems plausible, as also proposed previously^10^. Therefore, all these have been grouped under the RING-like family in the present classification.

#### YacG family merged with TRASH family, and L24 moved from the nuclear receptor family to the TRASH family

YacG (PDBid 1LV3_A) was placed as an autonomous family in the previous ZF classification because of lack of sequence similarity to any other proteins^4^. Supported by significant sequence similarity, YacG appears to be related to members of the TRASH family. HHpred initiated with YacG (PDBid 1LV3_A) retrieves FCS domain of Polyhomeotic-like protein 1 (PDBid 2L8E_A; E-value = 0.0043) and MYND domain of DEAF1 (PDBid 2JW6_A; E-value = 0.051).

Likewise, ribosomal protein L24 has been grouped with the TRASH family and removed from the nuclear receptor family based on sequence similarity. For example, FFAS with L24 (PDBid 3U5E_W) finds matches to YHS domain family (PF04945; Score = −11.400) and HHpred finds TC_1 of NosL (PDBid 2HPU_A; E-value = 5.1E-06).

### Summary and Conclusion

We have analysed the TC domains in all the structurally characterised proteins in the PDB. Our classification of the TC fold group has structural representatives from 97 Pfam families (v27.0) and 28 SCOP folds (v1.75) (all Pfam families and SCOP folds that annotate the ZF, even as a remark in larger families/folds, are enumerated). Additionally, we have discovered many ZFs that are not currently classified in these databases. We also find that Pfam misclassifies some TCs, such as the zf-FPG_IleRS (PF06827) and zf-dskA_traR (PF01258) that appear under the Zn_Beta_Ribbon clan (CL0167), and zf-AD (PF07776) that appears under the C2H2-zf clan (CL0361). This is probably because of the high conservation of metal-chelating aminoacids in the HMM profile of these families that causes biased sequence similarity with other families possessing similar patterns of conservation. Thus, based on manual analysis of their structure, the ZFs from these families have been unambiguously grouped under the TC fold. Our analysis led to the discovery of many novel TC domains such as TFIIIC, YggX, and lsr2.

TC is one of the very versatile protein folds that performs varied functions and can embrace various structural modifications. We have identified and documented the circular permutations of the TC fold in the available structures. We also observe that the TC domain from the UBR-box of N-recognins (PDBid 3NIH_A), RING domain of RAG1 (PDBid 1RMD_A), and FYVE-like ZF of E3 ligase CHFR (PDBid 2XOC_A), possess an additional zinc ion that forms a metal ion cluster with the zinc ion of the TC by sharing metal-chelating residue(s). In proteins, including RPB10, prolyl-RS, and E6 oncoprotein, the core structural elements of the TC are seen to overlap with the core SSEs of other bonafide proteins folds. The TC module may also serve as the building block for the evolutionary emergence of several new folds such as those observed in NosL/MerB-like fold, UBR-box, and ZF of Ash2L, CW and E7 oncoprotein. Functional diversity of TCs is not only evident from the extensive experimental literature but is also emphasized by its tendency to co-occur with a large variety of other domains ((Supplementary File 3). Different surface areas of the TC fold have been utilized in varied proteins to mediate dimerization and interactions with other biomolecules such as DNA, RNA, proteins and lipids ((Supplementary File 2: (Supplementary Figs xv–xvii). Some TCs possess a reactive zinc site where the zinc-chelating residues are redox or enzymatically active. In one example, *viz*. MerB, the evolutionary repurposing of a non-catalytic structural scaffold into a novel enzyme is seen^26^.

## Methods

In brief, an exhaustive search for all the TC ZFs was done (details in (Supplementary File 4). Each of the TC ZF in the collected dataset was studied in detail, and structure and sequence similarity analysis were done for all members using automated methods and manual examination. BLAST^50^, PSI-BLAST^51^, FFAS^52^, tools from the HMMER3 package^53^ and HHpred^54^ were used for automated sequence-similarity based searches. Dali^55^, TM-align^56^, Fr-TM-align^57^ and TopSearch^58^ were used for assessment of structure similarity. Sequence-based clustering was done using cd-hit^59^, and multiple sequence alignments were generated using PROMALS3D^60^ and followed by manual adjustments. Neighbouring domains were studied by referring to conserved domain database (CDD)^61^.

## Additional Information

**How to cite this article**: Kaur, G. and Subramanian, S. Classification of the treble clef zinc finger: noteworthy lessons for structure and function evolution. *Sci. Rep.* **6**, 32070; doi: 10.1038/srep32070 (2016).

## Supplementary Material

Supplementary Information

Supplementary File 1

Supplementary File 2

Supplementary File 3

Supplementary File 4
