# Supplementary material for: Classification of the treble clef zinc finger: noteworthy lessons for structure and function evolution
Source: Sci Rep. 2016 Aug 26;6:32070. doi: 10.1038/srep32070 (PMC4999995; doi:10.1038/srep32070)
Supplement: Supplementary Information [file srep32070-s1.pdf]

Title:

Classification of the treble clef zinc finger: noteworthy lessons for structure and function evolution

Authors:

Gurmeet Kaur, Srikrishna Subramanian[1]

Contact Information

CSIR-Institute of Microbial Technology (IMTECH), Sector 39-A, Chandigarh, 160036, India. Phone: +911726665483; Fax: +91-1722695215; Email: krishna@imtech.res.in

[1] Corresponding author

Supplementary Figures.

Figure S1. Structural variations of the treble clef fold. The observed structural variations to the core TC fold are shown using the ribbon diagrams for the respective domains. Colouring scheme: as in Fig. 1, mercury: gray. Large insertions in the core and disordered regions in the structures are represented by dotted lines.

Figure S2: Insertions and extensions to the core of the treble clef zinc finger fold. Colouring scheme and representation: as in Fig. 1. The additional α-helix and β-strands of the FYVE are coloured in a lighter tint. 3_{10}-helices are represented as rectangles with serrated edges. Additional loops regions between the knuckles are shown as loop-like protrusions. The SSEs in brackets ('[ ]') are not conserved in all the structurally characterised members.

Figure S3: Topology diagrams of treble clef domains. SSEs of the TC are coloured and represented as in Fig. 1. β-strands that are placed closely are involved in hydrogen bonding and form β-hairpins/β-sheets. In some proteins such as RPB10, hypothetical protein ORF126, prolyl-RS, etc., few SSEs are boxed in different colours because their topological connections are such that they could be independently structurally-related to other protein folds (as mentioned in the figure), but, none-the-less the overall zinc-binding site resembles a TC motif. In MerB (PDBid 3F0P_A1) and ZF of E6 oncoprotein TC2 (PDBid 4GIZ_C), the knuckle-β-hairpin forms a β-sheet by hydrogen bonding with the primary β-hairpin and for these, the α-helix is shown behind the plane of the β-sheet.

FIGURE S1

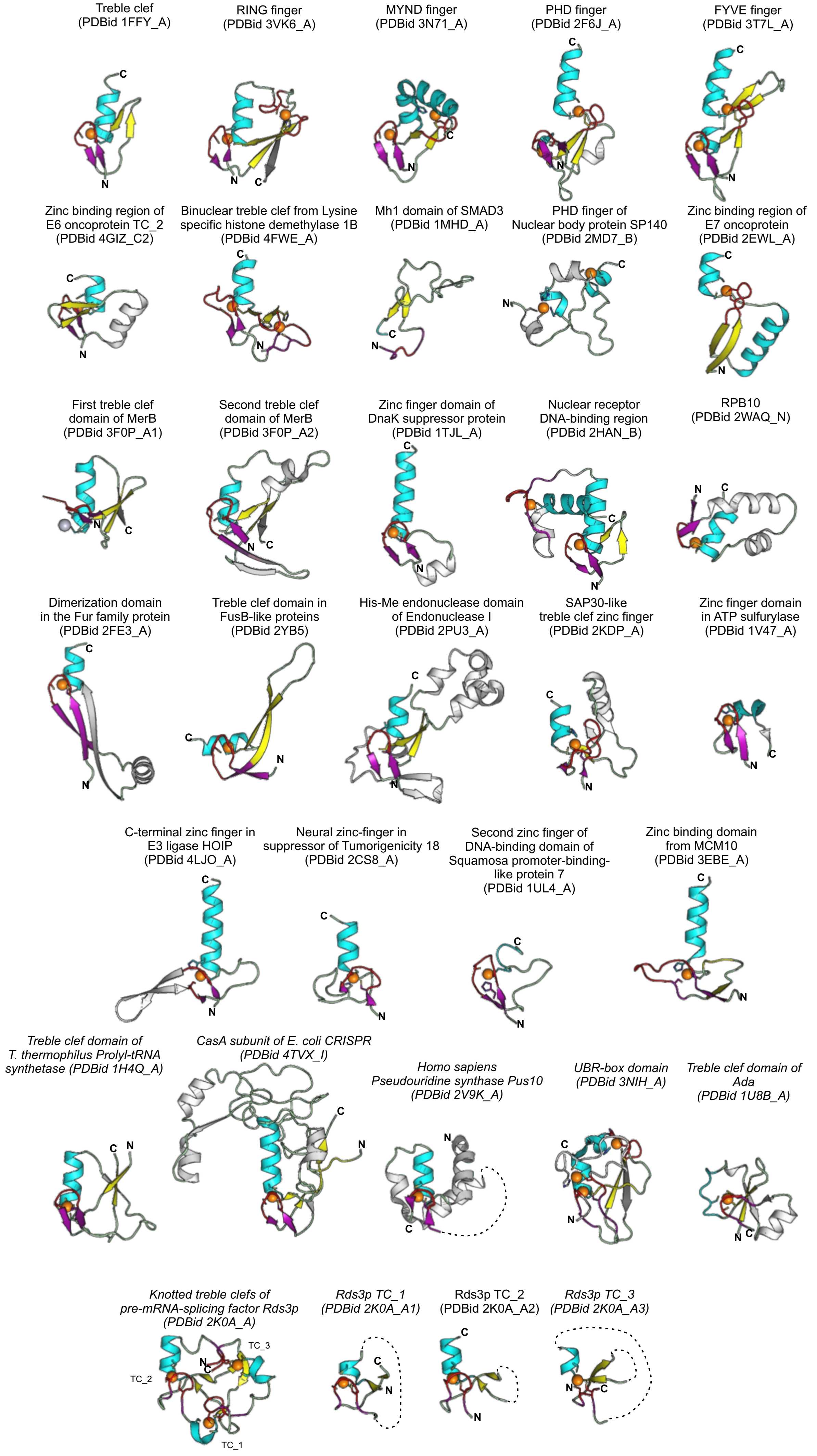

Treble clef
(PDBid 1FFY_A)
RING finger
(PDBid 3VK6_A)
MYND finger
(PDBid 3N71_A)
PHD finger
(PDBid 2F6J_A)
FYVE finger
(PDBid 3T7L_A)
Zinc binding region of
E6 oncoprotein TC_2
(PDBid 4GIZ_C2)
Binuclear treble clef from Lysine
specific histone demethylase 1B
(PDBid 4FWE_A)
Mh1 domain of SMAD3
(PDBid 1MHD_A)
PHD finger of
Nuclear body protein SP140
(PDBid 2MD7_B)
Zinc binding region of
E7 oncoprotein
(PDBid 2EWL_A)
First treble clef
domain of MerB
(PDBid 3F0P_A1)
Second treble clef
domain of MerB
(PDBid 3F0P_A2)
Zinc finger domain of
DnaK suppressor protein
(PDBid 1TJL_A)
Nuclear receptor
DNA-binding region
(PDBid 2HAN_B)
RPB10
(PDBid 2WAQ_N)
Dimerization domain
in the Fur family protein
(PDBid 2FE3_A)
Treble clef domain in
FusB-like proteins
(PDBid 2YB5)
His-Me endonuclease domain
of Endonuclease I
(PDBid 2PU3_A)
SAP30-like
treble clef zinc finger
(PDBid 2KDP_A)
Zinc finger domain
in ATP sulfurylase
(PDBid 1V47_A)
C-terminal zinc finger in
E3 ligase HOIP
(PDBid 4LJO_A)
Neural zinc-finger in
suppressor of Tumorigenicity 18
(PDBid 2CS8_A)
Second zinc finger of
DNA-binding domain of
Squamosa promoter-binding-
like protein 7
(PDBid 1UL4_A)
Zinc binding domain
from MCM10
(PDBid 3EBE_A)
Treble clef domain of
T. thermophilus Prolyl-tRNA
synthetase (PDBid 1H4Q_A)
CasA subunit of E. coli CRISPR
(PDBid 4TVX_I)
Homo sapiens
Pseudouridine synthase Pus10
(PDBid 2V9K_A)
UBR-box domain
(PDBid 3NIH_A)
Treble clef domain of
Ada
(PDBid 1U8B_A)
Knotted treble clefs of
pre-mRNA-splicing factor Rds3p
(PDBid 2K0A_A)
TC_3
TC_2
TC_1
Rds3p TC_1
(PDBid 2K0A_A1)
Rds3p TC_2
(PDBid 2K0A_A2)
Rds3p TC_3
(PDBid 2K0A_A3)

FIGURE S2

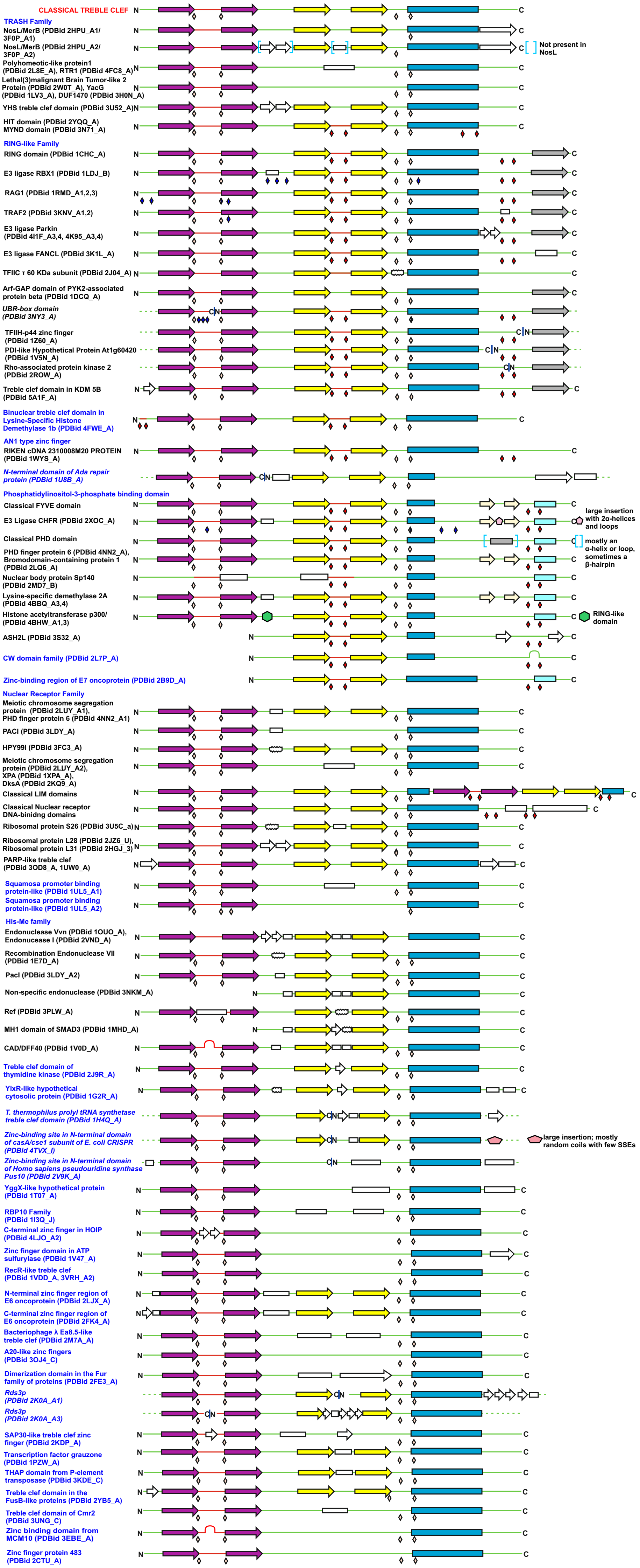
CLASSICAL TREBLE CLEF
TRASH Family
NosL/MerB (PDBid 2HPU_A1/ 3F0P_A1)
NosL/MerB (PDBid 2HPU_A2/ 3F0P_A2)
Not present in NosL
Polyhomeotic-like protein1 (PDBid 2L8E_A), RTR1 (PDBid 4FC8_A)
Lethal(3)malignant Brain Tumor-like 2 Protein (PDBid 2W0T_A), YacG (PDBid 1LV3_A), DUF1470 (PDBid 3H0N_A)
YHS treble clef domain (PDBid 3U52_A)
HIT domain (PDBid 2YQQ_A) MYND domain (PDBid 3N71_A)
RING-like Family
RING domain (PDBid 1CHC_A)
E3 ligase RBX1 (PDBid 1LDJ_B)
RAG1 (PDBid 1RMD_A1,2,3)
TRAF2 (PDBid 3KNV_A1,2)
E3 ligase Parkin (PDBid 4I1F_A3,4, 4K95_A3,4)
E3 ligase FANCL (PDBid 3K1L_A)
TFIIC τ 60 KDa subunit (PDBid 2J04_A)
Arf-GAP domain of PYK2-associated protein beta (PDBid 1DCQ_A)
UBR-box domain (PDBid 3NY3_A)
TFIIH-p44 zinc finger (PDBid 1Z60_A)
PDI-like Hypothetical Protein At1g60420 (PDBid 1V5N_A)
Rho-associated protein kinase 2 (PDBid 2ROW_A)
Treble clef domain in KDM 5B (PDBid 5A1F_A)
Binuclear treble clef domain in Lysine-Specific Histone Demethylase 1b (PDBid 4FWE_A)
AN1 type zinc finger
RIKEN cDNA 2310008M20 PROTEIN (PDBid 1WYS_A)
N-terminal domain of Ada repair protein (PDBid 1U8B_A)
Phosphatidylinositol-3-phosphate binding domain
Classical FYVE domain
E3 Ligase CHFR (PDBid 2XOC_A)
large insertion with 2α-helices and loops
Classical PHD domain
mostly an α-helix or loop, sometimes a β-hairpin
PHD finger protein 6 (PDBid 4NN2_A), Bromodomain-containing protein 1 (PDBid 2LQ6_A)
Nuclear body protein Sp140 (PDBid 2MD7_B)
Lysine-specific demethylase 2A (PDBid 4BBQ_A3,4)
Histone acetyltransferase p300/ (PDBid 4BHW_A1,3)
RING-like domain
ASH2L (PDBid 3S32_A)
CW domain family (PDBid 2L7P_A)
Zinc-binding region of E7 oncoprotein (PDBid 2B9D_A)
Nuclear Receptor Family
Meiotic chromosome segregation protein (PDBid 2LUY_A1), PHD finger protein 6 (PDBid 4NN2_A1)
PACI (PDBid 3LDY_A)
HPY99I (PDBid 3FC3_A)
Meiotic chromosome segregation protein (PDBid 2LJY_A2), XPA (PDBid 1XPA_A), DksA (PDBid 2KQ9_A)
Classical LIM domains
Classical Nuclear receptor DNA-binidng domains
Ribosomal protein S26 (PDBid 3U5C_a)
Ribosomal protein L28 (PDBid 2JZ6_U), Ribosomal protein L31 (PDBid 2HGJ_3)
PARP-like treble clef (PDBid 3OD8_A, 1UW0_A)
Squamosa promoter binding protein-like (PDBid 1UL5_A1)
Squamosa promoter binding protein-like (PDBid 1UL5_A2)
His-Me family
Endonuclease Vvn (PDBid 1OUO_A), Endonucease I (PDBid 2VND_A)
Recombination Endonuclease VII (PDBid 1E7D_A)
PacI (PDBid 3LDY_A2)
Non-specific endonuclease (PDBid 3NKM_A)
Ref (PDBid 3PLW_A)
MH1 domain of SMAD3 (PDBid 1MHD_A)
CAD/DFF40 (PDBid 1V0D_A)
Treble clef domain of thymidine kinase (PDBid 2J9R_A)
YlxR-like hypothetical cytosolic protein (PDBid 1G2R_A)
T. thermophilus prolyl tRNA synthetase treble clef domain (PDBid 1H4Q_A)
Zinc-binding site in N-terminal domain of casA/cse1 subunit of E. coli CRISPR (PDBid 4TVX_I)
large insertion; mostly random coils with few SSEs
Zinc-binding site in N-terminal domain of Homo sapiens pseudouridine synthase Pus10 (PDBid 2V9K_A)
YggX-like hypothetical protein (PDBid 1T07_A)
RBP10 Family (PDBid 1I3Q_J)
C-terminal zinc finger in HOIP (PDBid 4LJO_A2)
Zinc finger domain in ATP sulfurylase (PDBid 1V47_A)
RecR-like treble clef (PDBid 1VDD_A, 3VRH_A2)
N-terminal zinc finger region of E6 oncoprotein (PDBid 2LJX_A)
C-terminal zinc finger region of E6 oncoprotein (PDBid 2FK4_A)
Bacteriophage λ Ea8.5-like treble clef (PDBid 2M7A_A)
A20-like zinc fingers (PDBid 3OJ4_C)
Dimerization domain in the Fur family of proteins (PDBid 2FE3_A)
Rds3p (PDBid 2K0A_A1)
Rds3p (PDBid 2K0A_A3)
SAP30-like treble clef zinc finger (PDBid 2KDP_A)
Transcription factor grauzone (PDBid 1PZW_A)
THAP domain from P-element transposase (PDBid 3KDE_C)
Treble clef domain in the FusB-like proteins (PDBid 2YB5_A)
Treble clef domain of Cmr2 (PDBid 3UNG_C)
Zinc binding domain from MCM10 (PDBid 3EBE_A)
Zinc finger protein 483 (PDBid 2CTU_A)

FIGURE S3

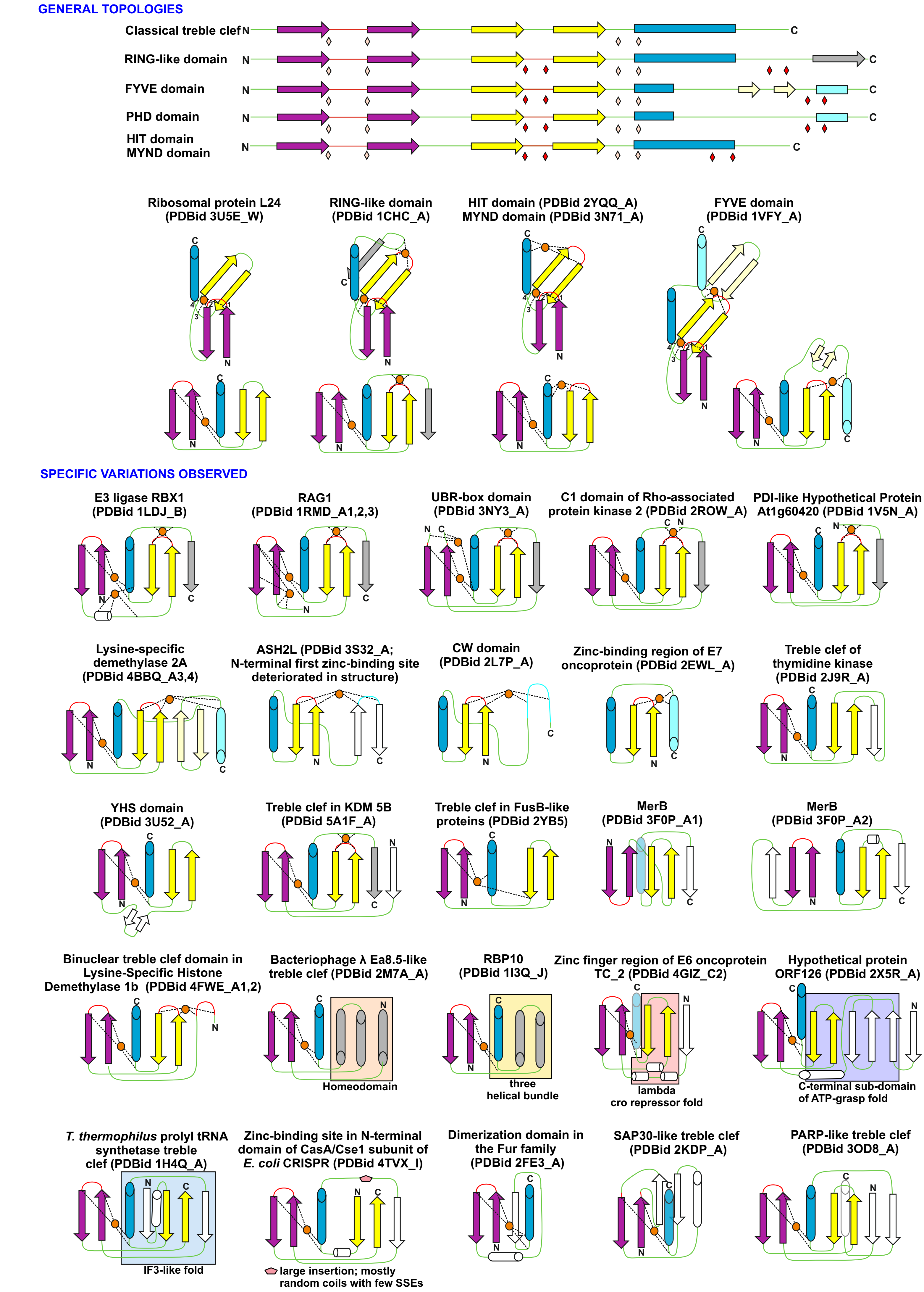
GENERAL TOPOLOGIES
Classical treble clef
RING-like domain
FYVE domain
PHD domain
HIT domain
MYND domain
Ribosomal protein L24 (PDBid 3U5E_W)
RING-like domain (PDBid 1CHC_A)
HIT domain (PDBid 2YQQ_A) MYND domain (PDBid 3N71_A)
FYVE domain (PDBid 1VFY_A)
SPECIFIC VARIATIONS OBSERVED
E3 ligase RBX1 (PDBid 1LDJ_B)
RAG1 (PDBid 1RMD_A1,2,3)
UBR-box domain (PDBid 3NY3_A)
C1 domain of Rho-associated protein kinase 2 (PDBid 2ROW_A)
PDI-like Hypothetical Protein At1g60420 (PDBid 1V5N_A)
Lysine-specific demethylase 2A (PDBid 4BBQ_A3,4)
ASH2L (PDBid 3S32_A; N-terminal first zinc-binding site deteriorated in structure)
CW domain (PDBid 2L7P_A)
Zinc-binding region of E7 oncoprotein (PDBid 2EWL_A)
Treble clef of thymidine kinase (PDBid 2J9R_A)
YHS domain (PDBid 3U52_A)
Treble clef in KDM 5B (PDBid 5A1F_A)
Treble clef in FusB-like proteins (PDBid 2YB5)
MerB (PDBid 3F0P_A1)
MerB (PDBid 3F0P_A2)
Binuclear treble clef domain in Lysine-Specific Histone Demethylase 1b (PDBid 4FWE_A1,2)
Bacteriophage λ Ea8.5-like treble clef (PDBid 2M7A_A)
Homeodomain
RBP10 (PDBid 1I3Q_J)
three helical bundle
Zinc finger region of E6 oncoprotein TC_2 (PDBid 4GIZ_C2)
lambda cro repressor fold
Hypothetical protein ORF126 (PDBid 2X5R_A)
C-terminal sub-domain of ATP-grasp fold
T. thermophilus prolyl tRNA synthetase treble clef (PDBid 1H4Q_A)
IF3-like fold
Zinc-binding site in N-terminal domain of CasA/Cse1 subunit of E. coli CRISPR (PDBid 4TVX_I)
large insertion; mostly random coils with few SSEs
Dimerization domain in the Fur family (PDBid 2FE3_A)
SAP30-like treble clef (PDBid 2KDP_A)
PARP-like treble clef (PDBid 3OD8_A)
