# Supplementary material for: Classification of the treble clef zinc finger: noteworthy lessons for structure and function evolution
Source: Sci Rep. 2016 Aug 26;6:32070. doi: 10.1038/srep32070 (PMC4999995; doi:10.1038/srep32070)
Supplement: Supplementary File 1 [file srep32070-s2.pdf]

Title:

Classification of the treble clef zinc finger: noteworthy lessons for structure and function evolution

Authors:

Gurmeet Kaur, Srikrishna Subramanian[1]

Contact Information

CSIR-Institute of Microbial Technology (IMTECH), Sector 39-A, Chandigarh, 160036, India. Phone: +911726665483; Fax: +91-1722695215; Email: krishna@imtech.res.in

[1] Corresponding author

Supplementary File 2

Description of all families of the treble clef fold group.

This file contains the detailed description of all families, structural and functional features, and structure-based multiple sequence alignments.

1. TRASH family

Trafficking, Resistance and Sensing of Heavy metals (TRASH) family consists of metallochaperone-like treble clef (TC) domains that assist in the aforementioned functions [1, 2]. It includes MYM-type ZF (PDBid 2DAS_A), YHS domain (PDBid 1T0Q_A), DNA gyrase inhibitor YacG (PDBid 1LV3_A), ribosomal protein L24 (PDBid 1JJ2_T), HIT domain (PDBid 2YQQ_A) and MYND domain (PDBid 2JW6_A) (Pfam clan CL0175) (**Supplementary Figure i**). L24 was placed in the nuclear receptor-like finger family in the previous evolutionary classification of ZFs [3] but based on sequence similarity is grouped under the TRASH family in the current classification. This family has both mononuclear and binuclear TC folds. The latter include the HIT and MYND domains and few of these have a kink in α-helix near the second zinc-binding site (PDBid 3QWV_A, 3OXG_A).

The structural fold seen in NosL (PDBid 2HPU_A) and MerB (PDBid 3F0P_A) was recently shown to have evolved by the duplication and fusion of TRASH-like TCs [4]. The TCs from hypothetical protein TA0938 (PDBid 2FQH_A), Jann_2411 (PDBid 3H0N_A) and transcription protein Rtr1 (PDBid 4FC8_A) have also been included in this family.

The structure of TA0938 (PDBid 2FQH_A) is suggested to have a novel fold with a putative zinc-binding site based on the presence of four metal-chelating residues [5], but no metal ion is present in the structure. These metal-chelating residues are positioned on loops whose topological arrangement resembles a TC ZF. HHpred initiated with the TC region finds matches to TRASH family proteins (for example, YHS domain, PDBid 1MTY_D, E-value=1.6E-08), thereby arguing for its placement in this family.

Jann_2411 (PDBid 3H0N_A; PF11706: zf-CGNR; DUF1470) has a TC at its C-terminal that is predicted to function in nucleic acid interactions [6]. This TC finds matches to TRASH family members in sequence similarity searches. For example, HHpred finds MYM-type domain (PDBid 2L8E_A) with E-value=0.0093 and 23% sequence identity. MYM-type TRASH domain (PDBid 2L8E_A) and Jann_2411 TC could be superimposed with an RMSD=1.1 Å over 27 pairs of backbone C_α atoms.

Likewise, Dali search with TC of transcription factor Rtr1 (PDBid 4FC8_A) retrieved only MYM-type domain (PDBid 2W0T_A; Z-score=1.8, RMSD=2.2 Å, nali=29) and FFAS search with the TC domain of Rtr1 (PDBid 4FC8_A) found matches to TRASH proteins, although the score is below threshold (for example, PDBid 3H0N_A, Score= -7.77; PDBid 3DHG_A, Score= -6.23). Thus, based on these results, zinc finger domains (ZFDs) of Jan_2411 and Rtr1 have been provisionally placed in this family.

Functions performed

TRASH domain of the P-type copper transport ATPases and of MerH mediates trafficking of heavy metals [1, 7]. The FCS-motif containing human polycomb group protein lethal(3)malignant brain tumor-like 2 (PDBid 2W0T_A) and members of the DUF581 family are proposed to function in nucleic acid- and protein-interactions, respectively [8, 9]. α-helix of L24 is seen to interact with 23s rRNA [10]. The HIT and MYND domains are suggested to function as protein interacting modules [11, 12].

NosL is a proposed to function as a putative copper metallochaperone [13] and MerB is an enzyme [14] that has evolved a catalytic function on the structural zinc binding site [4].

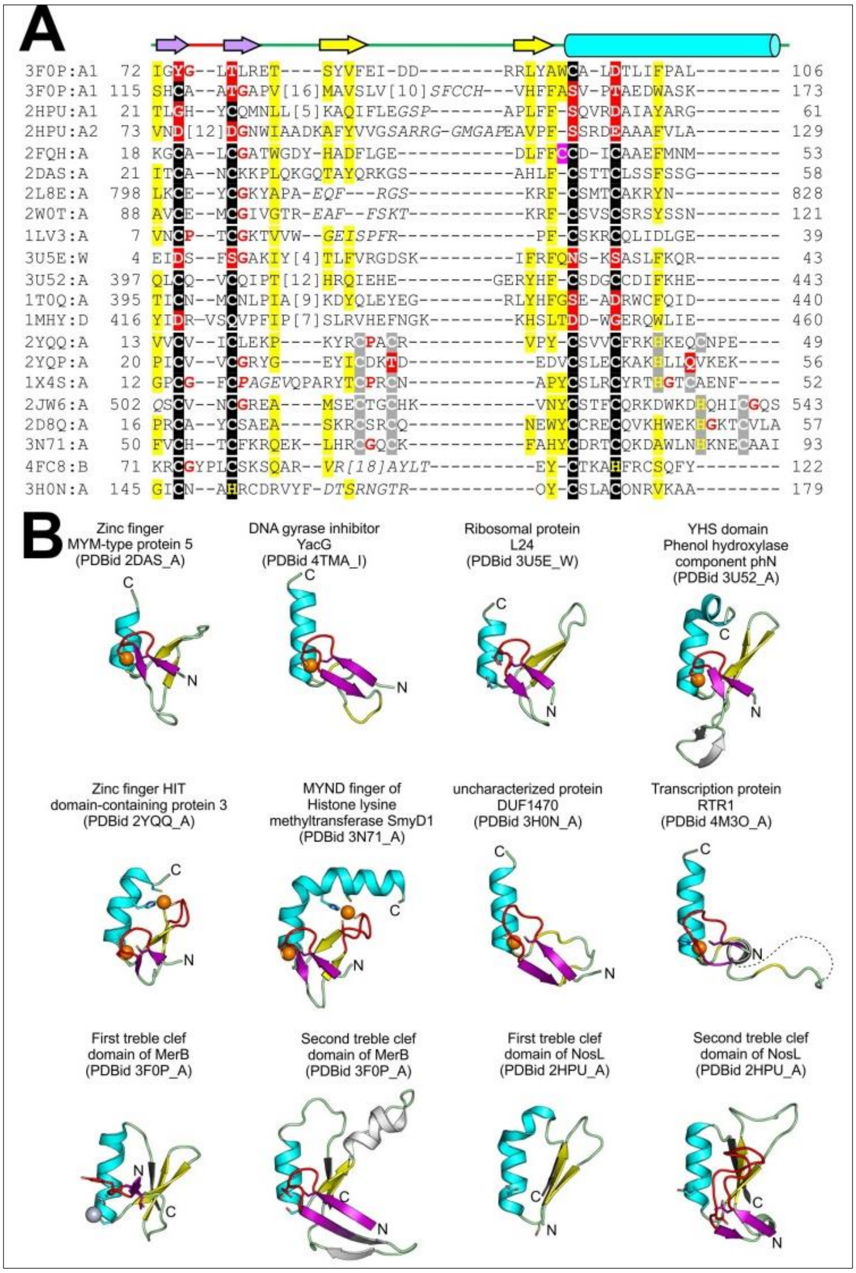

Supplementary Figure i: TRASH domain family.

(A) Structure-based multiple sequence alignment (MSA) of TRASH family. PDBid, start and end aminoacid numbers are indicated for each sequence. Secondary structure diagram is depicted above the alignment. The zinc-binding residues (Cys/His) are highlighted in black and other aminoacids at equivalent position in red. Potential metal-chelating residues are boxed in magenta. The zinc-chelating residues at the second zinc-binding site in binuclear treble clefs are highlighted in grey. Regions where the structures are not superimposable are shown in italics. Small aminoacids (Gly, Pro) in the vicinity of the zinc-binding ligands are coloured red. Uncharged residues (all aminoacids except Asp, Glu, Lys and Arg) in mostly hydrophobic sites are highlighted yellow. Conserved aromatic residues have been highlighted cyan. Long insertions are not shown and the number of omitted residues is boxed in green. Regions of circular permutations are separated by '|' coloured in blue and residue numbers along the permuted regions are coloured in red. This colouring scheme for MSA is followed all through this document. The sequences used for alignment were obtained after clustering all TRASH domains in the dataset at 40 %ID. **(B)** Ribbon diagrams of TRASH family representatives. Colouring scheme follows that used in Figure 1 and Supplementary Figure S1.

2. RING-like treble clef zinc fingers

RING domain, identified in human *RING1* [15], is also referred as C3HC4 ZF or the 'A-Box' [16]. The cumulative number of RING proteins in human is estimated to be more than kinases [17] and this family is one of the most populated TC families (Pfam clan CL0229, SCOPid 57849). The structure of RING domains consists of an N-terminal mononuclear TC followed by a 'squiggle' and a C-terminal β-strand [18]. The latter forms a three-stranded β-sheet with the primary β-hairpin. The extensions to the core of the TC and the turn of the primary β-hairpin provide additional ligands to chelate a second zinc ion. In the bonafide RING domains, the tertiary structure of the second zinc site resembles a rubredoxin-like zinc ribbon [19]. Domains such as the B-box, ZZ, TFIIH-p44, Zf-UBP, and C1-type are suggested to be evolutionarily related to the RING domain based on two characteristic structural features, *viz.*, a three-stranded β-sheet consisting of the primary β-hairpin and the C-terminal β-strand, and the invariable location of the last metal-chelating residue at the beginning of the C-terminal β-strand [18].

RING-like family in this classification groups the bonafide RING domains, U-box, B-box, UBP-type, UPF1-type, UBR-box, histone acetyltransferase P300/CREB-binding protein, ArfGAP/RecO-like ZF, binuclear TC domain of Replicase polyprotein 1ab, the TFIIIC τ 60KDa subunit RING domain, the α-COP RING domain, ZZ domains, cysteine-rich domains (CRDs) of At1g60420 and TFIIH-p44, C1 domains of protein kinases and the TC domain in lysine-specific histone demethylase (KDM) (**Supplementary Figure ii**). The reason for including B-box, C1 and related domains in the RING-like family is discussed in the main manuscript.

The binuclear TC domain of Replicase polyprotein 1ab (PDBid 4N0N_A) possesses the two characteristics of the RING domains and we observe that this domain also has similar co-occurring domains as the UPF1 (PDBid 2XZL_A), i.e. in both these proteins, the RING-like domain is followed by a mononuclear TC and a helicase domain.

The TATA-binding protein-interacting region of τ 60 kDa subunit of TFIIIC (PDBid 2J04_A) has a novel α/β fold [20], that Pfam annotates as 'Putative zinc-finger of transcription factor IIIC complex' (PF12660). Our structural analysis reveals the presence a zinc ribbon followed by a RING domain in this region. Several homologs of TFIIIC τ 60kDa subunit are seen to possess metal-chelating residues at positions that recapitulate the position in bonafide zinc ribbons and RING fingers (for example, UniProtIDs Q4WLA9, Q2TY38 have 6 and 7 out of the 8 zinc-binding residues, respectively).

We have identified a RING-like domain at the C-terminal of α-COP (cytoplasmic coat protein) (PDBids 3MV3_A, 3MKR_B) [21]. Sequence analysis reveals strong conservation for the second metal ion chelating aminoacids, but residues coordinating the first metal ion are substituted in homologs. This pattern of metal-chelating residue conservation is similar to that seen in the RING-like domain of TFIIIC τ 60.

Structural variations

The U-box domains, though structurally similar to RING domains, do not chelate metal ions and the resulting fold is stabilized by hydrogen bonds and salt bridges [22]. Loss of zinc-binding is also seen in UBP domains from ubiquitin carboxyl-terminal hydrolase 5 (PDBid 2G45_A; second zinc-binding site absent), pre-mRNA-splicing factor SAD1 (PDBid 4MSX_A; first zinc-binding site absent) and KDMs (PDBids 3AVR_A, 3ZLI_A; second zinc-binding site absent). Only a single metal-binding site corresponding to that of mononuclear TCs is seen in histone acetyltransferase P300/CREB-binding protein and ArfGAP/RecO-like RING domains.

The B-box, UPF1-type and TC domain of KDMs have extended regions after the α-helix that contributes ligand(s) to bind the second metal ion but lack the typical 'squiggle'. RING domains of E3 ubiquitin-protein ligase RBX1 and RBX2 (PDBids 1LDJ_B, 2ECL_A, respectively) have a nested zinc-binding loop inserted in between the zinc knuckle and primary β-hairpin (**Supplementary Figure ii, iii**). RAG1 protein (PDBid 1RMD_A) and UBR-box (PDBid 3NIH_A) have an additional zinc ion that shares one of its zinc ligand with the first zinc-binding site of the RING finger, forming a metal-ion cluster.

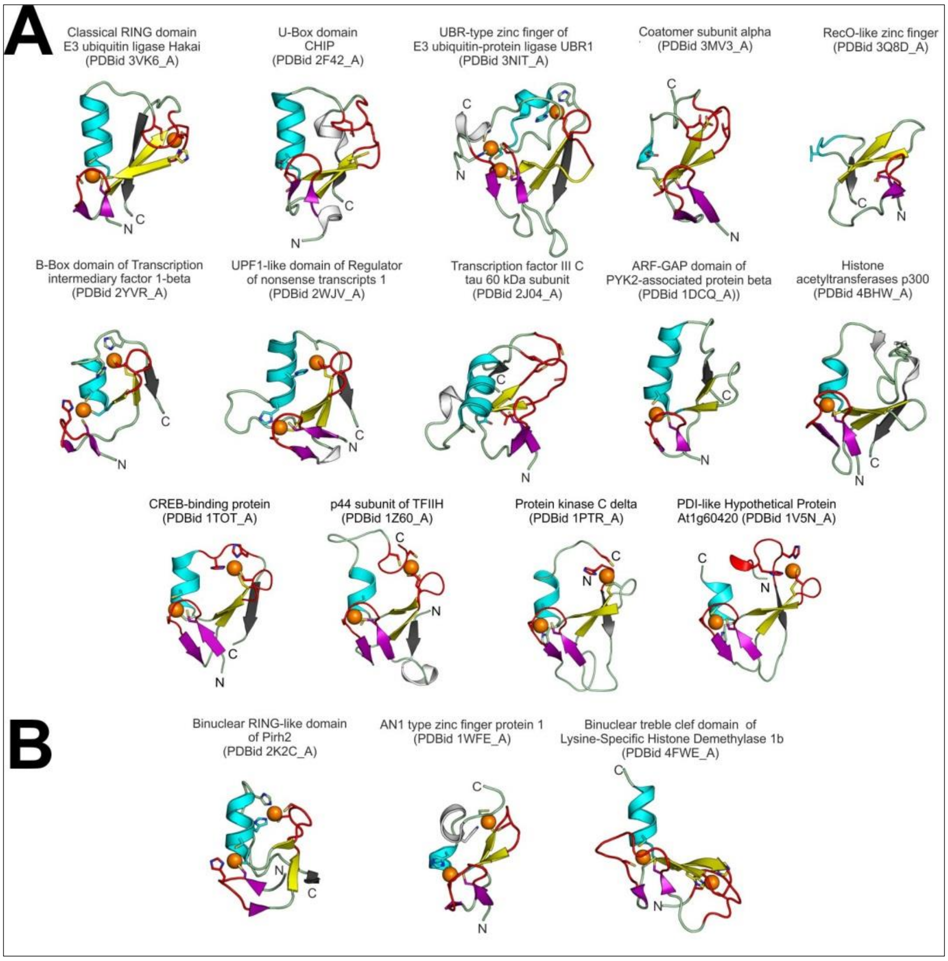

Supplementary Figure ii: RING-like family and some other binuclear treble clefs.
(A) Ribbon diagrams of RING-like family representatives. **(B)** Some other binuclear treble clefs.

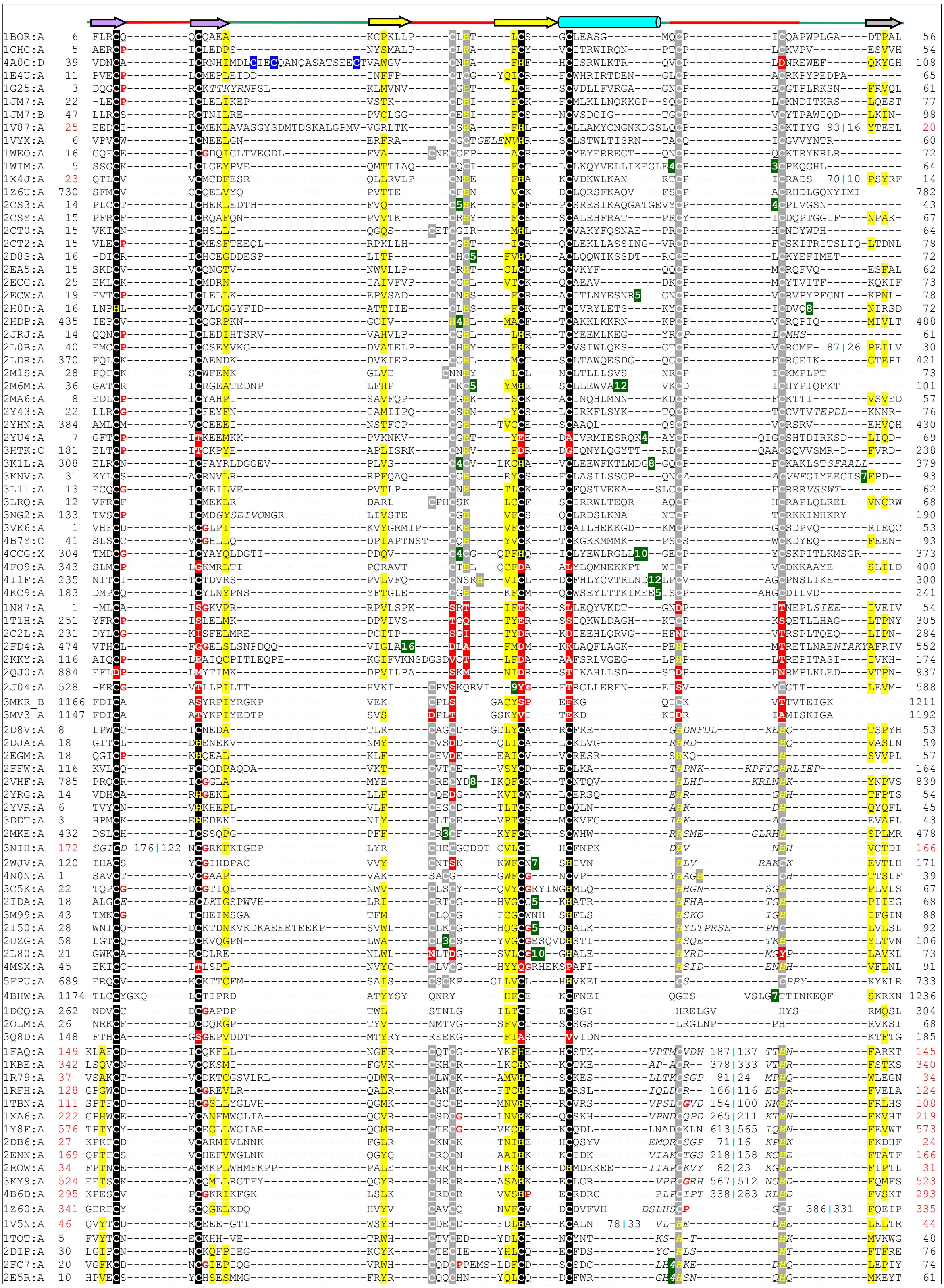

Supplementary Figure iii: Structure-based MSA of RING-like family.
The metal-chelating residues for the second zinc ion are boxed in grey (in blue for a third zinc ion present in some proteins). The sequences used for alignment were obtained after clustering all sequences of the family in the dataset at 40 %ID.

TFIIIC τ 60kDa subunit has an additional α-helix in between the primary β-hairpin and α-helix of the TC (PDBid 2J04_A). This insertion is of variable length in homologs that leads to poor sequence alignment in this region. In histone acetyltransferase p300/CREB-binding protein (PDBid

4BHW_A), a larger β-hairpin replaces the 'squiggle'. The UBR-box (PDBid 3NIH_A) has a unique circularly permutation at the zinc knuckle and additional zinc ion bound by N- and C-terminal extensions [23]. The C1 domain, and CRDs of At1g60420 and TFIIH-p44, too have circularly permutations but at the second zinc-binding site of the RING-like core.

A binuclear form of the TC domain inserted in the α-helical domain proceeding the JmjC domain in lysine-specific demethylase (KDM) 5B (PDBids 5A1F_A, 5FPL_A) is seen in some recently determined structures. Until before these, all available structures of this TC domain, *viz.* from KDM 6A (PDBid 3AVR_A), KDM6B (PDBid 4EYU_A) and UTY (PDBid 3ZLI_A) had only a single zinc ion. However, the presence of an additional zinc ion in some members and a conserved C-terminal β-strand in all known TC structures of this protein family, argues for classifying them as members of the RING-like TC family. It is likely that this TC domain has diverged from a RING-like precursor during expansion of chromatin-related functions in eukaryotes similar to some other ZF domains [23, 24].

Functions performed

RING domains interact with other proteins by utilizing different regions of the fold [18, 25]. RING domains in E3 ubiquitin ligases facilitate the transfer of ubiquitin from E2 ligases to the target by interacting with E2-ubiquitin conjugate [17, 18, 25]. RING fingers also function as E3 SUMO ligases (PDBid 3HTK_A) and stabilize the E2-SUMO-substrate complexes [26]. MDM4's RING domain helps in regulating the E3 ligase activity of MDM2 [27]. RING domain of RNF168 helps in extending the ubiquitination initiated by the RING domain of RNF8 [28]. U-box also exhibits E3 ubiquitin ligase activity (PDBid 2QJ0_A) [29].

RING domains also help in oligomerization and formation of macromolecular assemblages [25]. Two different interfaces of the RING domain are seen involved in dimerization, *viz.* the three-stranded β-sheet of the RING finger as seen in the heterodimeric MDM proteins (PDBid 2VJE) or α-helices that precede and follow the RING domains as seen in the heterodimer of BRCA1 and BARD1 (PDBid 1JM7) and homodimer of RAD18 (PDBid 2Y43). RING domain of cellulose synthase is also suggested in formation of higher-order complexes [30].

The RING-like domain of UPF1 with its proceeding mononuclear TC mediates interactions with UPF2 (PDBid 2WJV) [31]. The UBP-type domains bind to ubiquitin tails (PDBids 3PHD, 2G45) [32], although in some proteins with a UBP-related domain, such as pre-mRNA-splicing factor SAD1 (PDBid 4MSX_A), the ubiquitin-interacting motif is mutated [33]. UBR-box interacts with the N-degron containing proteins that have to be targeted for degradation (PDBid 3NIH) via the β-strand of the split β-hairpin containing zinc-knuckle [34]. RING domain of p300/CREB-binding protein (PDBid 4BHW_A3) lacks E3 ligase activity and is involved in occluding the active site of histone acetyltransferase domain [35]. TC domain of KDMs and the preceding jumonji domain help in interacting with the histone tail and thereby achieving site-specific histone demethylation (PDBid 3AVR_A) [36].

The TC in RecO is suggested to play a role in interacting with DNA [37], whereas that in ADP ribosylation factor GTPase activating proteins (ArfGAPs) is proposed to mediate interactions with Arfs [38]. A conserved arginine residue, the 'arginine finger', from the α-helix of TC of ArfGAP completes the active site of the interacting Arf (PDBid 3LVR) [38, 39].

B-box domains also mediate protein-protein interactions [40] that have been implicated in determining the subcellular location of the TRIM and CYLD [41, 42]. B-box1 of Midline1 interacts with Alpha4 and has a putative E3 or E4 ubiquitin ligase function [40, 43]. B-box2 of Midline2, on the other hand, regulates the E3 ligase activity of the co-occurring RING domain and interaction between the B-box1 and Alpha4 [40, 43]. The B-box domain from CPEB1 has been proposed to interact with RNA and proteins [44, 45].

C1 domains were discovered in PKC for their ability to bind diacylglycerol (DAG) and phorbol esters (PE), a lipid secondary messenger and its tumor promoting analog, respectively [46], These are now seen to be present in a number of proteins and are termed either conventional, novel or atypical based on their abilities to interact with DAG or PE [47]. DAG/PE bind and 'unzip' a region of the knuckle containing β-hairpin, that helps expose a large hydrophobic surface on the C1 domains that insert into lipophilic membranes [48]. Other than mediating interactions with the lipid molecules, C1 domains also mediate protein-protein interactions, for example, in the interaction of Vav proteins with RhoGTPases [49]. The CRD of TFIIH p44 is suggested to interact with p34 subunit [50].

3. Binuclear TC of lysine-specific histone demethylase 1b

The binuclear TC of lysine-specific histone demethylase 1b (PDBid 4FWE_A) is structurally dissimilar to other binuclear TCs [51], as the second half-site for binding the second zinc ion is provided by a structurally non-superimposable extended region at the N-terminal of the TC and not from the C-terminal (**Supplementary Figure iiB**). This binuclear TC is suggested to be important for stability and demethylase activity of the protein [51, 52].

4. Binuclear TC of p53-induced RING-H2 (Pirh2) protein or Rchy1

The N-terminal cross-braced binuclear TC of Pirh2 (PDBid 2K2C_A, **Supplementary Figure iiB**) is structurally similar to the RING-like domains as suggested previously [53]. However, these do not share significant sequence similarity with other RING-like domains and also lacks E3 ligase activity [53].

5. AN1 type zinc finger

AN1 is a binuclear TC domain with the last pair of metal-chelating aminoacids contributed from a region with extended conformation at the C-terminal of the TC (PDBid 1X4V_A) (**Supplementary Figure iiB, iv**). The AN1 domain in SAP interacts with TRAF6 protein, thereby regulating the immune response [54].

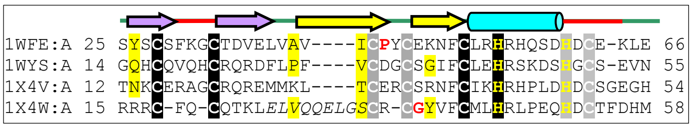

Supplementary Figure iv: Structure-based MSA of AN1 family.
The sequences used for alignment were obtained after clustering all AN1 domains in the dataset at 40 %ID.

6. N-terminal domain of Ada repair protein

The N-terminal domain of DNA-repair protein Ada has a four-stranded β-sheet surrounded by two α-helices and a zinc ion [55-57] (**Supplementary Figure vE**). The zinc-binding site is essential not only for proper folding of the domain but also for the methyl phosphotriester lesion repair in DNA [56], as one of the zinc-chelating cysteines is a nucleophilic methyl acceptor from the damaged-DNA onto itself [58]. The zinc-binding domain of Ada was not considered a bonafide ZF based on the reactive nature of the zinc site and thus, excluded from the previous classification [3]. However, it is included in the present classification based on our current understanding that the structural zinc-binding sites may be reactive in proteins, for example in C1 domain of PKCα [59], or may evolve novel functional attributes during evolution as seen in MerB [4].

The structure of zinc-binding domain of Ada (PDBid 1U8B_A) superimposes well with the Arf-GAP TC (PDBid 1DCQ_A; RMSD=1.9 Å over 27 pairs of equivalent backbone C_{α}) and RecO ZF (PDBid 3Q8D_A; RMSD=2.0 Å over 25 pairs of equivalent backbone C_{α}), but a circular permutation at the zinc knuckle containing β-hairpin needs to be considered (**Supplementary Figure vE**). TC of Ada, like RecO, has an ill-defined α-helix but a three-stranded antiparallel β-strand encompassing the primary β-hairpin is similar to the arrangement seen in the RING-like domains.

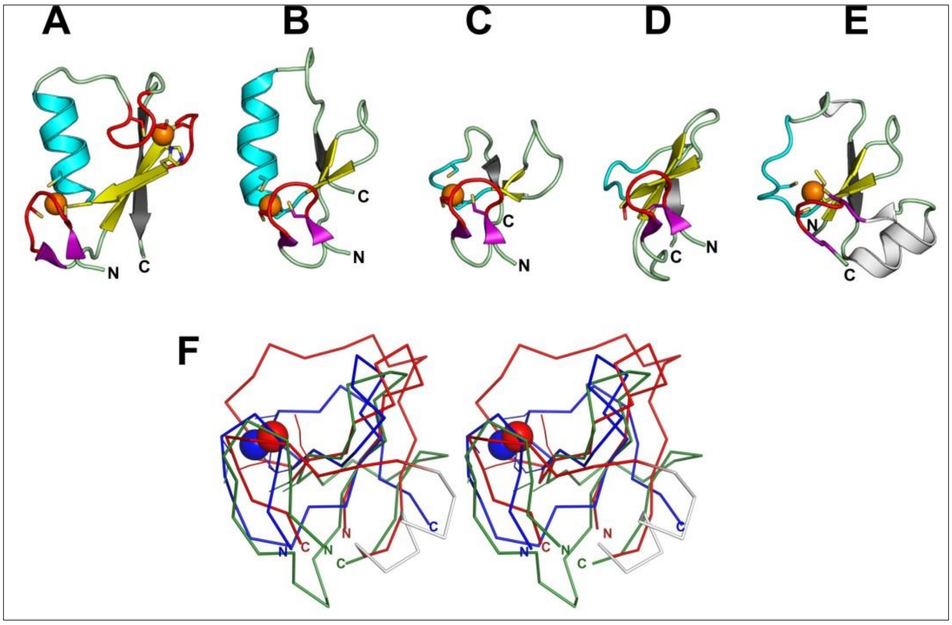

Supplementary Figure v: Comparison of zinc-binding domain of Ada with other treble clefs.
(A) RING domain (PDBid 3VK6). **(B)** Arf-GAP treble clef (PDBid 1DCQ). **(C)** RecO treble clef (PDBid 1U5K_A). **(D)** degraded RecO treble clef (PDBid 3Q8D_B). **(E)** Ada treble clef (PDBid 1U8B_A). **(F)** Stereo diagram for superimposition of RecO and Ada treble clef domains (RecO, PDBid 3Q8D_B: green, PDBid 1U5K_A: blue; Ada, PDBid 1U5K_B: red).

7. Phosphatidylinositol-3-phosphate binding domain family

This family encompasses the FYVE-like and the PHD-like cross-braced binuclear TC domains (Pfam clan CL0390, SCOPid 57902) (**Supplementary Figure vi**). The mononuclear TC is extended at the C-terminal by an additional α-helix that along with the turn of the primary β-hairpin chelates the second zinc ion. We include the binuclear domain from the C-terminal cysteine-rich domain of E3 ligase CHFR (PDBid 2XOC, residues 482-620) in this family as it shows significant structural similarity of the zinc-binding sites with FYVE-like TCs.

Structural variations

The region beyond the α-helix of the TC domain that contributes one of the zinc-chelating half-sites of the second zinc-binding site is seen to be variable in the PHDs and may take up an extended conformation [60]. The approximate location of the second zinc ion and first ligand of the last zinc-coordinating pair in the PHD fingers is similar to other binuclear TCs like RING, B-box, ZZ, etc. [18]. FYVE-like ZFDs have an additional β-hairpin in between the additional C-terminal α-helix and the α-helix of the TC. The FYVE ZF fold is proposed to have convergently achieved a binuclear state independent of other TCs, involving evolutionary events like duplication of mononuclear TCs followed by circular permutation [18].

The FYVE-like ZFD of CHFR (PDBid 2XOC, residues 482-620) has large insertions in between the half-sites of both zinc-binding sites. An additional third zinc ion is seen chelated by ligands present in a loop just after the α-helix of TC and shares one of its zinc-chelating residues with those chelating the zinc ion of the TC resulting in a metal-ion cluster similar to the one observed in RAG1 (PDBid 1RMD_A) and UBR-box (PDBid 3NIH_A).

The ZFD of Ash2L protein (PDBids 3S32, 3RSN) is referred to as an 'atypical PHD' finger as it lacks the first zinc ion and the region corresponding to the zinc knuckle is disordered. Only the SSEs involved in binding the second zinc ion are seen in the structures, and thus, appear atypical and resemble a CW-type domain (described below). However, sequence-based analysis of Ash2L homologs suggests that Ash2L is a classical PHD finger that has lost its first zinc-binding site ligands in some homologs including the ones that are structurally characterised. FFAS based sequence similarity search initiated with the PHD of Ash2L finds matches to bonafide PHD fingers (for example, PHD finger protein 2, PDBid 3KQI_A, Score= -29.4; Histone lysine demethylase PHF8, PDBid 1WEP_A, Score= -28.3).

The second PHD domain of Bromodomain and PHD finger-containing (BRPF) protein 2 (PDBid 2LQ6_A) has an additional functionally important β-hairpin inserted between the α-helix of the TC and the C-terminal α-helix.

Functions performed

FYVE domains bind phosphatidylinositol 3-phosphate (PI3P), a rare lipid on endosomal and phagosomal membranes, thereby, facilitating vacuolar protein sorting and endosomal functions [61, 62]. A conserved basic motif [R(R/K)HHCR] on the second β-strand of the primary β-hairpin forms a

pocket for interacting with the phosphate and hydroxyl groups of PI3P [62]. Two other conserved motifs, *viz.* WXXD (X is any aminoacid) and RVC at the N- and C-terminal, respectively, also contribute to PI3P binding [62]. Cysteine in the RVC motif is actually one of the zinc binding residues of FYVEs. Further, an extended region in between the knuckle and the primary β-hairpin, also called the 'turret', with conserved hydrophobic residues is proposed to insert into the lipid bilayer [61].

FYVE domain from RIM2α protein is shown to function as a protein-protein interaction mediator (PDBid 2CJS_B) [63]. Likewise, the C-terminal cysteine-rich region with the FYVE-like ZFD of CHFR interacts with substrate proteins to be ubiquitinylated [64, 65].

PHD ZFD is one of the few protein domains that can read epigenetic marks on histone tails [66] and modulate cellular functions such as chromatin remodelling, signalling, VDJ recombination, transcription activation and repression. PHD fingers identify diverse histone modifications such as H3K4me3/2, H3K4me0 (unmodified), H3K9me3, H3K4ac, H3R2 and acetylated H4 [67, 68]. Structural analysis suggests that the PHD ZFs employ the first β-strand of the primary β-hairpin for interacting with unmodified and methylated histones tails. The histone tail forms a three-stranded β-sheet with the primary β-hairpin [67]. An 'aromatic cage' facilitates the binding of trimethylammonium group on K4 of H3K4me3 (PDBid 2RI7), whereas the H3R2 binding pocket is rich in acidic and polar aminoacids (PDBid 4GY5) [69, 70]. The acetylated lysine in the histone tail is, however, bound in a narrow groove that lines the second β-strand of the primary β-hairpin (PDBid 2KWJ) [71]. The 'reader' properties of PHD fingers for histones are known to be affected by post-translational modification status of neighbouring aminoacids. Sometimes, neighbouring domains also affect the reading of the histone by the PHD fingers, a phenomenon termed as 'module integration' [68]. For example, the first and second PHD fingers of DPF3b simultaneously recognize K14ac and K4me0 marks on histone H3, respectively (PDBid 2KWJ).

Few PHD domains interact with other proteins too, besides histones. For example, different surfaces of the PHD finger from Pygopus (PDBid 2XB1) interact with histone tails and homology domain 1 of BCL9 [72], thereby linking the histone reading process to cellular signalling. PHD domains of KAP1 and SIZ1 are shown to possess E3 SUMO-ligase activities [73]. PHD finger of KAP1 interacts with Ubc9 E2 ligase that causes SUMOylation of the adjacent bromodomain leading to transcriptional repression [73].

Interaction of PHD domain with nucleic acids has also been reported. In, BRFP2, residues from the extra β-hairpin and primary β-hairpin are suggested to bind DNA non-specifically [74]. Some contribution of residues on the second PHD finger of plant homeodomain finger 6 (PHF6; PDBid 4NN2) towards DNA binding is also reported [75]. Like BRFP2, PHF6 also has a similar extra β-hairpin, although the residues implicated in DNA binding are not located at similar positions.

8. CW domain family

CW domain of many chromatin-associated proteins [76] has a structure consisting of a β-hairpin, followed by a α-helix and a C-terminal region with extended conformation (PDBid 4GUS_A). A structural zinc ion is bound by the turn of the β-hairpin and the C-terminal region (**Supplementary Figure vi**).

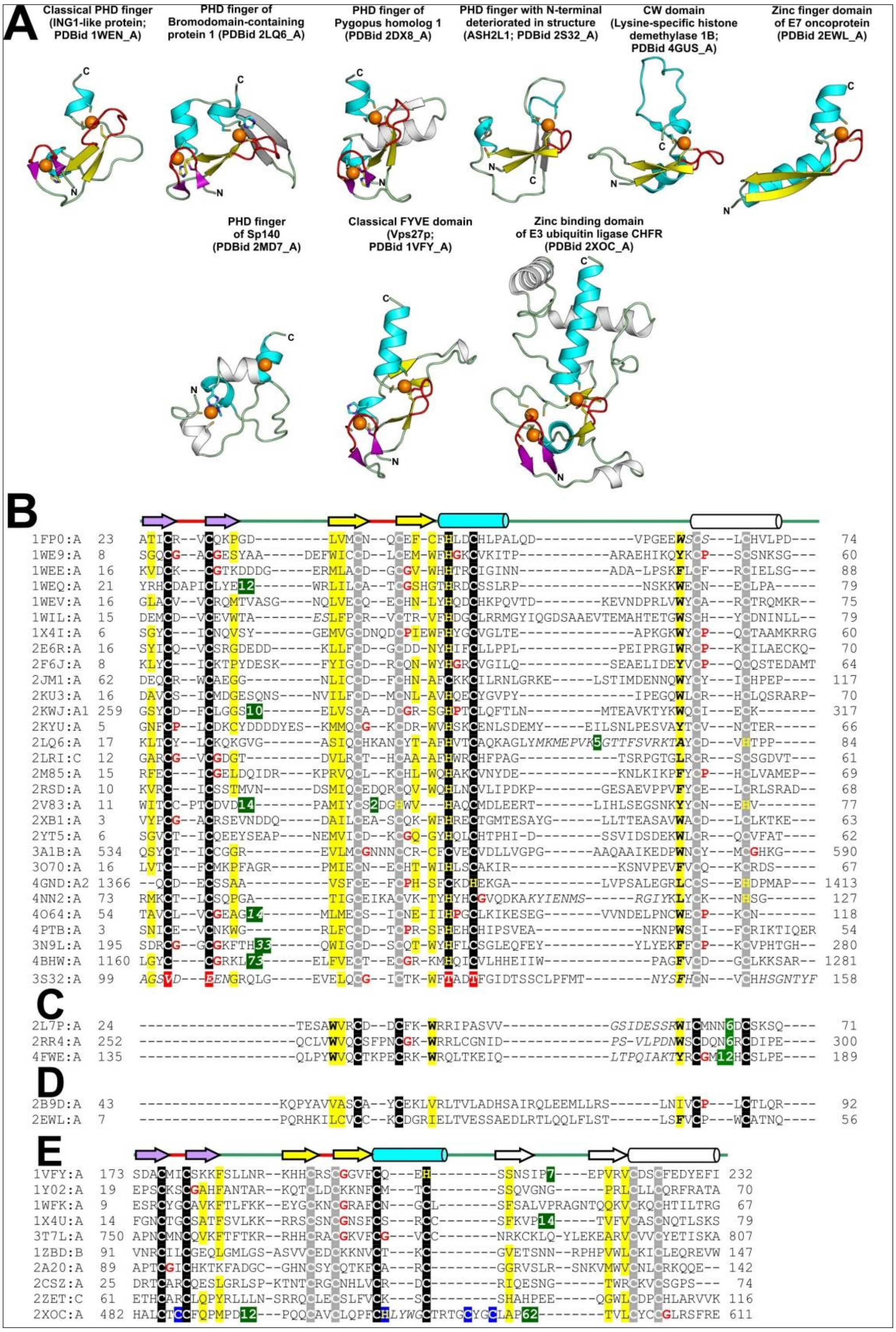

Supplementary Figure vi: Phosphatidylinositol-3-phosphate binding domain, CW domain and E7 ZF families. **(A)** Ribbon diagrams of PHD-like ZFDs, CW domain, E7 ZF and FYVE-like ZFDs. **(B)** Structure-based MSA of the PHD-like domains. **(C)** Structure-based MSA of the CW domains. **(D)** Structure-based MSA of the E7 ZFDs. **(E)** Structure-based MSA of the FYVE-like domains. The sequences used for alignment were obtained after clustering all sequences of the family in the dataset at 40 %ID; for E6 protein the sequences of the two structurally-characterised proteins are used.

CW domain derives its name from conserved cysteine and tryptophan residues (PF07496) and is structurally related to PHD fingers [76, 77]. The structure of CW domains is equivalent to the second zinc-binding region of PHD fingers, although the spacing between the last zinc-binding ligand pair is greater in CW domains. The CW domain lacks the first zinc-binding site of the PHD fingers [76] but the mode of interaction of CW domain with histone tail (PDBid 2RR4) is similar to that seen in PHD fingers, wherein the histone tail forms a three-stranded β-sheet with the primary β-hairpin [77]. The aromatic cage, as seen in the PHD fingers, is likewise employed by the CW domains to interact with the histones [77]. CW domain of lysine-specific demethylase D2 (PDBid 4FWE_A3), however, is suggested lack histone-interacting ability [51].

9. Zinc-binding region of E7 oncoprotein

The zinc-binding region of papillomavirus E7 oncoprotein (PDBid 2EWL_A) is shown previously to have derived from a PHD-like binuclear TC during evolution [78]. E7 also completely lacks the first zinc-binding site of the PHD-like TCs similar to CW domains and possesses only a β-hairpin (equivalent to the primary β-hairpin) and two α-helices (equivalent to the region after the primary β-hairpin) [78]. Ligands from the turn of the β-hairpin and the N-terminal region of the last α-helix chelate a single zinc ion (**Supplementary Figure vi**).

This zinc-binding region (also referred to as conserved region 3, CR3) of E7 is suggested to help in dimerization and interacting with host proteins such as Rb and histone acetyltransferase CBP/p300 [78-80]. Conserved hydrophobic and charged residues on the β-strands and proceeding α-helix are implicated in these functions [79, 80].

10. Nuclear receptor-like finger family

TCs of nucleic acid receptor DNA-binding domain (DBD) (PDBid 2HAN_A), LIM domains (PDBid 1IML_A), Stc1 (PDBid 2LUY), PARP (PDBid 1V9X) and DNA Ligase (PDBid 1UW0), endonuclease VIII and endonuclease VIII-like proteins (PDBids 1K3W_A, 1TDH_A), intron endonuclease I-TevI (PDBid 1I3J_A), restriction endonuclease Hpy99I (PDBid 3FC3), restriction endonuclease PacI (PDBid 3LDY), DNA glycosylase (PDBid 1L1T_A), C-terminal domain of Ile-tRNA synthetase (PDBid 1FFY_A), nucleotide excision repair protein XPA (PDBid 1XPA_A), DskA and FmdE (PDBids 1TJL, 2GVI), first TC of tRNA thiouridine synthetase TtuA (PDBid 3VRH_A), GATA DNA-binding TC ZFs (PDBid 3DFX_A), GATA-like finger in ADD domain (PDBids 2JM1_A1, 2PV0_A1) and PHD finger protein 6 (PDBid 4NN2_A1), Four and a half LIM domains protein 2 (PDBid 2MIU) and ribosomal proteins S14 (PDBid 3U5C_d), S26 (PDBid 3U5C_a), L28 (PDBid 2JZ6_A), L31 (PDBid 2HGJ_3), L34 (PDBid 3U5E_g) and S21 (PDBid 1VW9_W) are included in this family (**Supplementary Figure vii**). TCs of this family mostly function in nucleic acid binding, although some, like the LIM domain mediate protein-protein interactions. Pfam has misclassified some TC domains of this family, for example, Prokaryotic dksA/traR C4-type zinc

finger family (PF01258), FPG IleRS zinc finger (PF06827) and GATA zinc finger (PF00320) under the Zn_Beta_Ribbon clan (CL0167).

Structural variations

DBD of nuclear receptor proteins comprises of two TCs, that have probably evolved by duplication [3, 19], although the first zinc-binding half-site of the second TC is oriented differently as compared to bonafide TCs. LIM (Lin11, Isl-1 & Mec-3) domains also bind two zinc ions [81] and their fold consists of two tandem TCs with the α-helix of first being reduced to a single turn.

Evolutionary related TCs from PARP and DNA ligase have additional SSEs including an extra β-strand at the N- and C-terminal that form a four-stranded mixed β-sheet with the primary β-hairpin, and an additional α-helix present at the C-terminal runs roughly parallel to the α-helix of the TC. TCs from PARP have a longer primary β-hairpin with a variable region of interest at its turn [82]. DksA TC lacks a primary β-hairpin and has an α-helix instead. A paralog of DksA from *Pseudomonas aeruginosa,* DksA2 (PDBid 4IJJ), does not bind zinc as it has only two Cys that too are suggested to form a disulfide bond to stabilize the structure [83].

Stc1 (PDBid 2LUY) also has tandemly-duplicated TCs that interact with Argonaute 1, but unlike LIM domains, a longer linker joins the two TCs that allows for dynamic inter-domain motions [84]. The second TC domain of Stc1 finds matches to other TCs of this family, such as the restriction endonuclease HPY99I (PDBid 3GOX, E-value=0.00078) in HHpred.

Intron endonuclease I-TevI (PDBid 1I3J_A) has a very short TC in which the primary β-hairpin is replaced by a loop and the α-helix is reduced to a single turn [3].

Functions performed

The α-helix of the first TC of nuclear receptor DBD acts as the recognition helix and fits into the DNA major groove to provide specific interaction [85, 86]. Additional non-specific interactions are provided by the side-chains of basic aminoacids on the DBD and the phosphate groups in the DNA backbone [86].

PARP TCs bind to nicked-DNA via their primary β-hairpin (PDBids 3OD8, 3ODE) that interacts non-specifically with the phosphodiester backbone and the hydrophobic exposed surfaces of the nucleotide bases [82]. The TC of I-TevI interacts with DNA in the minor groove by forming non-specific hydrogen bonds with the phosphate backbone [87].

The first TC in His-Me nucleases Hpy99I (PDBid 3F3C_A) and PacI (PDBid 3LDY_A) is involved in DNA binding, along with the catalytic TC (classified in the His-Me family) [88]. The primary β-hairpin in Hpy99I (PDBid 3FC3) and the α-helix in PacI (PDBid 3LDY) provide residues that assist in DNA binding in the major groove. Likewise, the primary β-hairpin of the TC in endonuclease VIII (PDBid 2EA0) and DNA glycosylase (PDBid 1L1T) is involved in DNA binding [89].

In GATA-binding TCs, the α-helix and the region between the knuckle-β-hairpin and the primary β-hairpin is involved in interacting with the DNA major groove, for example in erythroid-

specific transcription factor (PDBid 1GAT) [90]. In the nitrogen regulatory protein (PDBid 2VUS), GATA TC is seen mediating dual interactions, i.e. with DNA and inhibitory protein NmrA, by utilizing the same interface (PDBids 2VUS, 4GAT).

LIM domains interact with a variety of proteins in different cellular processes like cytoskeletal organization, gene expression, oncogenesis and signal transduction [91]. LIM domains utilize different interfaces to interact with a diverse set of proteins. For example, in the co-crystal structure of LIM domain of Tes with an actin-related protein and Mena (PDBid 2XQN) [92], one of the interacting partner forms a β-zipper interaction with the first β-strand of the primary β-hairpins of the LIM TCs and the other is stacked on the opposite face that comprising of the zinc knuckle and regions of the second β-strand of the primary β-hairpin.

The TC of XPA is proposed to interact with RPA70 protein [93, 94]. Transcriptional regulator ATRX (PDBid 2JM1), DNA (cytosine-5)-methyltransferase 3A (DNMT3, PDBid 3A1B), DNA (cytosine-5)-methyltransferase 3-like (DNMT3L, PDBid 2PV0) and PHD finger protein 6 (PDBid 4NN2) have a GATA-like TC proceeded by a PHD domain. The GATA-like TC and the PHD ZF in ATRX, DNMT3 and DNMT3L, are together referred to as the ADD domain (**A**TRX-**D**NMT3-**D**NMT3L) (PDBids 2JM1, 2PV0), where the GATA-like finger is suggested to provide an extended surface for the specific binding of modified histone tails [68, 95, 96]. The GATA-like TC of PHD finger protein 6 (PDBid 4NN2), on the other hand, assists DNA binding [75].

11. SBT domain family

The tandem TCs of the DBD of squamosa promoter binding protein (PDBid 1UL4) constitute this family. These TCs help in mediating specific and non-specific interactions with DNA [97] (**Supplementary Figure vii**). In the second TC, three zinc-chelating residues are contributed by the knuckle and only one from the α-helix. The second TC (PDBid 1UL4) also finds a low-scoring match to the TC of polyhomeotic-like protein 1 (PDBid 2L8E, E-value=0.36) in HHpred search and these TCs could be manually superimposed with an RMSD=1.3Å over 24 pairs of backbone C_α atoms.

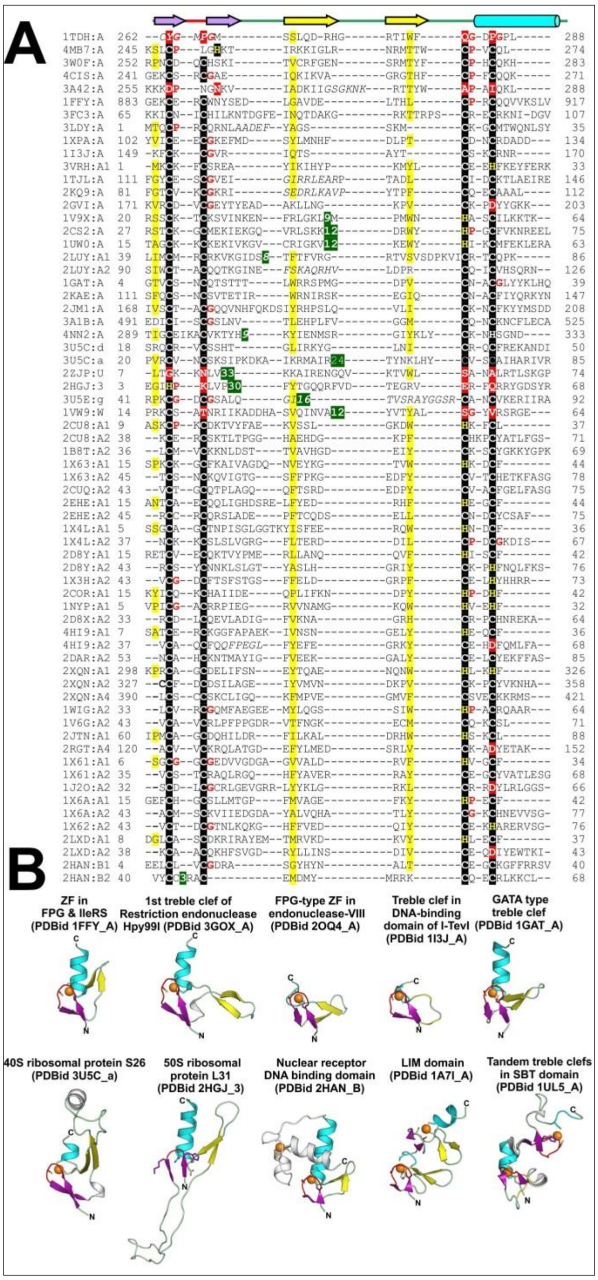

Supplementary Figure vii: Nuclear receptor-like finger family.

(A) Structure-based MSA of nuclear receptor-like finger family. The sequences used for alignment were obtained after clustering all sequences of the family in the dataset at 40 %ID. **(B)** Ribbon diagrams of nuclear receptor-like finger family representatives and the tandem treble clefs in SBT domain.

12. His-Me family

This family includes the catalytic TCs of His-Me nucleases (such as, Colicin E2, PDBid 3U43_B; intron-encoded homing endonuclease I-PpoI, PDBid 1CYQ_A; restriction endonuclease Hpy99I, PDBid 3FC3_A), their non-catalytic homologs (MH1 domain of transcription factor Smad, PDBid 1MHD_A) [19, 98] (Pfam clan CL0263) (SCOPid 54059), bacteriophage P1 encoded-Ref protein (PDBid 3PLW_A), DNA fragmentation factor 40 KDa subunit (DFF40, also called Caspase-activated DNase, CAD, PDBid 1V0D_A) and prophage-derived uncharacterised protein ybcO (PDBid 3G27_A) (**Supplementary Figure viii**). Detection of TCs in some members of this family is hindered

because of the loss of zinc-chelating residues and degradation of the core SSEs including the zinc knuckle and α-helix [19]. The primary β-hairpin is the most conserved secondary structure in the TC fold of this family and harbours the catalytic site with an invariant histidine (except for PacI, PDBid 3LDY_A, where a nearby tyrosine is proposed to a play an equivalent role) [99, 100]. Variable sized insertions are present in between the two β-strands of the primary β-hairpin.

The bacteriophage P1-encoded Ref protein (PDBid 3PLW_A) belongs to the HNH-family of nucleases based on structure, function and motif conservation [101]. A sequence based search initiated with the TC of Ref protein (PDBid 3PLW_A) finds marginal similarity to bonafide His-Me proteins (for example, FFAS search finds match to Colicin E7, PDBid 3GJN_B, Score= -8.89). Further, FFAS search with Ref (PDBid 3PLW_A) finds match to prophage-derived uncharacterised protein ybcO (PDBid 3G27_A, Score= -10.2), a member of DUF1364 (PF07102). YbcO has a histidine on the primary β-hairpin of TC that superimposes on the catalytic histidine of His-Me nucleases. We predict that this protein is likely to function as a nuclease and thus, classify it in this family.

DFF40 (PDBid 1V0D_A) has a His-Me TC, but the chelation pattern of the structural zinc is different from other TCs. Of the four metal-chelating residues, three are contributed by the zinc knuckle and only one is contributed from the α-helix [102].

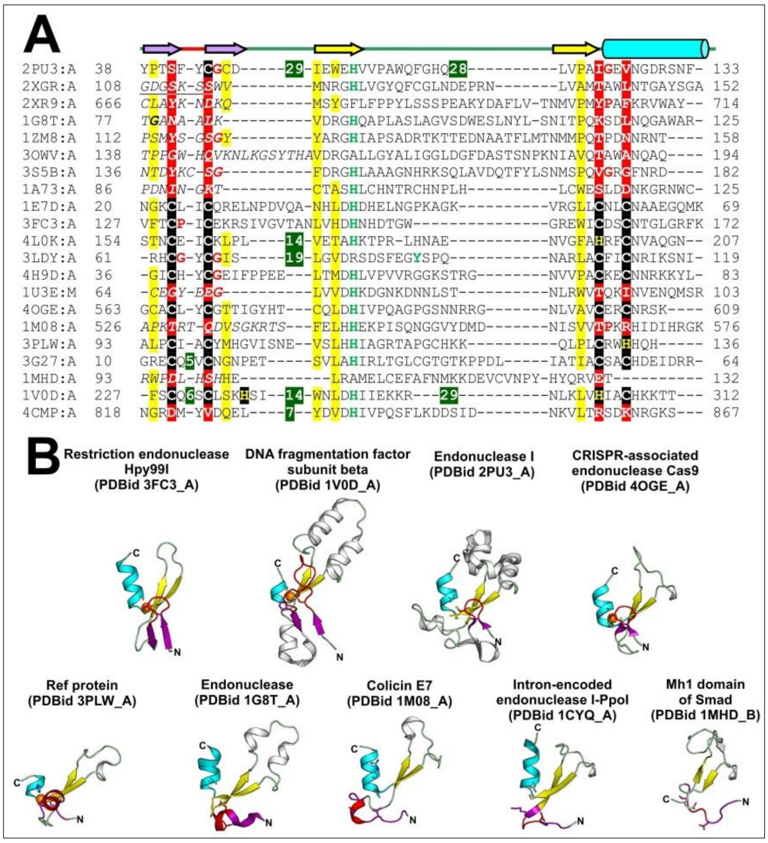

Supplementary Figure viii: His-Me family

(A) Structure-based MSA of His-Me family. The sequences used for alignment were obtained after clustering all sequences of the family in the dataset at 40 %ID. The catalytic residue is coloured green. Sequence regions that are disordered in structure are underlined and italicized. **(B)** Ribbon diagrams of His-Me family representatives.

13. Treble clef domain of thymidine kinase

Thymidine kinase has a C-terminal lasso domain which binds a structural zinc ion (PDBid 2J9R_A) [103]. This domain structurally resembles a TC ZF and is named so because of the presence of a substrate (thymidine)-binding flexible region in between the β-strands of the primary β-hairpin, wherein main chain backbone atoms interact with the nitrogenous base [103, 104] (**Supplementary Figure ix**).

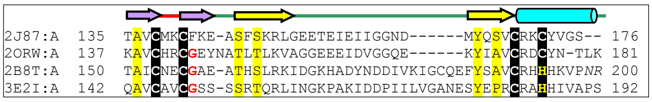

Supplementary Figure ix: Structure-based MSA of thymidine kinase family.
The sequences used for alignment were obtained after clustering all sequences of the family in the dataset at 40 %ID

14. YlxR-like hypothetical cytosolic protein

YlxR protein from *Streptococcus pneumonia* (PDBid 1G2R_A) has been previously shown to possess a TC fold that lacks metal-chelating residues [3]. Homologous proteins possess none to all four zinc binding aminoacids (PF04296). The TC fold of this protein has an additional β-strand inserted in between the two β-strands of the primary β-hairpin that together form a three-stranded β-sheet. This TC has two additional C-terminal α-helices and the resulting fold has a two-layer α/β sandwich structure that is classified as a separate fold in SCOP (SCOPid 64375).

15. tRNA synthetase treble clef domain

Prolyl-tRNA synthetases have a C-terminal domain with a zinc-binding site in many structurally characterised homologs (PDBids 1H4Q_A, 1NJ1_A) [105, 106]. The zinc binding region has been described previously as a circularly permuted TC [3] (**Supplementary Figure x**). The zinc-chelating aminoacids are selectively conserved across the archaeal, bacterial and eukaryotic lineages, and a conserved tyrosine has been proposed to stabilize the basic residues that interact with ATP pyrophosphate [105]. This domain is also suggested to function in positioning the acceptor tRNA arm properly in the active site [107].

16. Zinc-binding site in N-terminal domain of CasA/Cse1 subunit of CRISPR

The zinc binding site in the N-terminal domain of CasA/Cse1 subunit of CRISPR Cascade complex [108] resembles a circularly permuted TC motif (PDBid 4TVX_I, **Supplementary Figure x**). This zinc site is not conserved in all homologs, for example, CasA from *Thermus thermophilus* HB8 (PDBid 4F3E_A) and *Acidimicrobium ferrooxidans* DSM 10331 (PDBid 4H3T_A) lack the zinc-binding residues and the zinc knuckle region of the TC. The TC-like region possesses a loop that likely mediates interaction with a region of CRISPR-derived RNAs and target DNA [108, 109].

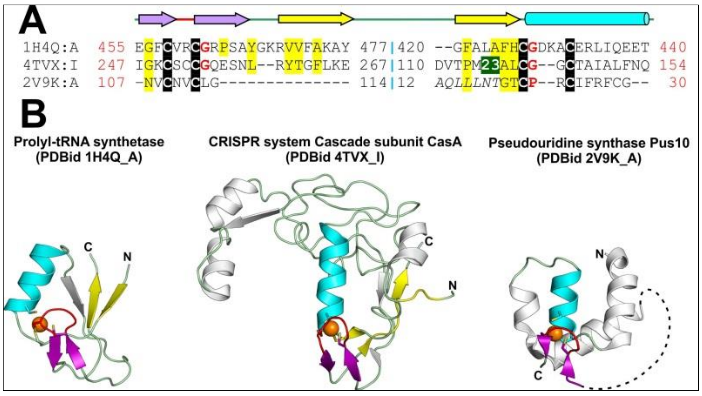

Supplementary Figure x: Circularly permuted treble clefs

(A) Structure-based MSA of representatives of treble clefs from tRNA synthetase, CasA and Pus10. **(B)** Ribbon diagrams of representatives of tRNA synthetase, CasA and Pus10.

17. Zinc-binding site in N-terminal domain of pseudouridine synthase Pus10

Pus10 (PDBid 2V9K_A, **Supplementary Figure x**) has a structural zinc ion in its N-terminal domain. A pair of zinc-binding ligands is contributed a knuckle and another pair from the beginning of a α-helix, as seen in TCs. However, like the prolyl-tRNA synthetase and CRISPR CasA, these SSEs are permuted in topology when compared to the classical TC. Binding of zinc ion maintains the structural integrity of the N-terminal domain and Arg27 on the α-helix of the TC is part of the proposed RNA-binding basic cleft [110].

18. NAD$^+$-dependent DNA ligase treble clef domain

TC of NAD$^+$-dependent DNA ligase is one of the shortest and the α-helix is replaced by a short helical region that is continuous with the α-helix of the proceeding HhH domain (PDBid 1DGS_A) [3]. The primary β-hairpin of this TC is involved in DNA binding [111].

19. YggX-like hypothetical protein

A conserved Gram-negative bacterial protein YggX (PDBid 1T07_A) has a TC fold, but there is no metal ion bound in the structure and the primary β-hairpin is replaced by a loop and a α-helix. Sequence analysis reveals the presence of four zinc-chelating aminoacids in several homologs (**Supplementary Figure xi**). This protein is implicated in bacterial oxidative stress response [112-114].

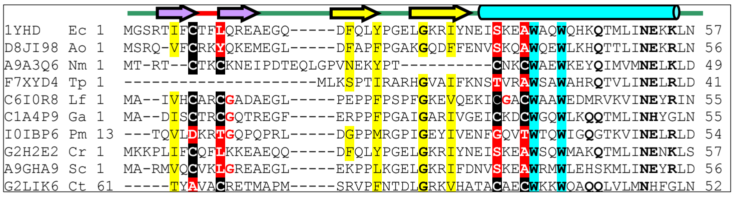

Supplementary Figure xi: Structure-based MSA of YggX-like family proteins.

The sequences used for alignment were obtained after clustering all sequences in the Pfam family (PF04362) at 40 %ID. The PDBid/UniProtID, organism name abbreviation and start and end residue number are mentioned for each sequence. Organism name abbreviations: Ec- *Escherichia coli*, Ao- *Acinetobacter oleivorans* (strain JCM 16667 / KCTC 23045 / DR1), Nm- *Nitrosopumilus maritimus* (strain SCM1), Tp- *Tremblaya princeps* (strain PCIT), Lf- *Leptospirillum ferrodiazotrophum*, *Ga- Gemmatimonas aurantiaca* (strain T-27 / DSM 14586 / JCM 11422 / NBRC 100505), Pm- *Phycisphaera mikurensis* (strain NBRC 102666 / KCTC 22515 / FYK2301M01), Cr- *Candidatus Regiella insecticola* R5.15, Sc- *Sorangium cellulosum* (strain So ce56), Ct- *Chloracidobacterium thermophilum* (strain B).

20. RPB10 protein from RNA polymerase

RPB10 folds into a three-helical bundle (SCOPid 46924) [3, 115]. A zinc-binding site constituted by metal-chelating aminoacids contributed from the beginning of the third α-helix and from a zinc knuckle-like extension of the helical bundle at the N-terminal (PDBid 1EF4_A) is seen in the protein. The zinc site resembles a TC fold, although the primary β-hairpin is substituted by two α-helices that form the core of the helical bundle fold.

21. C-terminal zinc finger in E3 ligase HOIP

The C-terminal region of HOIP (PDBid 4LJO_A), also called LDD (Linear ubiquitin chain Determining Domain; residues 910-1072) [116, 117], has a TC fold (residues 967-1014) whose primary β-hairpin is replaced by a loop and a β-hairpin is inserted in between the two zinc-chelating residues of the zinc knuckle. This inserted β-hairpin has been suggested to interact with the donor ubiquitin [116].

22. Treble clef domain in ClpX

Chaperone ClpX has a TC at its N-terminal that helps in dimerization and substrate binding [118]. The first β-strand of the primary β-hairpin forms antiparallel β-sheet interactions with the substrate (PDBid 2DS8) [118]. Equivalent hydrophobic residues from the knuckle, primary β-hairpin and the α-helix from both the monomers form the dimeric interface [119].

23. Zinc finger domain in ATP sulfurylase

Archaeal ATP sulfurylase has a zinc-binding site in the third domain of the enzyme (PDBid 1V47) [120], that is absent in eukaryotic and bacterial homologs (PDBids 1X6V, 2GKS). This zinc-binding site is suggested to provide thermal stability and also help in homodimerization [120]. The TC lacks the primary β-hairpin and the orientation of the knuckle-β-hairpin with respect to the α-helix is unlike bonafide TCs.

24. RecR-like treble clef

This family has the TC domain from recombination protein RecR (PDBid 1VDD_A) and the C-terminal TC of tRNA thiouridine synthetase TtuA (PDBid 3VRH_A). RecR-like TC is ~20 aminoacids in length and the primary β-hairpin is reduced to a short turn. HHpred search initiated with the TC of TtuA (PDBid 3VRH) finds RecR TC (PDBid 3VDP) with an E-value=0.075 and these could be superimposed with an RMSD=0.45 Å over 20 pairs of backbone C_α atoms. The ZFD of RecR is proposed to help in interacting with DNA and also provide structural stability [121]. The TC domains of TtuA along with the catalytic domain are implicated in tRNA binding [122].

25. Neural zinc-finger

This is a family of DNA-interacting TCs (PDBids 2MF8, 2CS8), most of which lack well-structured SSEs [123, 124] and thus, could be confused with Gag knuckles or zinc-binding loops. However, in ST18 (PDBid 2CS8) the second zinc binding half-site is seen to be contributed from the N-terminal base of a structured-α-helix that argues for their classification as TCs.

26. E6 C-terminal domain-like

Human papillomavirus (HPV) E6 oncoprotein has tandemly-duplicated domains (PDBid 4GIZ), each with one zinc-binding site. The topological arrangement of zinc-chelating SSEs resembles a TC-like fold with extensions and insertions to the core. The two ZFDs of E6 are positioned in suitable conformation to form a binding pocket for acidic LxxLL motifs of interacting proteins (PDBids 4GIZ, 3PY7) [125]. The N-terminal TC domain in certain strains of HPV is additionally implicated in homodimerization [126] and the C-terminal domain is suggested to interact with DNA via its positively-charged surface [127].

27. Bacteriophage λ Ea8.5-like treble clef

Structures of bacteriophage λ Ea8.5 homologs (PDBids 2M7A, 2M7B) reveal a fusion of homeodomain and TC [128]. Their structure comprises of αββααα SSEs, where α-1, 2 and 3 constitute the homeodomain, and two β-strands form the knuckle-containing β-hairpin and α-4 contributes the second zinc-chelating half-site of the TC. This TC lacks the primary β-hairpin and the region in between the two zinc binding half-sites is occupied by the α-2 and α-3 of the homeodomain. λ Ea8.5 is shown to be indispensable for λ-mediated inhibition of DNA-synthesis-initiation in *Escherichia coli* and a protein-protein interaction role is assigned to this protein [128, 129].

28. Mononuclear treble clef domain of UPF1 and replicase polyprotein 1ab

This family groups the mononuclear TC domain of UPF1 (PDBid 2WJV_A) and replicase polyprotein 1ab (PDBid 4N0N_A) that lies in between the respective binuclear RING-like and helicase domains in both these proteins. The mononuclear TC from these two proteins has been provisionally grouped together based on similar co-occurring domains that themselves share a homologous relationship. The TC domain of replicase polyprotein 1ab (PDBid 4N0N) is very short and lacks defined SSEs, and the first two zinc-binding residues are separated by only a single aminoacid. β-hairpin of the TC domain of UPF1 (PDBid 2WJV) forms an extensive hydrophobic network of interactions with the UPF2 [31].

29. A20-like zinc fingers

A20-like ZF (PDBid 3OJ4_C/F) is a ubiquitin-interacting TC where the primary β-hairpin is replaced by a loop. Regions of this loop are involved in ubiquitin binding and the interactions are hydrophobic and polar in nature [32].

30. Dimerization domain in the Fur family

The dimerization domain of Fur family transcription factors (PDBids 2FE3, 2O03, 1MZB, **Supplementary Figure xii**) consists of an α/β fold with a structural zinc-binding site [130]. The zinc is bound by residues contributed by a knuckle and a α-helix, like in a bonafide TC, but the primary β-hairpin is substituted by a α-helix and a β-strand. The latter forms an antiparallel β-sheet with the zinc knuckle β-hairpin and mediates dimerization by interacting with an equivalent β-strand from the other monomer. Dimerization, thus, leads to the formation of a contiguous six-stranded antiparallel β-sheet.

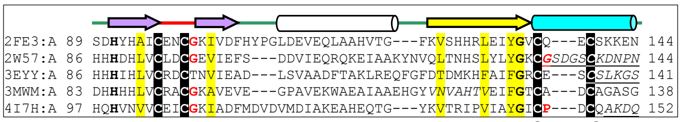

Supplementary Figure xii: Structure-based MSA of Fur family.
Sequences used for alignment were obtained after clustering all sequences of the family in the dataset at 40 %ID. Sequence regions which are disordered in structure are underlined and italicized.

31. GATA-like knotted treble clefs of pre-mRNA-splicing factor Rds3p

Rds3p is an essential subunit of the SF3b complex of U2 snRNP [131]. Rds3p structure (PDBid 2K0A_A) resembles a "triquetra" symbol and is a unique example of knotted ZFDs with three GATA-like TC ZFDs, one at each vertex of the roughly triangular structure [132]. Two of the three ZFDs are circularly permuted with respect to the bonafide structure of the GATA-like TC in the third ZFD (**Supplementary Figure xiii**). This triquetra motif is suggested to have likely arisen by a tandem series of events, including duplication, deletion, and segment-swapping [132].

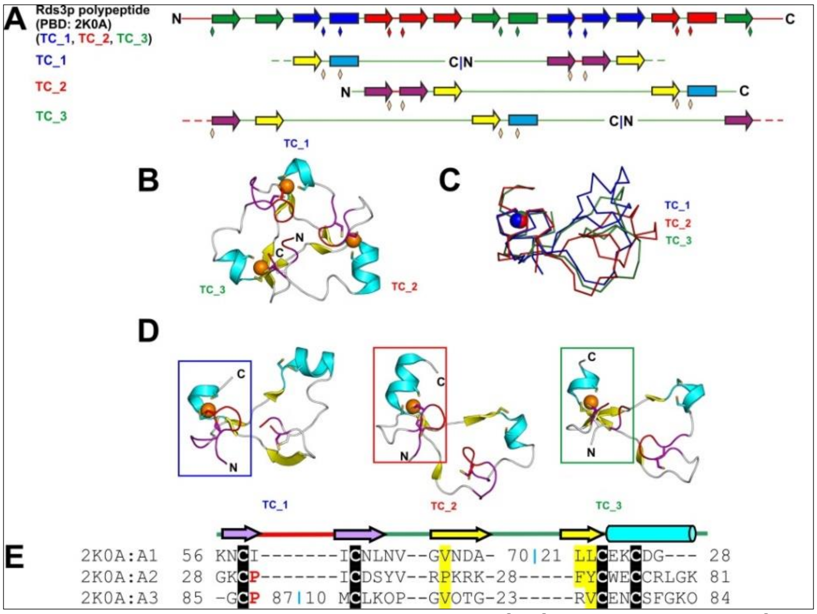

Supplementary Figure xiii: GATA-like knotted treble clefs of pre-mRNA-splicing factor Rds3p.
(A) Topology diagram of the complete and individual treble clefs of the Rds3p protein. **(B)** Tertiary structure of Rds3p (PDBid 2K0A). **(C)** Stereo diagram of superimposition of the individual treble clef motifs. **(D)** Ribbon representation of individual treble clef motifs. **(E)** Structure-based MSA of the individual treble clefs of Rds3p.

32. SAP30-like treble clef zinc finger

SAP30 (PDBid 2KDP_A), a subunit of the histone deacetylase-associated mammalian Sin3 corepressor complex, has a TC domain that is suggested to interact with dsDNA [133]. The primary β-hairpin of the TC is substituted by a α-helix and a β-strand. The latter forms β-sheet interactions with a β-strand inserted between the first two zinc-chelating residues from the zinc knuckle. Nucleic acid interacting region is suggested to be constituted of the positively-charged residues on the α-helical insertion and the α-helix of the TC, and the adjacent loop regions [133]. Dali search initiated with SAP30 TC (PDBid 2KDP_A) finds transcription factor grauzone (PDBid 1PZW_A, Z-score=3.6, RMSD=3.2 Å, nali=58) as the only match.

33. Zinc finger associated domain of grauzone Cg33133-Pa

Transcription regulator grauzone has a TC ZF (PDBid 1PZW_A) at its N-terminal that has a very long α-helix, rich in hydrophobic aminoacids [134]. This α-helix and a α-helix inserted in between the β-strands of the primary β-hairpin are suggested to mediate homodimerization [134].

34. THAP domain

The Thanatos-associated protein (THAP) domain is a DNA-binding TC restricted to animals and is structurally characterised from proteins such as P-element transposase (PDBid 3KDE_C), the transcription repressor CtBP (PDBid 2JM3_A), and cell proliferation regulator protein TAHP1 (PDBid 2JTG_A) [135-137]. THAP domain has a α-helical insertion in between the β-strands of the primary β-hairpin and shares full-length structural similarity with grauzone (PDBid 1PZW_A) as suggested previously [136] (manual superimposition with RMSD=1.97 Å over 28 pairs of backbone C_α atoms). The primary β-hairpin in THAP mediates interactions with dsDNA by docking into its major groove while a C-terminal loop binds an adjacent minor groove (PDBid 3KDE) [135].

35. Treble clef domain in the FusB-like proteins

A structure-stabilizing TC is present at the C-terminal of FusB (PDBid 4ADN) and FusC (PDBid 2YB5). The SSEs of this TC are not oriented in a canonical manner, with an additional N-terminal β-strand forming an antiparallel β-sheet with the primary β-hairpin. The third zinc-binding residue is contributed from the end of primary β-hairpin and not from the α-helix and is separated by five residues from the last zinc-chelating residues [138]. The primary β-hairpin is suggested to mediate interactions with elongation factor G [139].

36. Treble clef domain of Cmr2

Cmr2 (PDBids 3UNG, 4DOZ), a Cas10 protein of the CRISPR-Cas RNA silencing complex has a TC ZFD, similar to ribosomal protein S14, in between the first adenylyl cyclase-like and α-helical domain [140, 141].

37. Zinc binding domain from MCM10

The internal domain of MCM10 protein (PDBid 3EBE_A) has a TC ZFD [142, 143] that lacks the primary β-hairpin, and the zinc-chelating residues on the knuckle are separated by nine residues. Residues from the loop joining the knuckle-β-hairpin with the α-helix are proposed to mediate DNA interactions along with the OB domain that precedes the ZFD [142, 143].

38. Protein lsr2

N-terminal domain of lsr2, a putative functional analog of H-NS nucleoid-associated proteins, folds into a β-β-α motif (PDBid 4E1P) [144, 145]. By including a zinc knuckle preceding the β-β-α as a part of the structural fold, we annotate this domain to possess as a typical TC fold. The structure of lsr2 from *Mycobacterium tuberculosis* does not chelate a zinc ion but we observe that some lsr2 homologs do preserve all the four putative metal-chelating residues (UniProtIDs I8QSD7, Q47NC6). Dali search initiated with lsr2p (PDBid 4E1P_A) retrieves a number of TCs, such as 50S ribosomal protein L28 (PDBid 2Y0X_1, Z-score=4.1, RMSD=4.6 Å, nali=49) and thymidine kinase (PDBid 2ORW_B, Z-score=3.7, RMSD=4.2 Å, nali=41). The primary β-hairpin of the domain is seen involved in homodimerization in the structure and suggested to form linear chains of N-lsr2 on the proteolytic removal of three N-terminal residues [145], suggestive of a likely mechanism for lsr2's function as DNA-bridging and chromatin compaction protein [145-147].

Lsr2 protein is conserved in mycobacteria, some related actinobacteria [148] and the viruses that infect them (PF11774) [149]. Sequence similarity searches initiated with the full-length lsr2 (UniProtID P9WIP7) do not retrieve significant matches to proteins outside this family. However, we do observe similar domain organization in lsr2p and T4 Endonuclease VII (PDBid 1E4L_A), i.e. a TC followed by a α-helical bundle. Indeed, the C-terminal α-helical domain in both these protein families is placed under the same homology and topology group, *viz.* LEM/SAP HeH motif, in Evolutionary Classification of protein Domains (ECOD) [150], indicating a plausible remote evolutionary relationship in these domains. We also observe that the TC helps in dimerization in both these proteins, although the SSEs forming the dimerization interface are not identical.

39. Treble clef zinc finger domain of zinc finger protein 483

The TC of zinc finger protein 483 (PDBid 2CTU) possesses a loop in place of the primary β-hairpin and lacks significant sequence similarity with other TCs. Dali search initiated with this TC retrieves bonafide TC domains such as UPF0269 protein yggX (PDBid 1YHD_A, Z-score=2.4 Å, RMSD=5.1, nali=49) and hypothetical UPF0269 protein PA5148 (PDBid 1YHD_A, Z-score=2.3, RMSD=5.6 Å, nali=50).

40. Treble clef like zinc finger in hypothetical protein ORF126

The structure of hypothetical protein ORF126 from *Pyrobaculum spherical virus* (PDBid 2X5R), a structural genomics target, has a zinc-binding site within a novel fold. The zinc-binding site resembles a TC motif but the α-helix is reduced to a single turn. The primary β-hairpin of the TC forms a β-sheet with the other β-strands in the complete protein. Dali search initiated with only the TC-like region of ORF126 retrieves TC domain of 40S ribosomal protein S21 (PDBid 4BPE_Z, Z-score=2.5, RMSD=2.4 Å, nali=34).

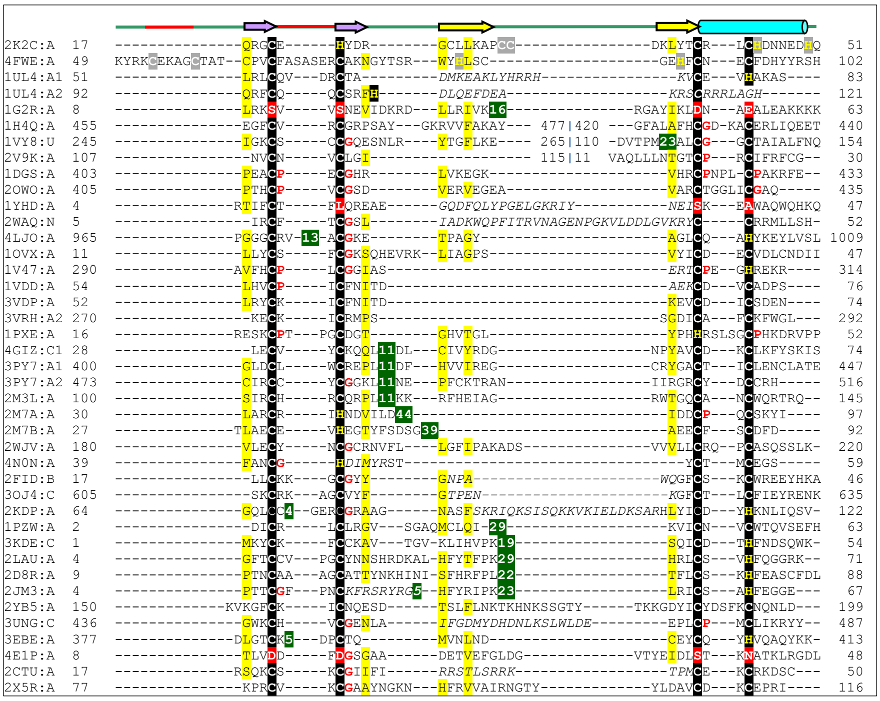

Supplementary Figure xiv: Structure-based MSA of some treble clef fold group proteins. This MSA has treble clefs from families with only one to few members and are thus not included in MSAs of other families. The sequences for this alignment were obtained by clustering all the sequences in the dataset at 40 %ID.

de novo proteins

This not a true family and members of this group (PDBids 1UW1_A, 3DGL_A, 2LZE) have no evolutionary relationship with bonafide TCs. This group has structures of *in vitro* designed sequences that analogously fold into TC-like ZFs [151]. Some of these proteins have been evolved *in vitro* to bind ATP (PDBid 1UW1_A) [152-154] and few others for the study of enzyme emergence and evolution (PDBids 3DGL, 2LZE) [155, 156].

INTERACTIONS OF TC DOMAINS WITH OTHER BIOMOLECULES

TCs interact with a variety of biomolecules including nucleic acids, proteins, small molecules such as lipids and nucleosides (**Supplementary Figure xv, xvi, xvii**). TCs also mediate dimerization in a number of proteins and a small fraction of TCs also function as enzymes (**Supplementary Figure xvii**).

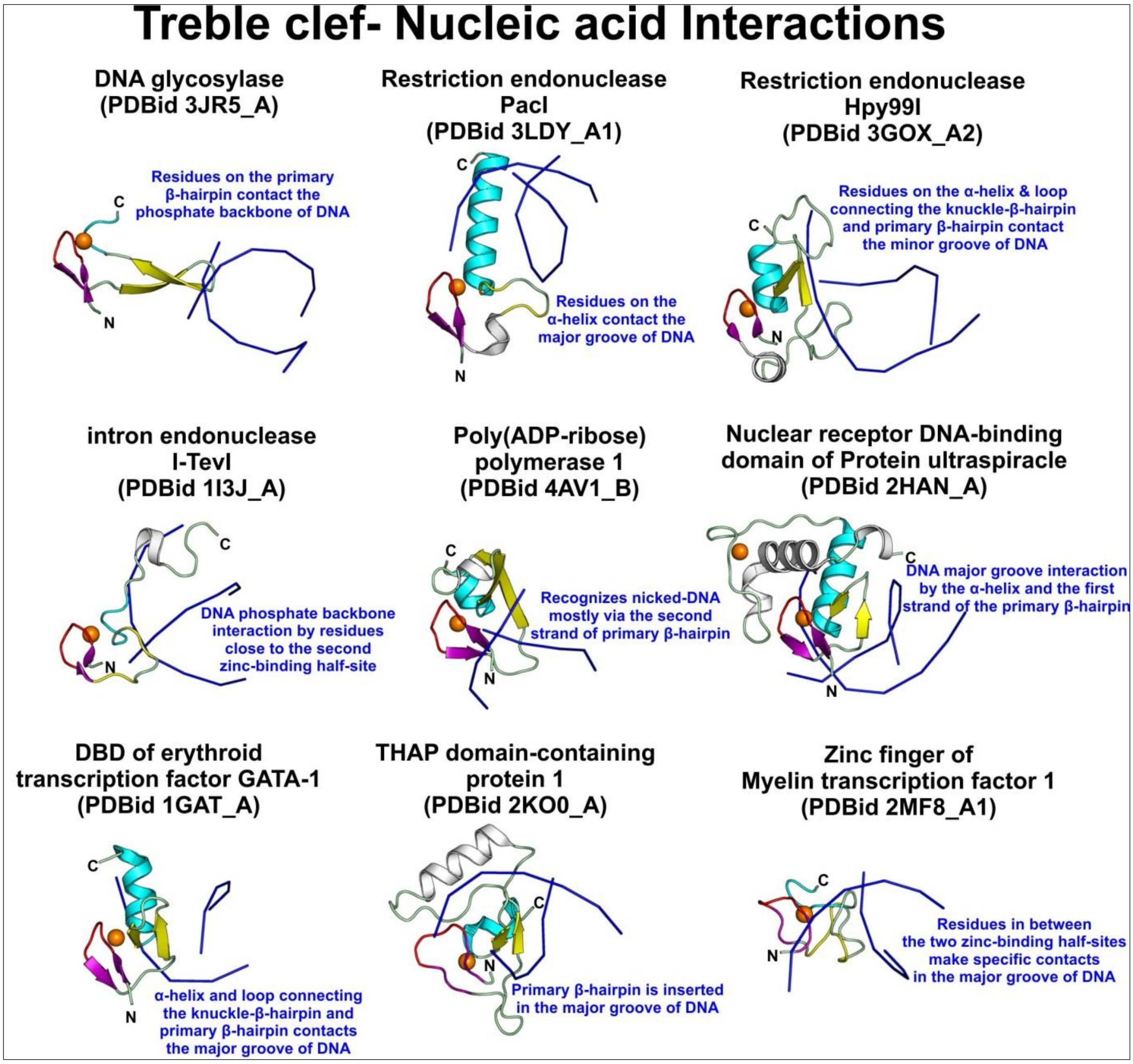

Supplementary Figure xv: Treble clef- Nucleic acid interactions

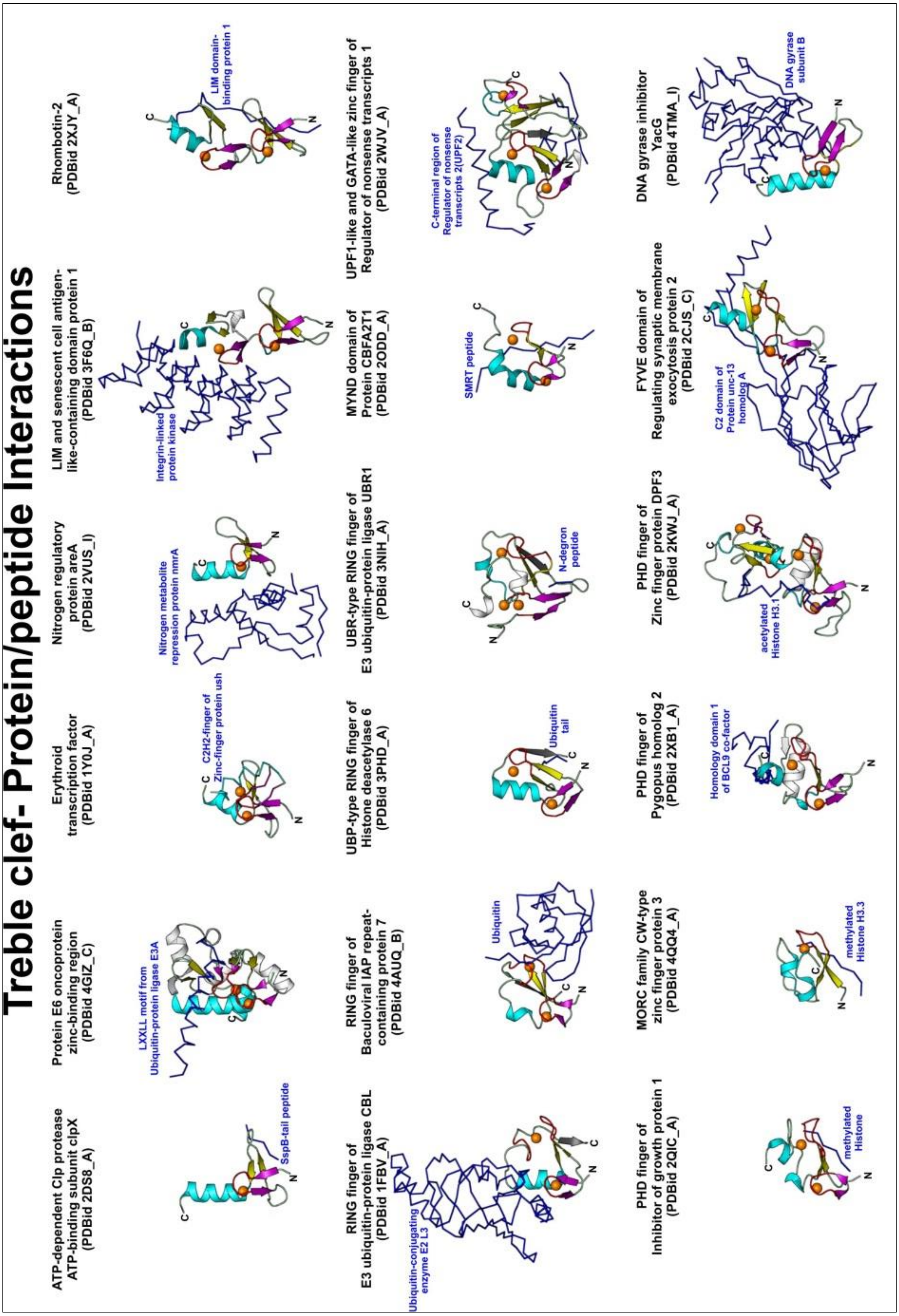

Supplementary Figure xvi: Treble clef- Protein/peptide interactions

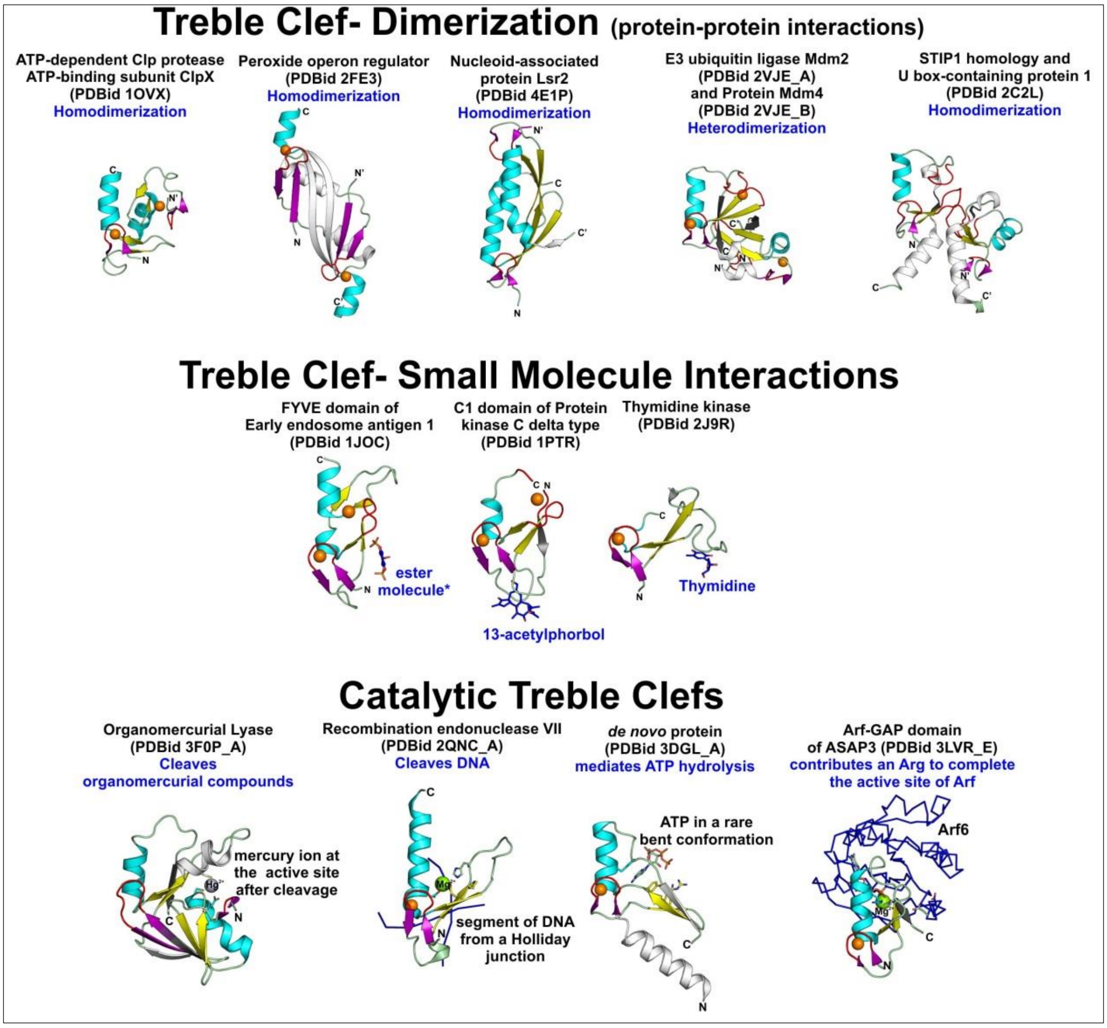

Supplementary Figure xvii: Treble clef domains involved in dimerization, small molecule interactions and mediating enzymatic reactions. (‘*’ indicates Inositol 1,3-bisphosphate)

References

1. Ettema, T.J., Huynen, M.A., de Vos, W.M. & van der Oost, J. TRASH: a novel metal-binding domain predicted to be involved in heavy-metal sensing, trafficking and resistance. *Trends in biochemical sciences* **28**, 170-173 (2003).
2. Punta, M. et al. The Pfam protein families database. *Nucleic acids research* **40**, 29 (2012).
3. Krishna, S.S., Majumdar, I. & Grishin, N.V. Structural classification of zinc fingers: survey and summary. *Nucleic acids research* **31**, 532-550 (2003).
4. Kaur, G. & Subramanian, S. Repurposing TRASH: Emergence of the enzyme organomercurial lyase from a non-catalytic zinc finger scaffold. *Journal of structural biology* **188**, 16-21 (2014).
5. Monleon, D., Yee, A., Arrowsmith, C. & Celda, B. NMR structure of hypothetical protein TA0938 from *Thermoplasma acidophilum*. *Proteins* **67**, 1185-1188 (2007).
6. Bakolitsa, C. et al. The structure of Jann_2411 (DUF1470) from *Jannaschia sp*. at 1.45 A resolution reveals a new fold (the ABATE domain) and suggests its possible role as a transcription regulator. *Acta crystallographica. Section F, Structural biology and crystallization communications* **66**, 1198-1204 (2010).

7. Schelert, J., Rudrappa, D., Johnson, T. & Blum, P. Role of MerH in mercury resistance in the archaeon *Sulfolobus solfataricus*. *Microbiology* **159**, 1198-1208 (2013).
8. Lechtenberg, B.C., Allen, M.D., Rutherford, T.J., Freund, S.M. & Bycroft, M. Solution structure of the FCS zinc finger domain of the human polycomb group protein L(3)mbt-like 2. *Protein science : a publication of the Protein Society* **18**, 657-661 (2009).
9. Jamshher, M. & Laxmi, A. DUF581 is plant specific FCS-like zinc finger involved in protein-protein interaction. *PloS one* **9**, e99074 (2014).
10. Klein, D.J., Moore, P.B. & Steitz, T.A. The roles of ribosomal proteins in the structure assembly, and evolution of the large ribosomal subunit. *Journal of molecular biology* **340**, 141-177 (2004).
11. He, F. et al. Solution structure of the zinc finger HIT domain in protein FON. *Protein science : a publication of the Protein Society* **16**, 1577-1587 (2007).
12. Matthews, J.M. et al. It takes two to tango: the structure and function of LIM, RING, PHD and MYND domains. *Current pharmaceutical design* **15**, 3681-3696 (2009).
13. Taubner, L.M., McGuirl, M.A., Dooley, D.M. & Copie, V. Structural studies of Apo NosL, an accessory protein of the nitrous oxide reductase system: insights from structural homology with MerB, a mercury resistance protein. *Biochemistry* **45**, 12240-12252 (2006).
14. Di Lello, P. et al. NMR structural studies reveal a novel protein fold for MerB, the organomercurial lyase involved in the bacterial mercury resistance system. *Biochemistry* **43**, 8322-8332 (2004).
15. Freemont, P.S., Hanson, I.M. & Trowsdale, J. A novel cysteine-rich sequence motif. *Cell* **64**, 483-484 (1991).
16. Barlow, P.N., Luisi, B., Milner, A., Elliott, M. & Everett, R. Structure of the C3HC4 domain by 1H-nuclear magnetic resonance spectroscopy. A new structural class of zinc-finger. *Journal of molecular biology* **237**, 201-211 (1994).
17. Deshaies, R.J. & Joazeiro, C.A. RING domain E3 ubiquitin ligases. *Annual review of biochemistry* **78**, 399-434 (2009).
18. Burroughs, A.M., Iyer, L.M. & Aravind, L. Functional diversification of the RING finger and other binuclear treble clef domains in prokaryotes and the early evolution of the ubiquitin system. *Molecular bioSystems* **7**, 2261-2277 (2011).
19. Grishin, N.V. Treble clef finger--a functionally diverse zinc-binding structural motif. *Nucleic acids research* **29**, 1703-1714 (2001).
20. Mylona, A. et al. Structure of the tau60/Delta tau91 subcomplex of yeast transcription factor IIIC: insights into preinitiation complex assembly. *Molecular cell* **24**, 221-232 (2006).
21. Kaur, G. & Subramanian, S. A novel RING finger in the C-terminal domain of the coatomer protein alpha-COP. *Biology direct* **10**, 70 (2015).
22. Aravind, L. & Koonin, E.V. The U box is a modified RING finger - a common domain in ubiquitination. *Current biology : CB* **10**, R132-134 (2000).
23. Kaur, G. & Subramanian, S. The UBR-box and its relationship to binuclear RING-like treble clef zinc fingers. *Biology direct* **10**, 36 (2015).
24. Aravind, L., Iyer, L.M. & Koonin, E.V. Comparative genomics and structural biology of the molecular innovations of eukaryotes. *Current opinion in structural biology* **16**, 409-419 (2006).
25. Borden, K.L. RING domains: master builders of molecular scaffolds? *Journal of molecular biology* **295**, 1103-1112 (2000).
26. Duan, X. et al. Structural and functional insights into the roles of the Mms21 subunit of the Smc5/6 complex. *Molecular cell* **35**, 657-668 (2009).
27. Linke, K. et al. Structure of the MDM2/MDMX RING domain heterodimer reveals dimerization is required for their ubiquitylation in trans. *Cell death and differentiation* **15**, 841-848 (2008).
28. Mattiroli, F. et al. RNF168 ubiquitinates K13-15 on H2A/H2AX to drive DNA damage signaling. *Cell* **150**, 1182-1195 (2012).
29. Tu, D., Li, W., Ye, Y. & Brunger, A.T. Structure and function of the yeast U-box-containing ubiquitin ligase Ufd2p. *Proceedings of the National Academy of Sciences of the United States of America* **104**, 15599-15606 (2007).

30. Taylor, N.G. Cellulose biosynthesis and deposition in higher plants. *The New phytologist* **178**, 239-252 (2008).
31. Clerici, M. et al. Unusual bipartite mode of interaction between the nonsense-mediated decay factors, UPF1 and UPF2. *The EMBO journal* **28**, 2293-2306 (2009).
32. Hurley, J.H., Lee, S. & Prag, G. Ubiquitin-binding domains. *The Biochemical journal* **399**, 361-372 (2006).
33. Hadjivassiliou, H., Rosenberg, O.S. & Guthrie, C. The crystal structure of *S. cerevisiae* Sad1, a catalytically inactive deubiquitinase that is broadly required for pre-mRNA splicing. *RNA* **20**, 656-669 (2014).
34. Choi, W.S. et al. Structural basis for the recognition of N-end rule substrates by the UBR box of ubiquitin ligases. *Nature structural & molecular biology* **17**, 1175-1181 (2010).
35. Delvecchio, M., Gaucher, J., Aguilar-Gurrieri, C., Ortega, E. & Panne, D. Structure of the p300 catalytic core and implications for chromatin targeting and HAT regulation. *Nature structural & molecular biology* **20**, 1040-1046 (2013).
36. Sengoku, T. & Yokoyama, S. Structural basis for histone H3 Lys 27 demethylation by UTX/KDM6A. *Genes & development* **25**, 2266-2277 (2011).
37. Leiros, I., Timmins, J., Hall, D.R. & McSweeney, S. Crystal structure and DNA-binding analysis of RecO from *Deinococcus radiodurans*. *The EMBO journal* **24**, 906-918 (2005).
38. Mandiyan, V., Andreev, J., Schlessinger, J. & Hubbard, S.R. Crystal structure of the ARF-GAP domain and ankyrin repeats of PYK2-associated protein beta. *The EMBO journal* **18**, 6890-6898 (1999).
39. Ismail, S.A., Vetter, I.R., Sot, B. & Wittinghofer, A. The structure of an Arf-ArfGAP complex reveals a Ca2+ regulatory mechanism. *Cell* **141**, 812-821 (2010).
40. Massiah, M.A. et al. Solution structure of the MID1 B-box2 CHC(D/C)C(2)H(2) zinc-binding domain: insights into an evolutionarily conserved RING fold. *Journal of molecular biology* **369**, 1-10 (2007).
41. Reymond, A. et al. The tripartite motif family identifies cell compartments. *The EMBO journal* **20**, 2140-2151 (2001).
42. Komander, D. et al. The structure of the CYLD USP domain explains its specificity for Lys63-linked polyubiquitin and reveals a B box module. *Molecular cell* **29**, 451-464 (2008).
43. Short, K.M., Hopwood, B., Yi, Z. & Cox, T.C. MID1 and MID2 homo- and heterodimerise to tether the rapamycin-sensitive PP2A regulatory subunit, alpha 4, to microtubules: implications for the clinical variability of X-linked Opitz GBBB syndrome and other developmental disorders. *BMC cell biology* **3**, 1 (2002).
44. Afroz, T. et al. A fly trap mechanism provides sequence-specific RNA recognition by CPEB proteins. *Genes & development* **28**, 1498-1514 (2014).
45. Merkel, D.J. et al. The C-terminal region of cytoplasmic polyadenylation element binding protein is a ZZ domain with potential for protein-protein interactions. *Journal of molecular biology* **425**, 2015-2026 (2013).
46. Nishizuka, Y. The molecular heterogeneity of protein kinase C and its implications for cellular regulation. *Nature* **334**, 661-665 (1988).
47. Colon-Gonzalez, F. & Kazanietz, M.G. C1 domains exposed: from diacylglycerol binding to protein-protein interactions. *Biochimica et biophysica acta* **1761**, 827-837 (2006).
48. Zhang, G., Kazanietz, M.G., Blumberg, P.M. & Hurley, J.H. Crystal structure of the cys2 activator-binding domain of protein kinase C delta in complex with phorbol ester. *Cell* **81**, 917-924 (1995).
49. Heo, J., Thapar, R. & Campbell, S.L. Recognition and activation of Rho GTPases by Vav1 and Vav2 guanine nucleotide exchange factors. *Biochemistry* **44**, 6573-6585 (2005).
50. Kellenberger, E. et al. Solution structure of the C-terminal domain of TFIIH P44 subunit reveals a novel type of C4C4 ring domain involved in protein-protein interactions. *The Journal of biological chemistry* **280**, 20785-20792 (2005).
51. Zhang, Q. et al. Structure-function analysis reveals a novel mechanism for regulation of histone demethylase LSD2/AOF1/KDM1b. *Cell research* **23**, 225-241 (2013).
52. Fang, R. et al. LSD2/KDM1B and its cofactor NPAC/GLYR1 endow a structural and molecular model for regulation of H3K4 demethylation. *Molecular cell* **49**, 558-570 (2013).

53. Sheng, Y. et al. Molecular basis of Pirh2-mediated p53 ubiquitylation. *Nature structural & molecular biology* **15**, 1334-1342 (2008).
54. Huang, J. et al. ZNF216 Is an A20-like and IkappaB kinase gamma-interacting inhibitor of NFkappaB activation. *The Journal of biological chemistry* **279**, 16847-16853 (2004).
55. Myers, L.C., Verdine, G.L. & Wagner, G. Solution structure of the DNA methyl phosphotriester repair domain of *Escherichia coli* Ada. *Biochemistry* **32**, 14089-14094 (1993).
56. He, C. et al. A methylation-dependent electrostatic switch controls DNA repair and transcriptional activation by *E. coli* ada. *Molecular cell* **20**, 117-129 (2005).
57. Takinowaki, H., Matsuda, Y., Yoshida, T., Kobayashi, Y. & Ohkubo, T. The solution structure of the methylated form of the N-terminal 16-kDa domain of *Escherichia coli* Ada protein. *Protein science : a publication of the Protein Society* **15**, 487-497 (2006).
58. Sedgwick, B., Robins, P., Totty, N. & Lindahl, T. Functional domains and methyl acceptor sites of the *Escherichia coli* ada protein. *The Journal of biological chemistry* **263**, 4430-4433 (1988).
59. Stewart, M.D. & Igumenova, T.I. Reactive cysteine in the structural Zn(2+) site of the C1B domain from PKCalpha. *Biochemistry* **51**, 7263-7277 (2012).
60. Chakravarty, S., Zeng, L. & Zhou, M.M. Structure and site-specific recognition of histone H3 by the PHD finger of human autoimmune regulator. *Structure* **17**, 670-679 (2009).
61. Gillooly, D.J. et al. Localization of phosphatidylinositol 3-phosphate in yeast and mammalian cells. *The EMBO journal* **19**, 4577-4588 (2000).
62. Stenmark, H., Aasland, R. & Driscoll, P.C. The phosphatidylinositol 3-phosphate-binding FYVE finger. *FEBS letters* **513**, 77-84 (2002).
63. Lu, J. et al. Structural basis for a Munc13-1 homodimer to Munc13-1/RIM heterodimer switch. *PLoS biology* **4**, e192 (2006).
64. Maddika, S., Sy, S.M. & Chen, J. Functional interaction between Chfr and Kif22 controls genomic stability. *The Journal of biological chemistry* **284**, 12998-13003 (2009).
65. Yu, X. et al. Chfr is required for tumor suppression and Aurora A regulation. *Nature genetics* **37**, 401-406 (2005).
66. Aasland, R., Gibson, T.J. & Stewart, A.F. The PHD finger: implications for chromatin-mediated transcriptional regulation. *Trends in biochemical sciences* **20**, 56-59 (1995).
67. Sanchez, R. & Zhou, M.M. The PHD finger: a versatile epigenome reader. *Trends in biochemical sciences* **36**, 364-372 (2011).
68. Li, Y. & Li, H. Many keys to push: diversifying the 'readership' of plant homeodomain fingers. *Acta biochimica et biophysica Sinica* **44**, 28-39 (2012).
69. Li, H. et al. Structural basis for lower lysine methylation state-specific readout by MBT repeats of L3MBTL1 and an engineered PHD finger. *Molecular cell* **28**, 677-691 (2007).
70. Cheng, J. et al. Structural insight into coordinated recognition of trimethylated histone H3 lysine 9 (H3K9me3) by the plant homeodomain (PHD) and tandem tudor domain (TTD) of UHRF1 (ubiquitin-like, containing PHD and RING finger domains, 1) protein. *The Journal of biological chemistry* **288**, 1329-1339 (2013).
71. Zeng, L. et al. Mechanism and regulation of acetylated histone binding by the tandem PHD finger of DPF3b. *Nature* **466**, 258-262 (2010).
72. Miller, T.C., Rutherford, T.J., Johnson, C.M., Fiedler, M. & Bienz, M. Allosteric remodelling of the histone H3 binding pocket in the Pygo2 PHD finger triggered by its binding to the B9L/BCL9 co-factor. *Journal of molecular biology* **401**, 969-984 (2010).
73. Ivanov, A.V. et al. PHD domain-mediated E3 ligase activity directs intramolecular sumoylation of an adjacent bromodomain required for gene silencing. *Molecular cell* **28**, 823-837 (2007).
74. Liu, L. et al. Solution structure of an atypical PHD finger in BRPF2 and its interaction with DNA. *Journal of structural biology* **180**, 165-173 (2012).
75. Liu, Z. et al. Structural and functional insights into the human Borjeson-Forssman-Lehmann syndrome-associated protein PHF6. *The Journal of biological chemistry* **289**, 10069-10083 (2014).

76. Perry, J. & Zhao, Y. The CW domain, a structural module shared amongst vertebrates, vertebrate-infecting parasites and higher plants. *Trends in biochemical sciences* **28**, 576-580 (2003).
77. He, F. et al. Structural insight into the zinc finger CW domain as a histone modification reader. *Structure* **18**, 1127-1139 (2010).
78. de Souza, R.F., Iyer, L.M. & Aravind, L. Diversity and evolution of chromatin proteins encoded by DNA viruses. *Biochimica et biophysica acta* **1799**, 302-318 (2010).
79. Liu, X., Clements, A., Zhao, K. & Marmorstein, R. Structure of the human Papillomavirus E7 oncoprotein and its mechanism for inactivation of the retinoblastoma tumor suppressor. *The Journal of biological chemistry* **281**, 578-586 (2006).
80. Ohlenschlager, O. et al. Solution structure of the partially folded high-risk human papilloma virus 45 oncoprotein E7. *Oncogene* **25**, 5953-5959 (2006).
81. Bach, I. The LIM domain: regulation by association. *Mechanisms of development* **91**, 5-17 (2000).
82. Langelier, M.F., Planck, J.L., Roy, S. & Pascal, J.M. Crystal structures of poly(ADP-ribose) polymerase-1 (PARP-1) zinc fingers bound to DNA: structural and functional insights into DNA-dependent PARP-1 activity. *The Journal of biological chemistry* **286**, 10690-10701 (2011).
83. Furman, R. et al. DksA2, a zinc-independent structural analog of the transcription factor DksA. *FEBS letters* **587**, 614-619 (2013).
84. He, C. et al. Structural analysis of Stc1 provides insights into the coupling of RNAi and chromatin modification. *Proceedings of the National Academy of Sciences of the United States of America* **110**, E1879-1888 (2013).
85. Jakob, M. et al. Novel DNA-binding element within the C-terminal extension of the nuclear receptor DNA-binding domain. *Nucleic acids research* **35**, 2705-2718 (2007).
86. Khorasanizadeh, S. & Rastinejad, F. Nuclear-receptor interactions on DNA-response elements. *Trends in biochemical sciences* **26**, 384-390 (2001).
87. Van Roey, P., Waddling, C.A., Fox, K.M., Belfort, M. & Derbyshire, V. Intertwined structure of the DNA-binding domain of intron endonuclease I-TevI with its substrate. *The EMBO journal* **20**, 3631-3637 (2001).
88. Sokolowska, M., Czapinska, H. & Bochtler, M. Crystal structure of the beta beta alpha-Me type II restriction endonuclease Hpy99I with target DNA. *Nucleic acids research* **37**, 3799-3810 (2009).
89. Fromme, J.C. & Verdine, G.L. Structural insights into lesion recognition and repair by the bacterial 8-oxoguanine DNA glycosylase MutM. *Nature structural biology* **9**, 544-552 (2002).
90. Omichinski, J.G. et al. NMR structure of a specific DNA complex of Zn-containing DNA binding domain of GATA-1. *Science* **261**, 438-446 (1993).
91. Kadrmas, J.L. & Beckerle, M.C. The LIM domain: from the cytoskeleton to the nucleus. *Nature reviews. Molecular cell biology* **5**, 920-931 (2004).
92. Boeda, B. et al. Molecular recognition of the Tes LIM2-3 domains by the actin-related protein Arp7A. *The Journal of biological chemistry* **286**, 11543-11554 (2011).
93. Ikegami, T. et al. Solution structure of the DNA- and RPA-binding domain of the human repair factor XPA. *Nature structural biology* **5**, 701-706 (1998).
94. Buchko, G.W. et al. Interactions of human nucleotide excision repair protein XPA with DNA and RPA70 Delta C327: chemical shift mapping and 15N NMR relaxation studies. *Biochemistry* **38**, 15116-15128 (1999).
95. Iwase, S. et al. ATRX ADD domain links an atypical histone methylation recognition mechanism to human mental-retardation syndrome. *Nature structural & molecular biology* **18**, 769-776 (2011).
96. Eustermann, S. et al. Combinatorial readout of histone H3 modifications specifies localization of ATRX to heterochromatin. *Nature structural & molecular biology* **18**, 777-782 (2011).
97. Yamasaki, K. et al. A novel zinc-binding motif revealed by solution structures of DNA-binding domains of Arabidopsis SBP-family transcription factors. *Journal of molecular biology* **337**, 49-63 (2004).

98. Grishin, N.V. Mh1 domain of Smad is a degraded homing endonuclease. *Journal of molecular biology* **307**, 31-37 (2001).
99. Friedhoff, P. et al. A similar active site for non-specific and specific endonucleases. *Nature structural biology* **6**, 112-113 (1999).
100. Shen, B.W. et al. Unusual target site disruption by the rare-cutting HNH restriction endonuclease PacI. *Structure* **18**, 734-743 (2010).
101. Gruenig, M.C. et al. Creating directed double-strand breaks with the Ref protein: a novel RecA-dependent nuclease from bacteriophage P1. *The Journal of biological chemistry* **286**, 8240-8251 (2011).
102. Woo, E.J. et al. Structural mechanism for inactivation and activation of CAD/DFF40 in the apoptotic pathway. *Molecular cell* **14**, 531-539 (2004).
103. Welin, M. et al. Structures of thymidine kinase 1 of human and mycoplasmic origin. *Proceedings of the National Academy of Sciences of the United States of America* **101**, 17970-17975 (2004).
104. Kosinska, U. et al. Structural studies of thymidine kinases from *Bacillus anthracis* and *Bacillus cereus* provide insights into quaternary structure and conformational changes upon substrate binding. *The FEBS journal* **274**, 727-737 (2007).
105. Ibba, M., Francklyn, C. & Cusack, S. The Aminoacyl-tRNA Synthetases. (Landes Bioscience, 2005).
106. Yaremchuk, A., Cusack, S. & Tukalo, M. Crystal structure of a eukaryote/archaeon-like protyl-tRNA synthetase and its complex with tRNAPro(CGG). *The EMBO journal* **19**, 4745-4758 (2000).
107. Larson, E.T. et al. Structure of the prolyl-tRNA synthetase from the eukaryotic pathogen *Giardia lamblia*. *Acta crystallographica. Section D, Biological crystallography* **68**, 1194-1200 (2012).
108. Jackson, R.N. et al. Structural biology. Crystal structure of the CRISPR RNA-guided surveillance complex from *Escherichia coli*. *Science* **345**, 1473-1479 (2014).
109. Sashital, D.G., Wiedenheft, B. & Doudna, J.A. Mechanism of foreign DNA selection in a bacterial adaptive immune system. *Molecular cell* **46**, 606-615 (2012).
110. McCleverty, C.J., Hornsby, M., Spraggon, G. & Kreusch, A. Crystal structure of human Pus10, a novel pseudouridine synthase. *Journal of molecular biology* **373**, 1243-1254 (2007).
111. Nandakumar, J., Nair, P.A. & Shuman, S. Last stop on the road to repair: structure of *E. coli* DNA ligase bound to nicked DNA-adenylate. *Molecular cell* **26**, 257-271 (2007).
112. Osborne, M.J., Siddiqui, N., Landgraf, D., Pomposiello, P.J. & Gehring, K. The solution structure of the oxidative stress-related protein YggX from *Escherichia coli*. *Protein science : a publication of the Protein Society* **14**, 1673-1678 (2005).
113. Gralnick, J. & Downs, D. Protection from superoxide damage associated with an increased level of the YggX protein in *Salmonella enterica*. *Proceedings of the National Academy of Sciences of the United States of America* **98**, 8030-8035 (2001).
114. Velayudhan, J. et al. Distinct roles of the Salmonella enterica serovar Typhimurium CyaY and YggX proteins in the biosynthesis and repair of iron-sulfur clusters. *Infection and immunity* **82**, 1390-1401 (2014).
115. Murzin, A.G., Brenner, S.E., Hubbard, T. & Chothia, C. SCOP: a structural classification of proteins database for the investigation of sequences and structures. *Journal of molecular biology* **247**, 536-540 (1995).
116. Stieglitz, B. et al. Structural basis for ligase-specific conjugation of linear ubiquitin chains by HOIP. *Nature* **503**, 422-426 (2013).
117. Smit, J.J. et al. The E3 ligase HOIP specifies linear ubiquitin chain assembly through its RING-IBR-RING domain and the unique LDD extension. *The EMBO journal* **31**, 3833-3844 (2012).
118. Park, E.Y. et al. Structural basis of SspB-tail recognition by the zinc binding domain of ClpX. *Journal of molecular biology* **367**, 514-526 (2007).
119. Donaldson, L.W., Wojtyra, U. & Houry, W.A. Solution structure of the dimeric zinc binding domain of the chaperone ClpX. *The Journal of biological chemistry* **278**, 48991-48996 (2003).

120. Taguchi, Y., Sugishima, M. & Fukuyama, K. Crystal structure of a novel zinc-binding ATP sulfurylase from *Thermus thermophilus HB8*. *Biochemistry* **43**, 4111-4118 (2004).
121. Lee, B.I. et al. Ring-shaped architecture of RecR: implications for its role in homologous recombinational DNA repair. *The EMBO journal* **23**, 2029-2038 (2004).
122. Nakagawa, H. et al. Crystallographic and mutational studies on the tRNA thiouridine synthetase TtuA. *Proteins* **81**, 1232-1244 (2013).
123. Gamsjaeger, R. et al. Structural and biophysical analysis of the DNA binding properties of myelin transcription factor 1. *The Journal of biological chemistry* **283**, 5158-5167 (2008).
124. Berkovits-Cymet, H.J., Amann, B.T. & Berg, J.M. Solution structure of a CCHHC domain of neural zinc finger factor-1 and its implications for DNA binding. *Biochemistry* **43**, 898-903 (2004).
125. Zanier, K. et al. Structural basis for hijacking of cellular LxxLL motifs by papillomavirus E6 oncoproteins. *Science* **339**, 694-698 (2013).
126. Zanier, K. et al. Solution structure analysis of the HPV16 E6 oncoprotein reveals a self-association mechanism required for E6-mediated degradation of p53. *Structure* **20**, 604-617 (2012).
127. Nomine, Y. et al. Structural and functional analysis of E6 oncoprotein: insights in the molecular pathways of human papillomavirus-mediated pathogenesis. *Molecular cell* **21**, 665-678 (2006).
128. Kwan, J.J. et al. The solution structures of two prophage homologues of the bacteriophage lambda Ea8.5 protein reveal a newly discovered hybrid homeodomain/zinc-finger fold. *Biochemistry* **52**, 3612-3614 (2013).
129. Los, J.M., Los, M., Wegrzyn, A. & Wegrzyn, G. Role of the bacteriophage lambda exo-xis region in the virus development. *Folia microbiologica* **53**, 443-450 (2008).
130. Traore, D.A. et al. Crystal structure of the apo-PerR-Zn protein from *Bacillus subtilis*. *Molecular microbiology* **61**, 1211-1219 (2006).
131. Wang, Q. & Rymond, B.C. Rds3p is required for stable U2 snRNP recruitment to the splicing apparatus. *Molecular and cellular biology* **23**, 7339-7349 (2003).
132. van Roon, A.M. et al. Solution structure of the U2 snRNP protein Rds3p reveals a knotted zinc-finger motif. *Proceedings of the National Academy of Sciences of the United States of America* **105**, 9621-9626 (2008).
133. He, Y., Imhoff, R., Sahu, A. & Radhakrishnan, I. Solution structure of a novel zinc finger motif in the SAP30 polypeptide of the Sin3 corepressor complex and its potential role in nucleic acid recognition. *Nucleic acids research* **37**, 2142-2152 (2009).
134. Jauch, R. et al. The zinc finger-associated domain of the Drosophila transcription factor grauzone is a novel zinc-coordinating protein-protein interaction module. *Structure* **11**, 1393-1402 (2003).
135. Sabogal, A., Lyubimov, A.Y., Corn, J.E., Berger, J.M. & Rio, D.C. THAP proteins target specific DNA sites through bipartite recognition of adjacent major and minor grooves. *Nature structural & molecular biology* **17**, 117-123 (2010).
136. Liew, C.K., Crossley, M., Mackay, J.P. & Nicholas, H.R. Solution structure of the THAP domain from Caenorhabditis elegans C-terminal binding protein (CtBP). *Journal of molecular biology* **366**, 382-390 (2007).
137. Roussigne, M. et al. The THAP domain: a novel protein motif with similarity to the DNA-binding domain of P element transposase. *Trends in biochemical sciences* **28**, 66-69 (2003).
138. Guo, X. et al. Structure and function of FusB: an elongation factor G-binding fusidic acid resistance protein active in ribosomal translocation and recycling. *Open biology* **2**, 120016 (2012).
139. Cox, G. et al. Ribosome clearance by FusB-type proteins mediates resistance to the antibiotic fusidic acid. *Proceedings of the National Academy of Sciences of the United States of America* **109**, 2102-2107 (2012).
140. Cocozaki, A.I. et al. Structure of the Cmr2 subunit of the CRISPR-Cas RNA silencing complex. *Structure* **20**, 545-553 (2012).

141. Makarova, K.S., Aravind, L., Wolf, Y.I. & Koonin, E.V. Unification of Cas protein families and a simple scenario for the origin and evolution of CRISPR-Cas systems. *Biology direct* **6**, 38 (2011).
142. Warren, E.M. et al. Structural basis for DNA binding by replication initiator Mcm10. *Structure* **16**, 1892-1901 (2008).
143. Warren, E.M., Huang, H., Fanning, E., Chazin, W.J. & Eichman, B.F. Physical interactions between Mcm10, DNA, and DNA polymerase alpha. *The Journal of biological chemistry* **284**, 24662-24672 (2009).
144. Gordon, B.R., Imperial, R., Wang, L., Navarre, W.W. & Liu, J. Lsr2 of Mycobacterium represents a novel class of H-NS-like proteins. *Journal of bacteriology* **190**, 7052-7059 (2008).
145. Summers, E.L. et al. The structure of the oligomerization domain of Lsr2 from *Mycobacterium tuberculosis* reveals a mechanism for chromosome organization and protection. *PloS one* **7**, e38542 (2012).
146. Qu, Y., Lim, C.J., Whang, Y.R., Liu, J. & Yan, J. Mechanism of DNA organization by Mycobacterium tuberculosis protein Lsr2. *Nucleic acids research* **41**, 5263-5272 (2013).
147. Chen, J.M. et al. Lsr2 of Mycobacterium tuberculosis is a DNA-bridging protein. *Nucleic acids research* **36**, 2123-2135 (2008).
148. Gordon, B.R. et al. Lsr2 is a nucleoid-associated protein that targets AT-rich sequences and virulence genes in Mycobacterium tuberculosis. *Proceedings of the National Academy of Sciences of the United States of America* **107**, 5154-5159 (2010).
149. Finn, R.D. et al. Pfam: the protein families database. *Nucleic acids research* **42**, D222-230 (2014).
150. Cheng, H. et al. ECOD: an evolutionary classification of protein domains. *PLoS computational biology* **10**, e1003926 (2014).
151. Krishna, S.S. & Grishin, N.V. Structurally analogous proteins do exist! *Structure* **12**, 1125-1127 (2004).
152. Mansy, S.S. et al. Structure and evolutionary analysis of a non-biological ATP-binding protein. *Journal of molecular biology* **371**, 501-513 (2007).
153. Simmons, C.R. et al. Three-dimensional structures reveal multiple ADP/ATP binding modes for a synthetic class of artificial proteins. *Biochemistry* **49**, 8689-8699 (2010).
154. Lo Surdo, P., Walsh, M.A. & Sollazzo, M. A novel ADP- and zinc-binding fold from function-directed in vitro evolution. *Nature structural & molecular biology* **11**, 382-383 (2004).
155. Simmons, C.R. et al. A synthetic protein selected for ligand binding affinity mediates ATP hydrolysis. *ACS chemical biology* **4**, 649-658 (2009).
156. Chao, F.A. et al. Structure and dynamics of a primordial catalytic fold generated by in vitro evolution. *Nature chemical biology* **9**, 81-83 (2013).
