# Supplementary material for: Classification of the treble clef zinc finger: noteworthy lessons for structure and function evolution
Source: Sci Rep. 2016 Aug 26;6:32070. doi: 10.1038/srep32070 (PMC4999995; doi:10.1038/srep32070)
Supplement: Supplementary File 2 [file srep32070-s3.pdf]

Title:

Classification of the treble clef zinc finger: noteworthy lessons for structure and function evolution

Authors:

Gurmeet Kaur, Srikrishna Subramanian[1]

Contact Information

CSIR-Institute of Microbial Technology (IMTECH), Sector 39-A, Chandigarh, 160036, India. Phone: +911726665483; Fax: +91-1722695215; Email: krishna@imtech.res.in

[1] Corresponding author

Supplementary File 3

Co-occurring domains with the treble clef zinc fingers. Domain co-occurrence for proteins with structurally characterised TC ZFs is shown. The UniProtId, selected PDBid(s) and the domains present in the full-length sequence of the protein are depicted. Different shapes have represented the different domains.

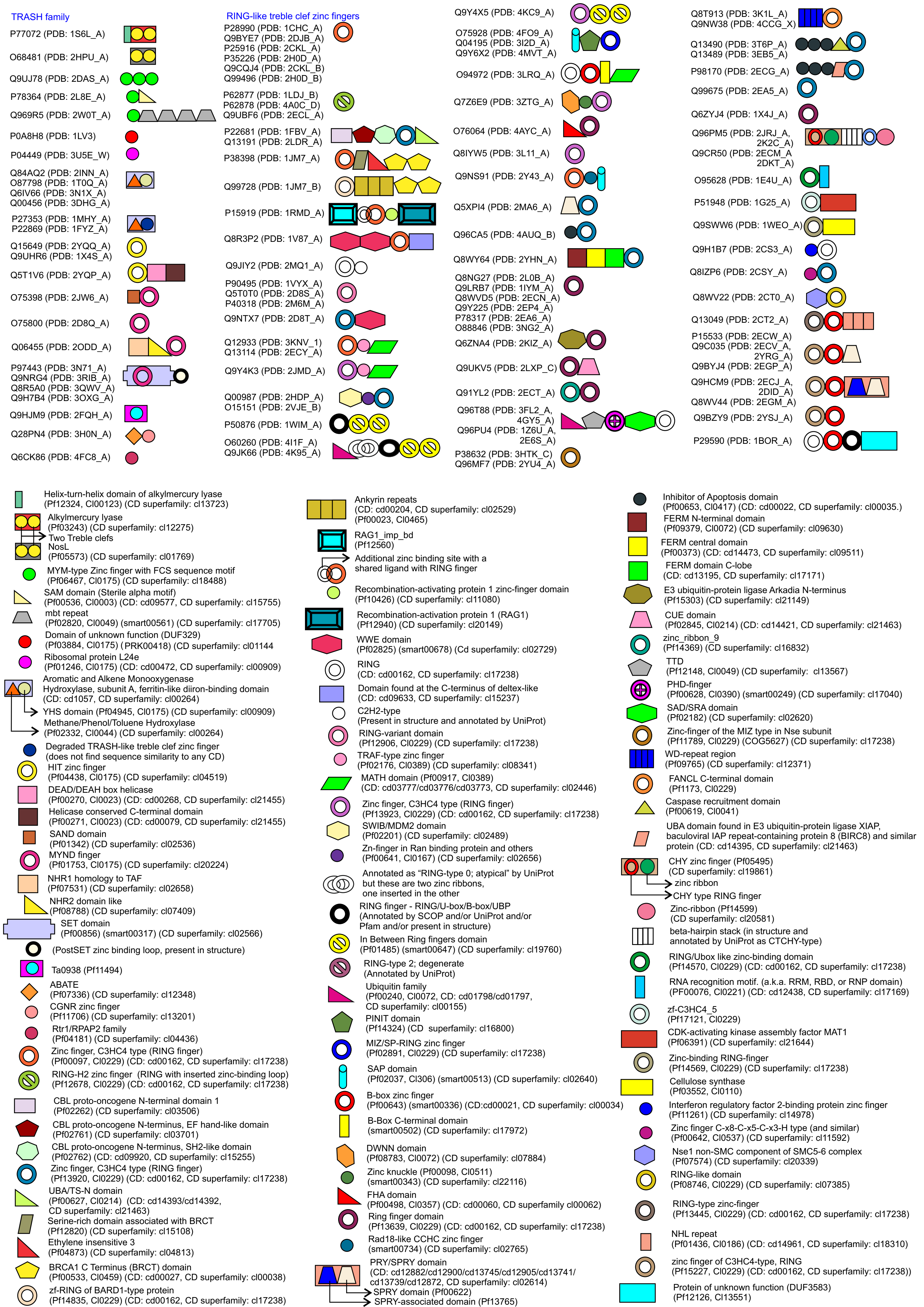
TRASH family
P77072 (PDB: 1S6L_A)
O68481 (PDB: 2HPU_A)
Q9UJ78 (PDB: 2DAS_A)
P78364 (PDB: 2L8E_A)
Q969R5 (PDB: 2W0T_A)
P0A8H8 (PDB: 1LV3)
P04449 (PDB: 3U5E_W)
Q84AQ2 (PDB: 2INN_A)
O87798 (PDB: 1T0Q_A)
Q6IV66 (PDB: 3N1X_A)
Q00456 (PDB: 3DHG_A)
P27353 (PDB: 1MHY_A)
P22869 (PDB: 1FYZ_A)
Q15649 (PDB: 2YQQ_A)
Q9UHR6 (PDB: 1X4S_A)
Q5T1V6 (PDB: 2YQP_A)
O75398 (PDB: 2JW6_A)
O75800 (PDB: 2D8Q_A)
Q06455 (PDB: 2ODD_A)
P97443 (PDB: 3N71_A)
Q9NRG4 (PDB: 3RIB_A)
Q8R5A0 (PDB: 3QWV_A)
Q9H7B4 (PDB: 3OXG_A)
Q9HJM9 (PDB: 2FQH_A)
Q28PN4 (PDB: 3H0N_A)
Q6CK86 (PDB: 4FC8_A)
RING-like treble clef zinc fingers
P28990 (PDB: 1CHC_A)
Q9BYE7 (PDB: 2DJB_A)
P25916 (PDB: 2CKL_A)
P35226 (PDB: 2H0D_A)
Q9CQJ4 (PDB: 2CKL_B)
Q99496 (PDB: 2H0D_B)
P62877 (PDB: 1LDJ_B)
P62878 (PDB: 4A0C_D)
Q9UBF6 (PDB: 2ECL_A)
P22681 (PDB: 1FBV_A)
Q13191 (PDB: 2LDR_A)
P38398 (PDB: 1JM7_A)
Q99728 (PDB: 1JM7_B)
P15919 (PDB: 1RMD_A)
Q8R3P2 (PDB: 1V87_A)
Q9JIY2 (PDB: 2MQ1_A)
P90495 (PDB: 1VYX_A)
Q5T0T0 (PDB: 2D8S_A)
P40318 (PDB: 2M6M_A)
Q9NTX7 (PDB: 2D8T_A)
Q12933 (PDB: 3KNV_1)
Q13114 (PDB: 2ECY_A)
Q9Y4K3 (PDB: 2JMD_A)
Q00987 (PDB: 2HDP_A)
O15151 (PDB: 2VJE_B)
P50876 (PDB: 1WIM_A)
O60260 (PDB: 4I1F_A)
Q9JK66 (PDB: 4K95_A)
Q9Y4X5 (PDB: 4KC9_A)
O75928 (PDB: 4FO9_A)
Q04195 (PDB: 3I2D_A)
Q9Y6X2 (PDB: 4MVT_A)
O94972 (PDB: 3LRQ_A)
Q7Z6E9 (PDB: 3ZTG_A)
O76064 (PDB: 4AYC_A)
Q8IYW5 (PDB: 3L11_A)
Q9NS91 (PDB: 2Y43_A)
Q5XPI4 (PDB: 2MA6_A)
Q96CA5 (PDB: 4AUQ_B)
Q8WY64 (PDB: 2YHN_A)
Q8NG27 (PDB: 2L0B_A)
Q9LRB7 (PDB: 1IYM_A)
Q8WVD5 (PDB: 2ECN_A)
Q9Y225 (PDB: 2EP4_A)
P78317 (PDB: 2EA6_A)
O88846 (PDB: 3NG2_A)
Q6ZNA4 (PDB: 2KIZ_A)
Q9UKV5 (PDB: 2LXP_C)
Q91YL2 (PDB: 2ECT_A)
Q96T88 (PDB: 3FL2_A, 4GY5_A)
Q96PU4 (PDB: 1Z6U_A, 2E6S_A)
P38632 (PDB: 3HTK_C)
Q96MF7 (PDB: 2YU4_A)
Q8T913 (PDB: 3K1L_A)
Q9NW38 (PDB: 4CCG_X)
Q13490 (PDB: 3T6P_A)
Q13489 (PDB: 3EB5_A)
P98170 (PDB: 2ECG_A)
Q99675 (PDB: 2EA5_A)
Q6ZYJ4 (PDB: 1X4J_A)
Q96PM5 (PDB: 2JRJ_A, 2K2C_A)
Q9CR50 (PDB: 2ECM_A 2DKT_A)
O95628 (PDB: 1E4U_A)
P51948 (PDB: 1G25_A)
Q9SWW6 (PDB: 1WEO_A)
Q9H1B7 (PDB: 2CS3_A)
Q8IZP6 (PDB: 2CSY_A)
Q8WV22 (PDB: 2CT0_A)
Q13049 (PDB: 2CT2_A)
P15533 (PDB: 2ECW_A)
Q9C035 (PDB: 2ECV_A, 2YRG_A)
Q9BYJ4 (PDB: 2EGP_A)
Q9HCM9 (PDB: 2ECJ_A, 2DID_A)
Q8WV44 (PDB: 2EGM_A)
Q9BZY9 (PDB: 2YSJ_A)
P29590 (PDB: 1BOR_A)
Helix-turn-helix domain of alkylmercury lyase (Pf12324, Cl00123) (CD superfamily: cl13723)
Alkylmercury lyase (Pf03243) (CD superfamily: cl12275)
Two Treble clefs
NosL (Pf05573) (CD superfamily: cl01769)
MYM-type Zinc finger with FCS sequence motif (Pf06467, Cl0175) (CD superfamily: cl18488)
SAM domain (Sterile alpha motif) (Pf00536, Cl0003) (CD: cd09577, CD superfamily: cl15755)
mbt repeat (Pf02820, Cl0049) (smart00561) (CD superfamily: cl17705)
Domain of unknown function (DUF329) (Pf03884, Cl0175) (PRK00418) (CD superfamily: cl01144
Ribosomal protein L24e (Pf01246, Cl0175) (CD: cd00472, CD superfamily: cl00909)
Aromatic and Alkene Monooxygenase Hydroxylase, subunit A, ferritin-like diiron-binding domain (CD: cd1057, CD superfamily: cl00264)
YHS domain (Pf04945, Cl0175) (CD superfamily: cl00909)
Methane/Phenol/Toluene Hydroxylase (Pf02332, Cl0044) (CD superfamily: cl00264)
Degraded TRASH-like treble clef zinc finger (does not find sequence similarity to any CD)
HIT zinc finger (Pf04438, Cl0175) (CD superfamily: cl04519)
DEAD/DEAH box helicase (Pf00270, Cl0023) (CD: cd00268, CD superfamily: cl21455)
Helicase conserved C-terminal domain (Pf00271, Cl0023) (CD: cd00079, CD superfamily: cl21455)
SAND domain (Pf01342) (CD superfamily: cl02536)
MYND finger (Pf01753, Cl0175) (CD superfamily: cl20224)
NHR1 homology to TAF (Pf07531) (CD superfamily: cl02658)
NHR2 domain like (Pf08788) (CD superfamily: cl07409)
SET domain (Pf00856) (smart00317) (CD superfamily: cl02566)
(PostSET zinc binding loop, present in structure)
Ta0938 (Pf11494)
ABATE (Pf07336) (CD superfamily: cl12348)
CGNR zinc finger (Pf11706) (CD superfamily: cl13201)
Rtr1/RPAP2 family (Pf04181) (CD superfamily: cl04436)
Zinc finger, C3HC4 type (RING finger) (Pf00097, Cl0229) (CD: cd00162, CD superfamily: cl17238)
RING-H2 zinc finger (RING with inserted zinc-binding loop) (Pf12678, Cl0229) (CD: cd00162, CD superfamily: cl17238)
CBL proto-oncogene N-terminal domain 1 (Pf02262) (CD superfamily: cl03506)
CBL proto-oncogene N-terminus, EF hand-like domain (Pf02761) (CD superfamily: cl03701)
CBL proto-oncogene N-terminus, SH2-like domain (Pf02762) (CD: cd09920, CD superfamily: cl15255)
Zinc finger, C3HC4 type (RING finger) (Pf13920, Cl0229) (CD: cd00162, CD superfamily: cl17238)
UBA/TS-N domain (Pf00627, Cl0214) (CD: cd14393/cd14392, CD superfamily: cl21463)
Serine-rich domain associated with BRCT (Pf12820) (CD superfamily: cl15108)
Ethylene insensitive 3 (Pf04873) (CD superfamily: cl04813)
BRCA1 C Terminus (BRCT) domain (Pf00533, Cl0459) (CD: cd00027, CD superfamily: cl00038)
zf-RING of BARD1-type protein (Pf14835, Cl0229) (CD: cd00162, CD superfamily: cl17238)
Ankyrin repeats (CD: cd00204, CD superfamily: cl02529) (Pf00023, Cl0465)
RAG1_imp_bd (Pf12560)
Additional zinc binding site with a shared ligand with RING finger
Recombination-activating protein 1 zinc-finger domain (Pf10426) (CD superfamily: cl11080)
Recombination-activation protein 1 (RAG1) (Pf12940) (CD superfamily: cl20149)
WWE domain (Pf02825) (smart00678) (Cd superfamily: cl02729)
RING (CD: cd00162, CD superfamily: cl17238)
Domain found at the C-terminus of deltex-like (CD: cd09633, CD superfamily: cl15237)
C2H2-type (Present in structure and annotated by UniProt)
RING-variant domain (Pf12906, Cl0229) (CD superfamily: cl17238)
TRAF-type zinc finger (Pf02176, Cl0389) (CD superfamily: cl08341)
MATH domain (Pf00917, Cl0389) (CD: cd03777/cd03776/cd03773, CD superfamily: cl02446)
Zinc finger, C3HC4 type (RING finger) (Pf13923, Cl0229) (CD: cd00162, CD superfamily: cl17238)
SWIB/MDM2 domain (Pf02201) (CD superfamily: cl02489)
Zn-finger in Ran binding protein and others (Pf00641, Cl0167) (CD superfamily: cl02656)
Annotated as “RING-type 0; atypical” by UniProt but these are two zinc ribbons, one inserted in the other
RING finger - RING/U-box/B-box/UBP (Annotated by SCOP and/or UniProt and/or Pfam and/or present in structure)
In Between Ring fingers domain (Pf01485) (smart00647) (CD superfamily: cl19760)
RING-type 2; degenerate (Annotated by UniProt)
Ubiquitin family (Pf00240, Cl0072, CD: cd01798/cd01797, CD superfamily: cl00155)
PINIT domain (Pf14324) (CD superfamily: cl16800)
MIZ/SP-RING zinc finger (Pf02891, Cl0229) (CD superfamily: cl17238)
SAP domain (Pf02037, Cl306) (smart00513) (CD superfamily: cl02640)
B-box zinc finger (Pf00643) (smart00336) (CD:cd00021, CD superfamily: cl00034)
B-Box C-terminal domain (smart00502) (CD superfamily: cl17972)
DWNN domain (Pf08783, Cl0072) (CD superfamily: cl07884)
Zinc knuckle (Pf00098, Cl0511) (smart00343) (CD superfamily: cl22116)
FHA domain (Pf00498, Cl0357) (CD: cd00060, CD superfamily cl00062)
Ring finger domain (Pf13639, Cl0229) (CD: cd00162, CD superfamily: cl17238)
Rad18-like CCHC zinc finger (smart00734) (CD superfamily: cl02765)
PRY/SPRY domain (CD: cd12882/cd12900/cd13745/cd12905/cd13741/ cd13739/cd12872, CD superfamily: cl02614)
SPRY domain (Pf00622)
SPRY-associated domain (Pf13765)
Inhibitor of Apoptosis domain (Pf00653, Cl0417) (CD: cd00022, CD superfamily: cl00035.)
FERM N-terminal domain (Pf09379, Cl0072) (CD superfamily: cl09630)
FERM central domain (Pf00373) (CD: cd14473, CD superfamily: cl09511)
FERM domain C-lobe (CD: cd13195, CD superfamily: cl17171)
E3 ubiquitin-protein ligase Arkadia N-terminus (Pf15303) (CD superfamily: cl21149)
CUE domain (Pf02845, Cl0214) (CD: cd14421, CD superfamily: cl21463)
zinc_ribbon_9 (Pf14369) (CD superfamily: cl16832)
TTD (Pf12148, Cl0049) (CD superfamily: cl13567)
PHD-finger (Pf00628, Cl0390) (smart00249) (CD superfamily: cl17040)
SAD/SRA domain (Pf02182) (CD superfamily: cl02620)
Zinc-finger of the MIZ type in Nse subunit (Pf11789, Cl0229) (COG5627) (CD superfamily: cl17238)
WD-repeat region (Pf09765) (CD superfamily: cl12371)
FANCL C-terminal domain (Pf1173, Cl0229)
Caspase recruitment domain (Pf00619, Cl0041)
UBA domain found in E3 ubiquitin-protein ligase XIAP, baculoviral IAP repeat-containing protein 8 (BIRC8) and similar protein (CD: cd14395, CD superfamily: cl21463)
CHY zinc finger (Pf05495) (CD superfamily: cl19861)
zinc ribbon
CHY type RING finger
Zinc-ribbon (Pf14599) (CD superfamily: cl20581)
beta-hairpin stack (in structure and annotated by UniProt as CTCHY-type)
RING/Ubox like zinc-binding domain (Pf14570, Cl0229) (CD: cd00162, CD superfamily: cl17238)
RNA recognition motif. (a.k.a. RRM, RBD, or RNP domain) (PF00076, Cl0221) (CD: cd12438, CD superfamily: cl17169)
zf-C3HC4_5 (Pf17121, Cl0229)
CDK-activating kinase assembly factor MAT1 (Pf06391) (CD superfamily: cl21644)
Zinc-binding RING-finger (Pf14569, Cl0229) (CD superfamily: cl17238)
Cellulose synthase (Pf03552, Cl0110)
Interferon regulatory factor 2-binding protein zinc finger (Pf11261) (CD superfamily: cl14978)
Zinc finger C-x8-C-x5-C-x3-H type (and similar) (Pf00642, Cl0537) (CD superfamily: cl11592)
Nse1 non-SMC component of SMC5-6 complex (Pf07574) (CD superfamily: cl20339)
RING-like domain (Pf08746, Cl0229) (CD superfamily: cl07385)
RING-type zinc-finger (Pf13445, Cl0229) (CD: cd00162, CD superfamily: cl17238)
NHL repeat (Pf01436, Cl0186) (CD: cd14961, CD superfamily: cl18310)
zinc finger of C3HC4-type, RING (Pf15227, Cl0229) (CD: cd00162, CD superfamily: cl17238))
Protein of unknown function (DUF3583) (Pf12126, Cl13551)

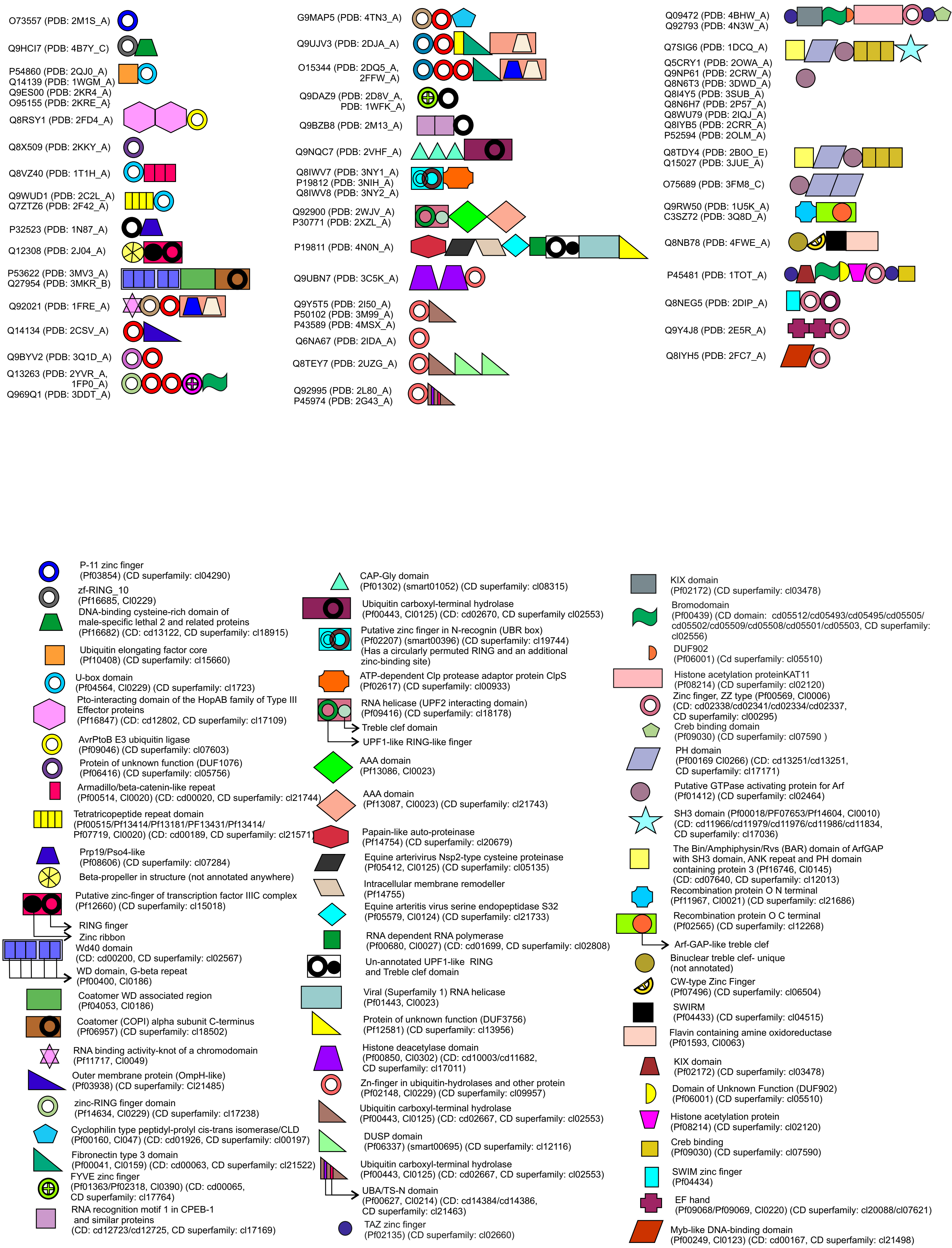
O73557 (PDB: 2M1S_A)
Q9HCI7 (PDB: 4B7Y_C)
P54860 (PDB: 2QJ0_A)
Q14139 (PDB: 1WGM_A)
Q9ES00 (PDB: 2KR4_A)
O95155 (PDB: 2KRE_A}
Q8RSY1 (PDB: 2FD4_A)
Q8X509 (PDB: 2KKY_A)
Q8VZ40 (PDB: 1T1H_A)
Q9WUD1 (PDB: 2C2L_A)
Q7ZTZ6 (PDB: 2F42_A)
P32523 (PDB: 1N87_A)
Q12308 (PDB: 2J04_A)
P53622 (PDB: 3MV3_A)
Q27954 (PDB: 3MKR_B)
Q92021 (PDB: 1FRE_A)
Q14134 (PDB: 2CSV_A)
Q9BYV2 (PDB: 3Q1D_A)
Q13263 (PDB: 2YVR_A, 1FP0_A)
Q969Q1 (PDB: 3DDT_A)
G9MAP5 (PDB: 4TN3_A)
Q9UJV3 (PDB: 2DJA_A)
O15344 (PDB: 2DQ5_A, 2FFW_A)
Q9DAZ9 (PDB: 2D8V_A, PDB: 1WFK_A)
Q9BZB8 (PDB: 2M13_A)
Q9NQC7 (PDB: 2VHF_A)
Q8IWV7 (PDB: 3NY1_A)
P19812 (PDB: 3NIH_A)
Q8IWV8 (PDB: 3NY2_A)
Q92900 (PDB: 2WJV_A)
P30771 (PDB: 2XZL_A)
P19811 (PDB: 4N0N_A)
Q9UBN7 (PDB: 3C5K_A)
Q9Y5T5 (PDB: 2I50_A)
P50102 (PDB: 3M99_A)
P43589 (PDB: 4MSX_A)
Q6NA67 (PDB: 2IDA_A)
Q8TEY7 (PDB: 2UZG_A)
Q92995 (PDB: 2L80_A)
P45974 (PDB: 2G43_A)
Q09472 (PDB: 4BHW_A)
Q92793 (PDB: 4N3W_A)
Q7SIG6 (PDB: 1DCQ_A)
Q5CRY1 (PDB: 2OWA_A)
Q9NP61 (PDB: 2CRW_A)
Q8N6T3 (PDB: 3DWD_A)
Q8I4Y5 (PDB: 3SUB_A)
Q8N6H7 (PDB: 2P57_A)
Q8WU79 (PDB: 2IQJ_A)
Q8IYB5 (PDB: 2CRR_A)
P52594 (PDB: 2OLM_A)
Q8TDY4 (PDB: 2B0O_E)
Q15027 (PDB: 3JUE_A)
O75689 (PDB: 3FM8_C)
Q9RW50 (PDB: 1U5K_A)
C3SZ72 (PDB: 3Q8D_A)
Q8NB78 (PDB: 4FWE_A)
P45481 (PDB: 1TOT_A)
Q8NEG5 (PDB: 2DIP_A)
Q9Y4J8 (PDB: 2E5R_A)
Q8IYH5 (PDB: 2FC7_A)
P-11 zinc finger (Pf03854) (CD superfamily: cl04290)
zf-RING_10 (Pf16685, Cl0229)
DNA-binding cysteine-rich domain of male-specific lethal 2 and related proteins (Pf16682) (CD: cd13122, CD superfamily: cl18915)
Ubiquitin elongating factor core (Pf10408) (CD superfamily: cl15660)
U-box domain (Pf04564, Cl0229) (CD superfamily: cl1723)
Pto-interacting domain of the HopAB family of Type III Effector proteins (Pf16847) (CD: cd12802, CD superfamily: cl17109)
AvrPtoB E3 ubiquitin ligase (Pf09046) (CD superfamily: cl07603)
Protein of unknown function (DUF1076) (Pf06416) (CD superfamily: cl05756)
Armadillo/beta-catenin-like repeat (Pf00514, Cl0020) (CD: cd00020, CD superfamily: cl21744)
Tetratricopeptide repeat domain (Pf00515/Pf13414/Pf13181/Pf13431/Pf13414/ Pf07719, Cl0020) (CD: cd00189, CD superfamily: cl21571)
Prp19/Pso4-like (Pf08606) (CD superfamily: cl07284)
Beta-propeller in structure (not annotated anywhere)
Putative zinc-finger of transcription factor IIIC complex (Pf12660) (CD superfamily: cl15018)
RING finger
Zinc ribbon
Wd40 domain (CD: cd00200, CD superfamily: cl02567)
WD domain, G-beta repeat (Pf00400, Cl0186)
Coatomer WD associated region (Pf04053, Cl0186)
Coatomer (COPI) alpha subunit C-terminus (Pf06957) (CD superfamily: cl18502)
RNA binding activity-knot of a chromodomain (Pf11717, Cl0049)
Outer membrane protein (OmpH-like) (Pf03938) (CD superfamily: Cl21485)
zinc-RING finger domain (Pf14634, Cl0229) (CD superfamily: cl17238)
Cyclophilin type peptidyl-prolyl cis-trans isomerase/CLD (Pf00160, Cl047) (CD: cd01926, CD superfamily: cl00197)
Fibronectin type 3 domain (Pf00041, Cl0159) (CD: cd00063, CD superfamily: cl21522)
FYVE zinc finger (Pf01363/Pf02318, Cl0390) (CD: cd00065, CD superfamily: cl17764)
RNA recognition motif 1 in CPEB-1 and similar proteins (CD: cd12723/cd12725, CD superfamily: cl17169)
CAP-Gly domain (Pf01302) (smart01052) (CD superfamily: cl08315)
Ubiquitin carboxyl-terminal hydrolase (Pf00443, Cl0125) (CD: cd02670, CD superfamily cl02553)
Putative zinc finger in N-recognin (UBR box) (Pf02207) (smart00396) (CD superfamily: cl19744) (Has a circularly permuted RING and an additional zinc-binding site)
ATP-dependent Clp protease adaptor protein ClpS (Pf02617) (CD superfamily: cl00933)
RNA helicase (UPF2 interacting domain) (Pf09416) (CD superfamily: cl18178)
Treble clef domain
UPF1-like RING-like finger
AAA domain (Pf13086, Cl0023)
AAA domain (Pf13087, Cl0023) (CD superfamily: cl21743)
Papain-like auto-proteinase (Pf14754) (CD superfamily: cl20679)
Equine arterivirus Nsp2-type cysteine proteinase (Pf05412, Cl0125) (CD superfamily: cl05135)
Intracellular membrane remodeller (Pf14755)
Equine arteritis virus serine endopeptidase S32 (Pf05579, Cl0124) (CD superfamily: cl21733)
RNA dependent RNA polymerase (Pf00680, Cl0027) (CD: cd01699, CD superfamily: cl02808)
Un-annotated UPF1-like RING and Treble clef domain
Viral (Superfamily 1) RNA helicase (Pf01443, Cl0023)
Protein of unknown function (DUF3756) (Pf12581) (CD superfamily: cl13956)
Histone deacetylase domain (Pf00850, Cl0302) (CD: cd10003/cd11682, CD superfamily: cl17011)
Zn-finger in ubiquitin-hydrolases and other protein (Pf02148, Cl0229) (CD superfamily: cl09957)
Ubiquitin carboxyl-terminal hydrolase (Pf00443, Cl0125) (CD: cd02667, CD superfamily: cl02553)
DUSP domain (Pf06337) (smart00695) (CD superfamily: cl12116)
Ubiquitin carboxyl-terminal hydrolase (Pf00443, Cl0125) (CD: cd02667, CD superfamily: cl02553)
UBA/TS-N domain (Pf00627, Cl0214) (CD: cd14384/cd14386, CD superfamily: cl21463)
TAZ zinc finger (Pf02135) (CD superfamily: cl02660)
KIX domain (Pf02172) (CD superfamily: cl03478)
Bromodomain (Pf00439) (CD domain: cd05512/cd05493/cd05495/cd05505/ cd05502/cd05509/cd05508/cd05501/cd05503, CD superfamily: cl02556)
DUF902 (Pf06001) (Cd superfamily: cl05510)
Histone acetylation proteinKAT11 (Pf08214) (CD superfamily: cl02120)
Zinc finger, ZZ type (Pf00569, Cl0006) (CD: cd02338/cd02341/cd02334/cd02337, CD superfamily: cl00295)
Creb binding domain (Pf09030) (CD superfamily: cl07590)
PH domain (Pf00169 Cl0266) (CD: cd13251/cd13251, CD superfamily: cl17171)
Putative GTPase activating protein for Arf (Pf01412) (CD superfamily: cl02464)
SH3 domain (Pf00018/PF07653/Pf14604, Cl0010) (CD: cd11966/cd11979/cd11976/cd11986/cd11834, CD superfamily: cl17036)
The Bin/Amphiphysin/Rvs (BAR) domain of ArfGAP with SH3 domain, ANK repeat and PH domain containing protein 3 (Pf16746, Cl0145) (CD: cd07640, CD superfamily: cl12013)
Recombination protein O N terminal (Pf11967, Cl0021) (CD superfamily: cl21686)
Recombination protein O C terminal (Pf02565) (CD superfamily: cl12268)
Arf-GAP-like treble clef
Binuclear treble clef- unique (not annotated)
CW-type Zinc Finger (Pf07496) (CD superfamily: cl06504)
SWIRM (Pf04433) (CD superfamily: cl04515)
Flavin containing amine oxidoreductase (Pf01593, Cl0063)
KIX domain (Pf02172) (CD superfamily: cl03478)
Domain of Unknown Function (DUF902) (Pf06001) (CD superfamily: cl05510)
Histone acetylation protein (Pf08214) (CD superfamily: cl02120)
Creb binding (Pf09030) (CD superfamily: cl07590)
SWIM zinc finger (Pf04434)
EF hand (Pf09068/Pf09069, Cl0220) (CD superfamily: cl20088/cl07621)
Myb-like DNA-binding domain (Pf00249, Cl0123) (CD: cd00167, CD superfamily: cl21498)

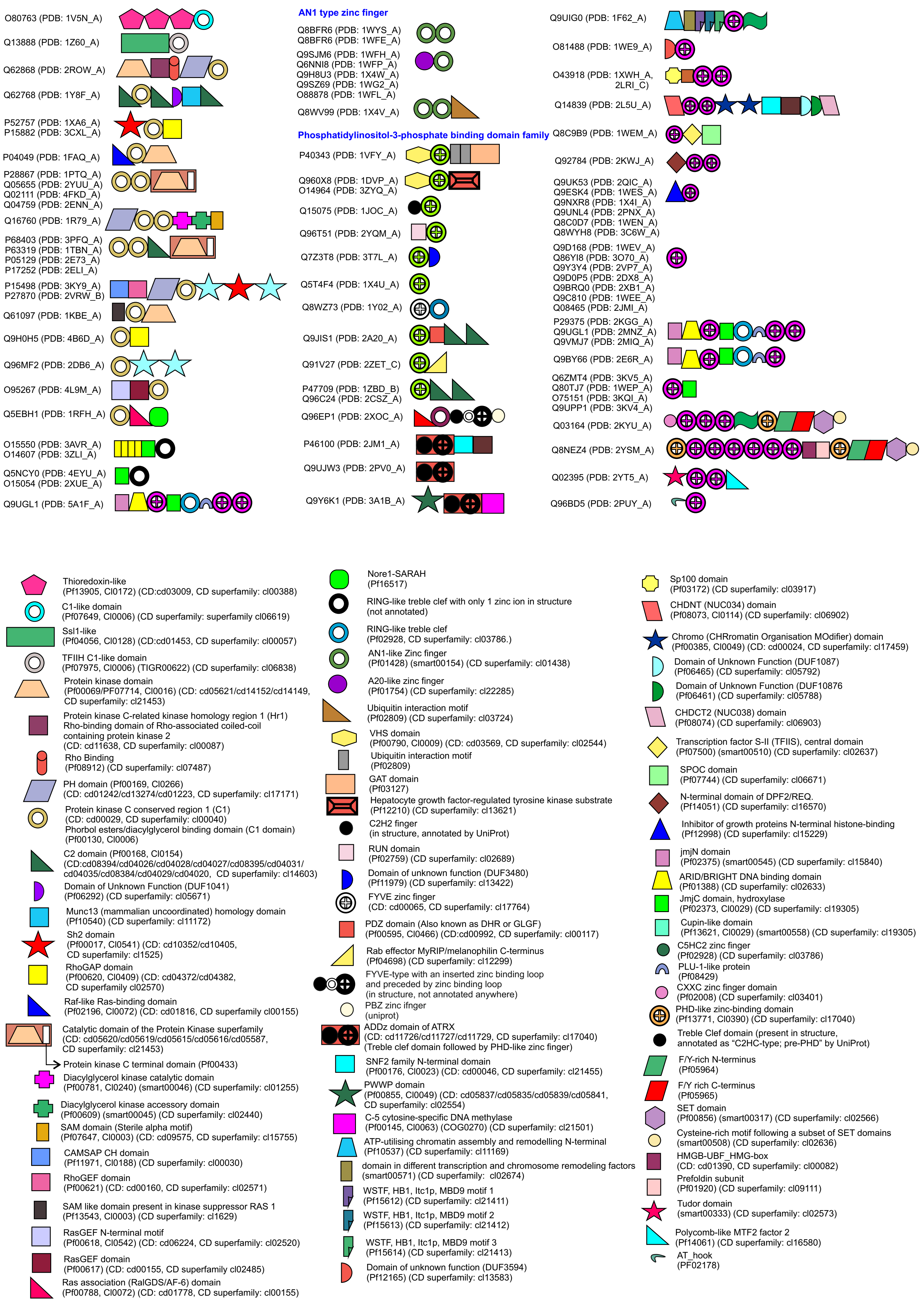

O80763 (PDB: 1V5N_A)
Q13888 (PDB: 1Z60_A)
Q62868 (PDB: 2ROW_A)
Q62768 (PDB: 1Y8F_A)
P52757 (PDB: 1XA6_A)
P15882 (PDB: 3CXL_A)
P04049 (PDB: 1FAQ_A)
P28867 (PDB: 1PTQ_A)
Q05655 (PDB: 2YUU_A)
Q02111 (PDB: 4FKD_A)
Q04759 (PDB: 2ENN_A)
Q16760 (PDB: 1R79_A)
P68403 (PDB: 3PFQ_A)
P63319 (PDB: 1TBN_A)
P05129 (PDB: 2E73_A)
P17252 (PDB: 2ELI_A)
P15498 (PDB: 3KY9_A)
P27870 (PDB: 2VRW_B)
Q61097 (PDB: 1KBE_A)
Q9H0H5 (PDB: 4B6D_A)
Q96MF2 (PDB: 2DB6_A)
O95267 (PDB: 4L9M_A)
Q5EBH1 (PDB: 1RFH_A)
O15550 (PDB: 3AVR_A)
O14607 (PDB: 3ZLI_A)
Q5NCY0 (PDB: 4EYU_A)
O15054 (PDB: 2XUE_A)
Q9UGL1 (PDB: 5A1F_A)
AN1 type zinc finger
Q8BFR6 (PDB: 1WYS_A)
Q8BFR6 (PDB: 1WFE_A)
Q9SJM6 (PDB: 1WFH_A)
Q6NNI8 (PDB: 1WFP_A)
Q9H8U3 (PDB: 1X4W_A)
Q9SZ69 (PDB: 1WG2_A)
O88878 (PDB: 1WFL_A)
Q8WV99 (PDB: 1X4V_A)
Phosphatidylinositol-3-phosphate binding domain family
P40343 (PDB: 1VFY_A)
Q960X8 (PDB: 1DVP_A)
O14964 (PDB: 3ZYQ_A)
Q15075 (PDB: 1JOC_A)
Q96T51 (PDB: 2YQM_A)
Q7Z3T8 (PDB: 3T7L_A)
Q5T4F4 (PDB: 1X4U_A)
Q8WZ73 (PDB: 1Y02_A)
Q9JIS1 (PDB: 2A20_A)
Q91V27 (PDB: 2ZET_C)
P47709 (PDB: 1ZBD_B)
Q96C24 (PDB: 2CSZ_A)
Q96EP1 (PDB: 2XOC_A)
P46100 (PDB: 2JM1_A)
Q9UJW3 (PDB: 2PV0_A)
Q9Y6K1 (PDB: 3A1B_A)
Q9UIG0 (PDB: 1F62_A)
O81488 (PDB: 1WE9_A)
O43918 (PDB: 1XWH_A, 2LRI_C)
Q14839 (PDB: 2L5U_A)
Q8C9B9 (PDB: 1WEM_A)
Q92784 (PDB: 2KWJ_A)
Q9UK53 (PDB: 2QIC_A)
Q9ESK4 (PDB: 1WES_A)
Q9NXR8 (PDB: 1X4I_A)
Q9UNL4 (PDB: 2PNX_A)
Q8C0D7 (PDB: 1WEN_A)
Q8WYH8 (PDB: 3C6W_A)
Q9D168 (PDB: 1WEV_A)
Q86YI8 (PDB: 3O70_A)
Q9Y3Y4 (PDB: 2VP7_A)
Q9D0P5 (PDB: 2DX8_A)
Q9BRQ0 (PDB: 2XB1_A)
Q9C810 (PDB: 1WEE_A)
Q08465 (PDB: 2JMI_A)
P29375 (PDB: 2KGG_A)
Q9UGL1 (PDB: 2MNZ_A)
Q9VMJ7 (PDB: 2MIQ_A)
Q9BY66 (PDB: 2E6R_A)
Q6ZMT4 (PDB: 3KV5_A)
Q80TJ7 (PDB: 1WEP_A)
O75151 (PDB: 3KQI_A)
Q9UPP1 (PDB: 3KV4_A)
Q03164 (PDB: 2KYU_A)
Q8NEZ4 (PDB: 2YSM_A)
Q02395 (PDB: 2YT5_A)
Q96BD5 (PDB: 2PUY_A)
Thioredoxin-like (Pf13905, Cl0172) (CD:cd03009, CD superfamily: cl00388)
C1-like domain (Pf07649, Cl0006) (CD superfamily: superfamily cl06619)
Ssl1-like (Pf04056, Cl0128) (CD:cd01453, CD superfamily: cl00057)
TFIIH C1-like domain (Pf07975, Cl0006) (TIGR00622) (CD superfamily: cl06838)
Protein kinase domain (Pf00069/PF07714, Cl0016) (CD: cd05621/cd14152/cd14149, CD superfamily: cl21453)
Protein kinase C-related kinase homology region 1 (Hr1) Rho-binding domain of Rho-associated coiled-coil containing protein kinase 2 (CD: cd11638, CD superfamily: cl00087)
Rho Binding (Pf08912) (CD superfamily: cl07487)
PH domain (Pf00169, Cl0266) (CD: cd01242/cd13274/cd01223, CD superfamily: cl17171)
Protein kinase C conserved region 1 (C1) (CD: cd00029, CD superfamily: cl00040) Phorbol esters/diacylglycerol binding domain (C1 domain) (Pf00130, Cl0006)
C2 domain (Pf00168, Cl0154) (CD:cd08394/cd04026/cd04028/cd04027/cd08395/cd04031/ cd04035/cd08384/cd04029/cd04020, CD superfamily: cl14603)
Domain of Unknown Function (DUF1041) (Pf06292) (CD superfamily: cl05671)
Munc13 (mammalian uncoordinated) homology domain (Pf10540) (CD superfamily: cl11172)
Sh2 domain (Pf00017, Cl0541) (CD: cd10352/cd10405, CD superfamily: cl1525)
RhoGAP domain (Pf00620, Cl0409) (CD: cd04372/cd04382, CD superfamily cl02570)
Raf-like Ras-binding domain (Pf02196, Cl0072) (CD: cd01816, CD superfamily cl00155)
Catalytic domain of the Protein Kinase superfamily (CD: cd05620/cd05619/cd05615/cd05616/cd05587, CD superfamily: cl21453)
Protein kinase C terminal domain (Pf00433)
Diacylglycerol kinase catalytic domain (Pf00781, Cl0240) (smart00046) (CD superfamily: cl01255)
Diacylglycerol kinase accessory domain (Pf00609) (smart00045) (CD superfamily: cl02440)
SAM domain (Sterile alpha motif) (Pf07647, Cl0003) (CD: cd09575, CD superfamily: cl15755)
CAMSAP CH domain (Pf11971, Cl0188) (CD superfamily: cl00030)
RhoGEF domain (Pf00621) (CD: cd00160, CD superfamily: cl02571)
SAM like domain present in kinase suppressor RAS 1 (Pf13543, Cl0003) (CD superfamily: cl1629)
RasGEF N-terminal motif (Pf00618, Cl0542) (CD: cd06224, CD superfamily: cl02520)
RasGEF domain (Pf00617) (CD: cd00155, CD superfamily cl02485)
Ras association (RalGDS/AF-6) domain (Pf00788, Cl0072) (CD: cd01778, CD superfamily: cl00155)
Nore1-SARAH (Pf16517)
RING-like treble clef with only 1 zinc ion in structure (not annotated)
RING-like treble clef (Pf02928, CD superfamily: cl03786.)
AN1-like Zinc finger (Pf01428) (smart00154) (CD superfamily: cl01438)
A20-like zinc finger (Pf01754) (CD superfamily: cl22285)
Ubiquitin interaction motif (Pf02809) (CD superfamily: cl03724)
VHS domain (Pf00790, Cl0009) (CD: cd03569, CD superfamily: cl02544)
Ubiquitin interaction motif (Pf02809)
GAT domain (Pf03127)
Hepatocyte growth factor-regulated tyrosine kinase substrate (Pf12210) (CD superfamily: cl13621)
C2H2 finger (in structure, annotated by UniProt)
RUN domain (Pf02759) (CD superfamily: cl02689)
Domain of unknown function (DUF3480) (Pf11979) (CD superfamily: cl13422)
FYVE zinc finger (CD: cd00065, CD superfamily: cl17764)
PDZ domain (Also known as DHR or GLGF) (Pf00595, Cl0466) (CD:cd00992, CD superfamily: cl00117)
Rab effector MyRIP/melanophilin C-terminus (Pf04698) (CD superfamily: cl12299)
FYVE-type with an inserted zinc binding loop and preceded by zinc binding loop (in structure, not annotated anywhere)
PBZ zinc ifnger (uniprot)
ADDz domain of ATRX (CD: cd11726/cd11727/cd11729, CD superfamily: cl17040) (Treble clef domain followed by PHD-like zinc finger)
SNF2 family N-terminal domain (Pf00176, Cl0023) (CD: cd00046, CD superfamily: cl21455)
PWWP domain (Pf00855, Cl0049) (CD: cd05837/cd05835/cd05839/cd05841, CD superfamily: cl02554)
C-5 cytosine-specific DNA methylase (Pf00145, Cl0063) (COG0270) (CD superfamily: cl21501)
ATP-utilising chromatin assembly and remodelling N-terminal (Pf10537) (CD superfamily: cl11169)
domain in different transcription and chromosome remodeling factors (smart00571) (CD superfamily: cl02674)
WSTF, HB1, Itc1p, MBD9 motif 1 (Pf15612) (CD superfamily: cl21411)
WSTF, HB1, Itc1p, MBD9 motif 2 (Pf15613) (CD superfamily: cl21412)
WSTF, HB1, Itc1p, MBD9 motif 3 (Pf15614) (CD superfamily: cl21413)
Domain of unknown function (DUF3594) (Pf12165) (CD superfamily: cl13583)
Sp100 domain (Pf03172) (CD superfamily: cl03917)
CHDNT (NUC034) domain (Pf08073, Cl0114) (CD superfamily: cl06902)
Chromo (CHRromatin Organisation MOdifier) domain (Pf00385, Cl0049) (CD: cd00024, CD superfamily: cl17459)
Domain of Unknown Function (DUF1087) (Pf06465) (CD superfamily: cl05792)
Domain of Unknown Function (DUF10876 (Pf06461) (CD superfamily: cl05788)
CHDCT2 (NUC038) domain (Pf08074) (CD superfamily: cl06903)
Transcription factor S-II (TFIIS), central domain (Pf07500) (smart00510) (CD superfamily: cl02637)
SPOC domain (Pf07744) (CD superfamily: cl06671)
N-terminal domain of DPF2/REQ. (Pf14051) (CD superfamily: cl16570)
Inhibitor of growth proteins N-terminal histone-binding (Pf12998) (CD superfamily: cl15229)
jmjN domain (Pf02375) (smart00545) (CD superfamily: cl15840)
ARID/BRIGHT DNA binding domain (Pf01388) (CD superfamily: cl02633)
JmjC domain, hydroxylase (Pf02373, Cl0029) (CD superfamily: cl19305)
Cupin-like domain (Pf13621, Cl0029) (smart00558) (CD superfamily: cl19305)
C5HC2 zinc finger (Pf02928) (CD superfamily: cl03786)
PLU-1-like protein (Pf08429)
CXXC zinc finger domain (Pf02008) (CD superfamily: cl03401)
PHD-like zinc-binding domain (Pf13771, Cl0390) (CD superfamily: cl17040)
Treble Clef domain (present in structure, annotated as "C2HC-type; pre-PHD" by UniProt)
F/Y-rich N-terminus (Pf05964)
F/Y rich C-terminus (Pf05965)
SET domain (Pf00856) (smart00317) (CD superfamily: cl02566)
Cysteine-rich motif following a subset of SET domains (smart00508) (CD superfamily: cl02636)
HMGB-UBF_HMG-box (CD: cd01390, CD superfamily: cl00082)
Prefoldin subunit (Pf01920) (CD superfamily: cl09111)
Tudor domain (smart00333) (CD superfamily: cl02573)
Polycomb-like MTF2 factor 2 (Pf14061) (CD superfamily: cl16580)
AT_hook (PF02178)

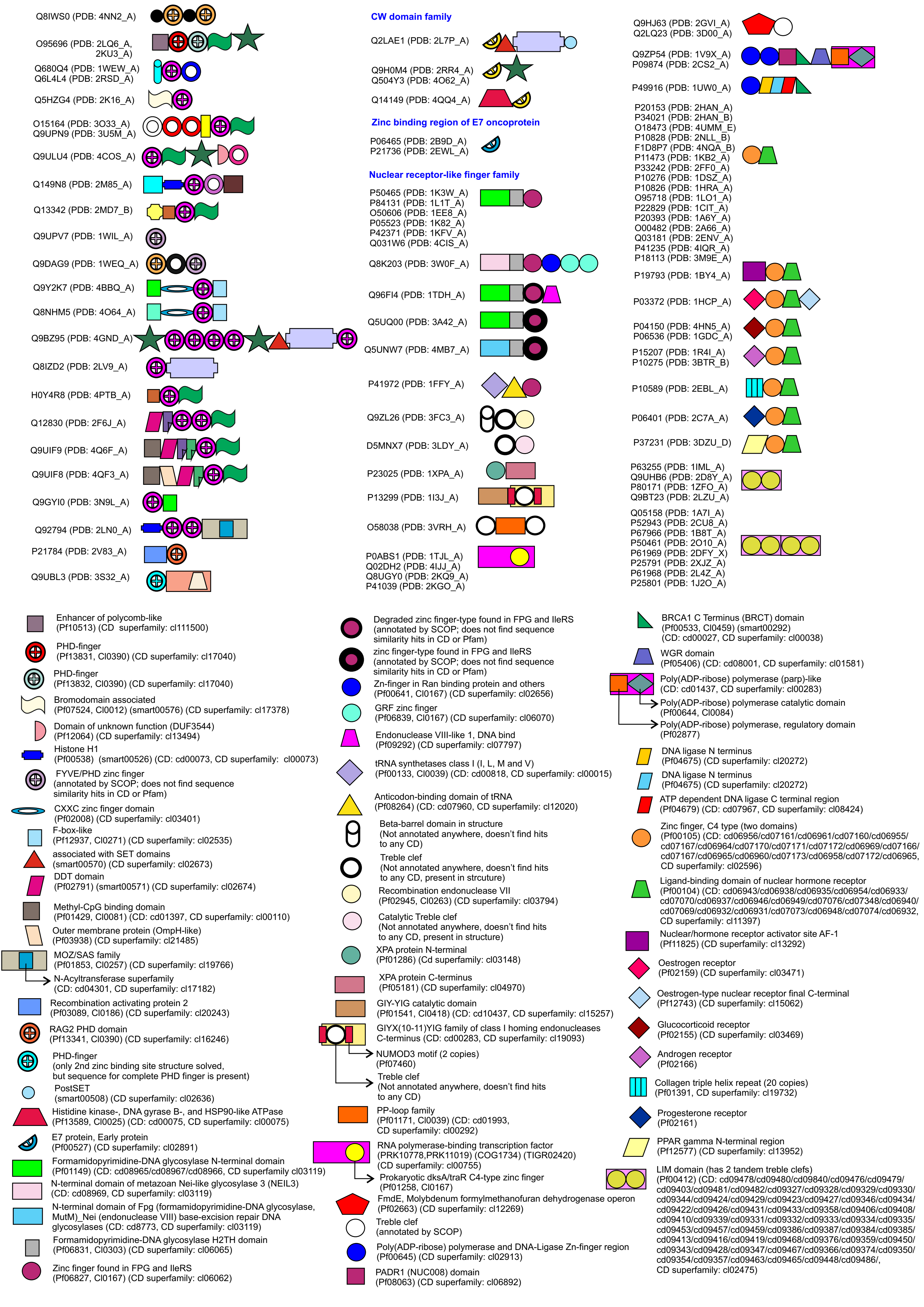
Q8IWS0 (PDB: 4NN2_A)
O95696 (PDB: 2LQ6_A, 2KU3_A)
Q680Q4 (PDB: 1WEW_A)
Q6L4L4 (PDB: 2RSD_A)
Q5HZG4 (PDB: 2K16_A)
O15164 (PDB: 3O33_A)
Q9UPN9 (PDB: 3U5M_A)
Q9ULU4 (PDB: 4COS_A)
Q149N8 (PDB: 2M85_A)
Q13342 (PDB: 2M7D_B)
Q9UPV7 (PDB: 1WIL_A)
Q9DAG9 (PDB: 1WEQ_A)
Q9Y2K7 (PDB: 4BBQ_A)
Q8NHM5 (PDB: 4O64_A)
Q9BZ95 (PDB: 4GND_A)
Q8IZD2 (PDB: 2LV9_A)
H0Y4R8 (PDB: 4PTB_A)
Q12830 (PDB: 2F6J_A)
Q9UIF9 (PDB: 4Q6F_A)
Q9UIF8 (PDB: 4QF3_A)
Q9GYI0 (PDB: 3N9L_A)
Q92794 (PDB: 2LN0_A)
P21784 (PDB: 2V83_A)
Q9UBL3 (PDB: 3S32_A)
CW domain family
Q2LAE1 (PDB: 2L7P_A)
Q9H0M4 (PDB: 2RR4_A)
Q504Y3 (PDB: 4O62_A)
Q14149 (PDB: 4QQ4_A)
Zinc binding region of E7 oncoprotein
P06465 (PDB: 2B9D_A)
P21736 (PDB: 2EWL_A)
Nuclear receptor-like finger family
P50465 (PDB: 1K3W_A)
P84131 (PDB: 1L1T_A)
O50606 (PDB: 1EE8_A)
P05523 (PDB: 1K82_A)
P42371 (PDB: 1KFV_A)
Q031W6 (PDB: 4CIS_A)
Q8K203 (PDB: 3W0F_A)
Q96FI4 (PDB: 1TDH_A)
Q5UQ00 (PDB: 3A42_A)
Q5UNW7 (PDB: 4MB7_A)
P41972 (PDB: 1FFY_A)
Q9ZL26 (PDB: 3FC3_A)
D5MNX7 (PDB: 3LDY_A)
P23025 (PDB: 1XPA_A)
P13299 (PDB: 1I3J_A)
O58038 (PDB: 3VRH_A)
P0ABS1 (PDB: 1TJL_A)
Q02DH2 (PDB: 4IJJ_A)
Q8UGY0 (PDB: 2KQ9_A)
P41039 (PDB: 2KGO_A)
Q9HJ63 (PDB: 2GVI_A)
Q2LQ23 (PDB: 3D00_A)
Q9ZP54 (PDB: 1V9X_A)
P09874 (PDB: 2CS2_A)
P49916 (PDB: 1UW0_A)
P20153 (PDB: 2HAN_A)
P34021 (PDB: 2HAN_B)
O18473 (PDB: 4UMM_E)
P10828 (PDB: 2NLL_B)
F1D8P7 (PDB: 4NQA_B)
P11473 (PDB: 1KB2_A)
P33242 (PDB: 2FF0_A)
P10276 (PDB: 1DSZ_A)
P10826 (PDB: 1HRA_A)
O95718 (PDB: 1LO1_A)
P22829 (PDB: 1CIT_A)
P20393 (PDB: 1A6Y_A)
O00482 (PDB: 2A66_A)
Q03181 (PDB: 2ENV_A)
P41235 (PDB: 4IQR_A)
P18113 (PDB: 3M9E_A)
P19793 (PDB: 1BY4_A)
P03372 (PDB: 1HCP_A)
P04150 (PDB: 4HN5_A)
P06536 (PDB: 1GDC_A)
P15207 (PDB: 1R4I_A)
P10275 (PDB: 3BTR_B)
P10589 (PDB: 2EBL_A)
P06401 (PDB: 2C7A_A)
P37231 (PDB: 3DZU_D)
P63255 (PDB: 1IML_A)
Q9UHB6 (PDB: 2D8Y_A)
P80171 (PDB: 1ZFO_A)
Q9BT23 (PDB: 2LZU_A)
Q05158 (PDB: 1A7I_A)
P52943 (PDB: 2CU8_A)
P67966 (PDB: 1B8T_A)
P50461 (PDB: 2O10_A)
P61969 (PDB: 2DFY_X)
P25791 (PDB: 2XJZ_A)
P61968 (PDB: 2L4Z_A)
P25801 (PDB: 1J2O_A)
Enhancer of polycomb-like (Pf10513) (CD superfamily: cl111500)
PHD-finger (Pf13831, Cl0390) (CD superfamily: cl17040)
PHD-finger (Pf13832, Cl0390) (CD superfamily: cl17040)
Bromodomain associated (Pf07524, Cl0012) (smart00576) (CD superfamily: cl17378)
Domain of unknown function (DUF3544) (Pf12064) (CD superfamily: cl13494)
Histone H1 (Pf00538) (smart00526) (CD: cd00073, CD superfamily: cl00073)
FYVE/PHD zinc finger (annotated by SCOP; does not find sequence similarity hits in CD or Pfam)
CXXC zinc finger domain (Pf02008) (CD superfamily: cl03401)
F-box-like (Pf12937, Cl0271) (CD superfamily: cl02535)
associated with SET domains (smart00570) (CD superfamily: cl02673)
DDT domain (Pf02791) (smart00571) (CD superfamily: cl02674)
Methyl-CpG binding domain (Pf01429, Cl0081) (CD: cd01397, CD superfamily: cl00110)
Outer membrane protein (OmpH-like) (Pf03938) (CD superfamily: cl21485)
MOZ/SAS family (Pf01853, Cl0257) (CD superfamily: cl19766)
N-Acyltransferase superfamily (CD: cd04301, CD superfamily: cl17182)
Recombination activating protein 2 (Pf03089, Cl0186) (CD superfamily: cl20243)
RAG2 PHD domain (Pf13341, Cl0390) (CD superfamily: cl16246)
PHD-finger (only 2nd zinc binding site structure solved, but sequence for complete PHD finger is present)
PostSET (smart00508) (CD superfamily: cl02636)
Histidine kinase-, DNA gyrase B-, and HSP90-like ATPase (Pf13589, Cl0025) (CD: cd00075, CD superfamily: cl00075)
E7 protein, Early protein (Pf00527) (CD superfamily: cl02891)
Formamidopyrimidine-DNA glycosylase N-terminal domain (Pf01149) (CD: cd08965/cd08967/cd08966, CD superfamily cl03119)
N-terminal domain of metazoan Nei-like glycosylase 3 (NEIL3) (CD: cd08969, CD superfamily: cl03119)
N-terminal domain of Fpg (formamidopyrimidine-DNA glycosylase, MutM)_Nei (endonuclease VIII) base-excision repair DNA glycosylases (CD: cd8773, CD superfamily: cl03119)
Formamidopyrimidine-DNA glycosylase H2TH domain (Pf06831, Cl0303) (CD superfamily: cl06065)
Zinc finger found in FPG and IleRS (Pf06827, Cl0167) (CD superfamily: cl06062)
Degraded zinc finger-type found in FPG and IleRS (annotated by SCOP; does not find sequence similarity hits in CD or Pfam)
zinc finger-type found in FPG and IleRS (annotated by SCOP; does not find sequence similarity hits in CD or Pfam)
Zn-finger in Ran binding protein and others (Pf00641, Cl0167) (CD superfamily: cl02656)
GRF zinc finger (Pf06839, Cl0167) (CD superfamily: cl06070)
Endonuclease VIII-like 1, DNA bind (Pf09292) (CD superfamily: cl07797)
tRNA synthetases class I (I, L, M and V) (Pf00133, Cl0039) (CD: cd00818, CD superfamily: cl00015)
Anticodon-binding domain of tRNA (Pf08264) (CD: cd07960, CD superfamily: cl12020)
Beta-barrel domain in structure (Not annotated anywhere, doesn't find hits to any CD)
Treble clef (Not annotated anywhere, doesn't find hits to any CD, present in strcuture)
Recombination endonuclease VII (Pf02945, Cl0263) (CD superfamily: cl03794)
Catalytic Treble clef (Not annotated anywhere, doesn't find hits to any CD, present in structure)
XPA protein N-terminal (Pf01286) (Cd superfamily: cl03148)
XPA protein C-terminus (Pf05181) (CD superfamily: cl04970)
GIY-YIG catalytic domain (Pf01541, Cl0418) (CD: cd10437, CD superfamily: cl15257)
GIYX(10-11)YIG family of class I homing endonucleases C-terminus (CD: cd00283, CD superfamily: cl19093)
NUMOD3 motif (2 copies) (Pf07460)
Treble clef (Not annotated anywhere, doesn't find hits to any CD)
PP-loop family (Pf01171, Cl0039) (CD: cd01993, CD superfamily: cl00292)
RNA polymerase-binding transcription factor (PRK10778,PRK11019) (COG1734) (TIGR02420) (CD superfamily: cl00755)
Prokaryotic dksA/traR C4-type zinc finger (Pf01258, Cl0167)
FmdE, Molybdenum formylmethanofuran dehydrogenase operon (Pf02663) (CD superfamily: cl12269)
Treble clef (annotated by SCOP)
Poly(ADP-ribose) polymerase and DNA-Ligase Zn-finger region (Pf00645) (CD superfamily: cl02913)
PADR1 (NUC008) domain (Pf08063) (CD superfamily: cl06892)
BRCA1 C Terminus (BRCT) domain (Pf00533, Cl0459) (smart00292) (CD: cd00027, CD superfamily: cl00038)
WGR domain (Pf05406) (CD: cd08001, CD superfamily: cl01581)
Poly(ADP-ribose) polymerase (parp)-like (CD: cd01437, CD superfamily: cl00283)
Poly(ADP-ribose) polymerase catalytic domain (Pf00644, Cl0084)
Poly(ADP-ribose) polymerase, regulatory domain (Pf02877)
DNA ligase N terminus (Pf04675) (CD superfamily: cl20272)
DNA ligase N terminus (Pf04675) (CD superfamily: cl20272)
ATP dependent DNA ligase C terminal region (Pf04679) (CD: cd07967, CD superfamily: cl08424)
Zinc finger, C4 type (two domains) (Pf00105) (CD: cd06956/cd07161/cd06961/cd07160/cd06955/ cd07167/cd06964/cd07170/cd07171/cd07172/cd06969/cd07166/ cd07167/cd06965/cd06960/cd07173/cd06958/cd07172/cd06965, CD superfamily: cl02596)
Ligand-binding domain of nuclear hormone receptor (Pf00104) (CD: cd06943/cd06938/cd06935/cd06954/cd06933/ cd07070/cd06937/cd06946/cd06949/cd07076/cd07348/cd06940/ cd07069/cd06932/cd06931/cd07073/cd06948/cd07074/cd06932, CD superfamily: cl11397)
Nuclear/hormone receptor activator site AF-1 (Pf11825) (CD superfamily: cl13292)
Oestrogen receptor (Pf02159) (CD superfamily: cl03471)
Oestrogen-type nuclear receptor final C-terminal (Pf12743) (CD superfamily: cl15062)
Glucocorticoid receptor (Pf02155) (CD superfamily: cl03469)
Androgen receptor (Pf02166)
Collagen triple helix repeat (20 copies) (Pf01391, CD superfamily: cl19732)
Progesterone receptor (Pf02161)
PPAR gamma N-terminal region (Pf12577) (CD superfamily: cl13952)
LIM domain (has 2 tandem treble clefs) (Pf00412) (CD: cd09478/cd09480/cd09840/cd09476/cd09479/ cd09403/cd09481/cd09482/cd09327/cd09328/cd09329/cd09330/ cd09344/cd09424/cd09429/cd09423/cd09427/cd09346/cd09434/ cd09422/cd09426/cd09431/cd09433/cd09358/cd09406/cd09408/ cd09410/cd09339/cd09331/cd09332/cd09333/cd09334/cd09335/ cd09453/cd09457/cd09459/cd09386/cd09387/cd09384/cd09385/ cd09413/cd09416/cd09419/cd09468/cd09376/cd09359/cd09450/ cd09343/cd09428/cd09347/cd09467/cd09366/cd09374/cd09350/ cd09354/cd09357/cd09463/cd09465/cd09448/cd09486/, CD superfamily: cl02475)

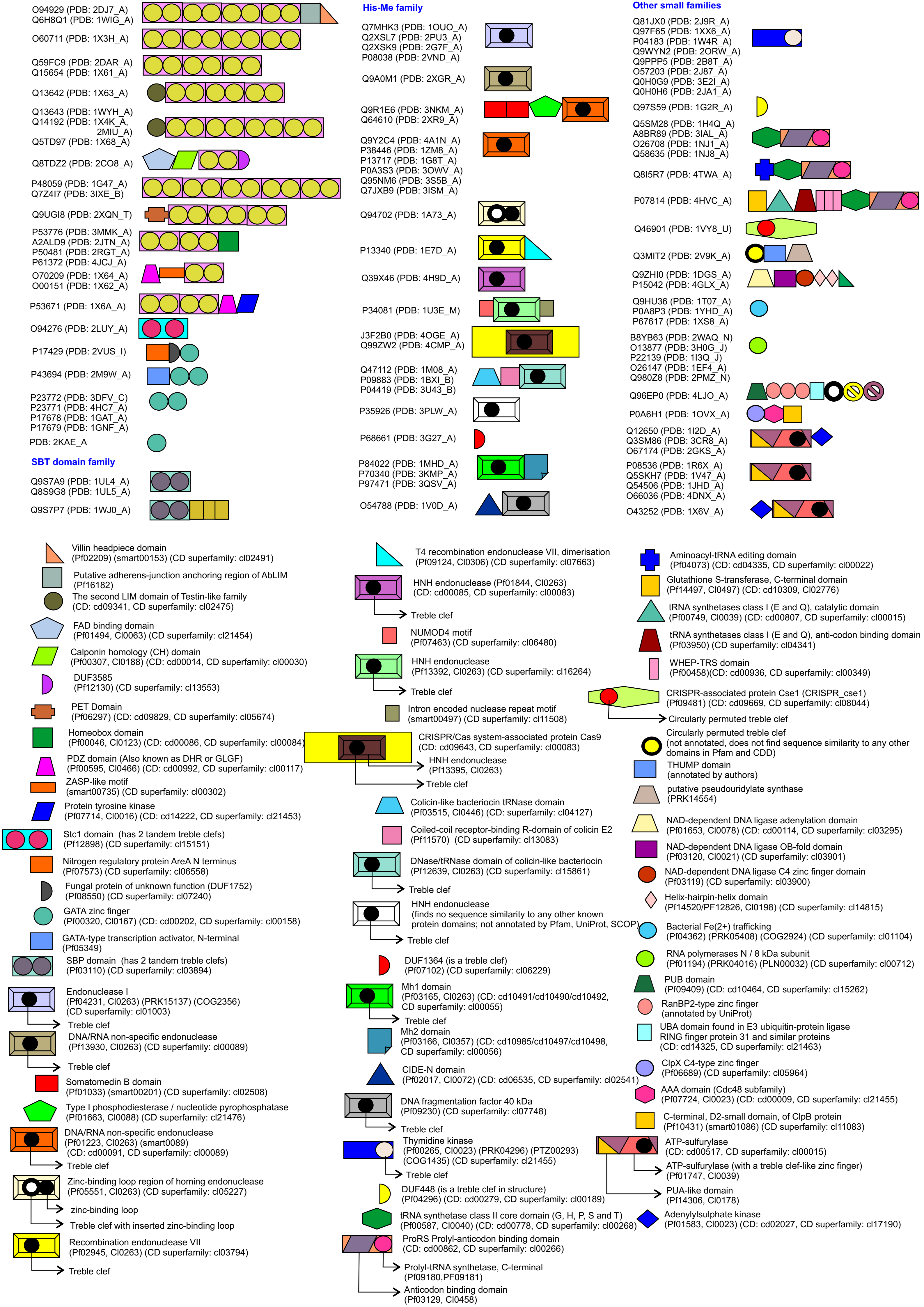
O94929 (PDB: 2DJ7_A)
Q6H8Q1 (PDB: 1WIG_A)
O60711 (PDB: 1X3H_A)
Q59FC9 (PDB: 2DAR_A)
Q15654 (PDB: 1X61_A)
Q13642 (PDB: 1X63_A)
Q13643 (PDB: 1WYH_A)
Q14192 (PDB: 1X4K_A,
2MIU_A)
Q5TD97 (PDB: 1X68_A)
Q8TDZ2 (PDB: 2CO8_A)
P48059 (PDB: 1G47_A)
Q7Z4I7 (PDB: 3IXE_B)
Q9UGI8 (PDB: 2XQN_T)
P53776 (PDB: 3MMK_A)
A2ALD9 (PDB: 2JTN_A)
P50481 (PDB: 2RGT_A)
P61372 (PDB: 4JCJ_A)
O70209 (PDB: 1X64_A)
O00151 (PDB: 1X62_A)
P53671 (PDB: 1X6A_A)
O94276 (PDB: 2LUY_A)
P17429 (PDB: 2VUS_I)
P43694 (PDB: 2M9W_A)
P23772 (PDB: 3DFV_C)
P23771 (PDB: 4HC7_A)
P17678 (PDB: 1GAT_A)
P17679 (PDB: 1GNF_A)
PDB: 2KAE_A
SBT domain family
Q9S7A9 (PDB: 1UL4_A)
Q8S9G8 (PDB: 1UL5_A)
Q9S7P7 (PDB: 1WJ0_A)
His-Me family
Q7MHK3 (PDB: 1OUO_A)
Q2XSL7 (PDB: 2PU3_A)
Q2XSK9 (PDB: 2G7F_A)
P08038 (PDB: 2VND_A)
Q9A0M1 (PDB: 2XGR_A)
Q9R1E6 (PDB: 3NKM_A)
Q64610 (PDB: 2XR9_A)
Q9Y2C4 (PDB: 4A1N_A)
P38446 (PDB: 1ZM8_A)
P13717 (PDB: 1G8T_A)
P0A3S3 (PDB: 3OWV_A)
Q95NM6 (PDB: 3S5B_A)
Q7JXB9 (PDB: 3ISM_A)
Q94702 (PDB: 1A73_A)
P13340 (PDB: 1E7D_A)
Q39X46 (PDB: 4H9D_A)
P34081 (PDB: 1U3E_M)
J3F2B0 (PDB: 4OGE_A)
Q99ZW2 (PDB: 4CMP_A)
Q47112 (PDB: 1M08_A)
P09883 (PDB: 1BXI_B)
P04419 (PDB: 3U43_B)
P35926 (PDB: 3PLW_A)
P68661 (PDB: 3G27_A)
P84022 (PDB: 1MHD_A)
P70340 (PDB: 3KMP_A)
P97471 (PDB: 3QSV_A)
O54788 (PDB: 1V0D_A)
Other small families
Q81JX0 (PDB: 2J9R_A)
Q97F65 (PDB: 1XX6_A)
P04183 (PDB: 1W4R_A)
Q9WYN2 (PDB: 2ORW_A)
Q9PPP5 (PDB: 2B8T_A)
O57203 (PDB: 2J87_A)
Q0H0G9 (PDB: 3E2I_A)
Q0H0H6 (PDB: 2JA1_A)
Q97S59 (PDB: 1G2R_A)
Q5SM28 (PDB: 1H4Q_A)
A8BR89 (PDB: 3IAL_A)
O26708 (PDB: 1NJ1_A)
Q58635 (PDB: 1NJ8_A)
Q8I5R7 (PDB: 4TWA_A)
P07814 (PDB: 4HVC_A)
Q46901 (PDB: 1VY8_U)
Q3MIT2 (PDB: 2V9K_A)
Q9ZHI0 (PDB: 1DGS_A)
P15042 (PDB: 4GLX_A)
Q9HU36 (PDB: 1T07_A)
P0A8P3 (PDB: 1YHD_A)
P67617 (PDB: 1XS8_A)
B8YB63 (PDB: 2WAQ_N)
O13877 (PDB: 3H0G_J)
P22139 (PDB: 1I3Q_J)
O26147 (PDB: 1EF4_A)
Q980Z8 (PDB: 2PMZ_N)
Q96EP0 (PDB: 4LJO_A)
P0A6H1 (PDB: 1OVX_A)
Q12650 (PDB: 1I2D_A)
Q3SM86 (PDB: 3CR8_A)
O67174 (PDB: 2GKS_A)
P08536 (PDB: 1R6X_A)
Q5SKH7 (PDB: 1V47_A)
Q54506 (PDB: 1JHD_A)
O66036 (PDB: 4DNX_A)
O43252 (PDB: 1X6V_A)
Villin headpiece domain
(Pf02209) (smart00153) (CD superfamily: cl02491)
Putative adherens-junction anchoring region of AbLIM
(Pf16182)
The second LIM domain of Testin-like family
(CD: cd09341, CD superfamily: cl02475)
FAD binding domain
(Pf01494, Cl0063) (CD superfamily: cl21454)
Calponin homology (CH) domain
(Pf00307, Cl0188) (CD: cd00014, CD superfamily: cl00030)
DUF3585
(Pf12130) (CD superfamily: cl13553)
PET Domain
(Pf06297) (CD: cd09829, CD superfamily: cl05674)
Homeobox domain
(Pf00046, Cl0123) (CD: cd00086, CD superfamily: cl00084)
PDZ domain (Also known as DHR or GLGF)
(Pf00595, Cl0466) (CD: cd00992, CD superfamily: cl00117)
ZASP-like motif
(smart00735) (CD superfamily: cl00302)
Protein tyrosine kinase
(Pf07714, Cl0016) (CD: cd14222, CD superfamily: cl21453)
Stc1 domain (has 2 tandem treble clefs)
(Pf12898) (CD superfamily: cl15151)
Nitrogen regulatory protein AreA N terminus
(Pf07573) (CD superfamily: cl06558)
Fungal protein of unknown function (DUF1752)
(Pf08550) (CD superfamily: cl07240)
GATA zinc finger
(Pf00320, Cl0167) (CD: cd00202, CD superfamily: cl00158)
GATA-type transcription activator, N-terminal
(Pf05349)
SBP domain (has 2 tandem treble clefs)
(Pf03110) (CD superfamily: cl03894)
Endonuclease I
(Pf04231, Cl0263) (PRK15137) (COG2356)
(CD superfamily: cl01003)
Treble clef
DNA/RNA non-specific endonuclease
(Pf13930, Cl0263) (CD superfamily: cl00089)
Treble clef
Somatomedin B domain
(Pf01033) (smart00201) (CD superfamily: cl02508)
Type I phosphodiesterase / nucleotide pyrophosphatase
(Pf01663, Cl0088) (CD superfamily: cl21476)
DNA/RNA non-specific endonuclease
(Pf01223, Cl0263) (smart0089)
(CD: cd00091, CD superfamily: cl00089)
Treble clef
Zinc-binding loop region of homing endonuclease
(Pf05551, Cl0263) (CD superfamily: cl05227)
zinc-binding loop
Treble clef with inserted zinc-binding loop
Recombination endonuclease VII
(Pf02945, Cl0263) (CD superfamily: cl03794)
Treble clef
T4 recombination endonuclease VII, dimerisation
(Pf09124, Cl0306) (CD superfamily: cl07663)
HNH endonuclease (Pf01844, Cl0263)
(CD: cd00085, CD superfamily: cl00083)
Treble clef
NUMOD4 motif
(Pf07463) (CD superfamily: cl06480)
HNH endonuclease
(Pf13392, Cl0263) (CD superfamily: cl16264)
Treble clef
Intron encoded nuclease repeat motif
(smart00497) (CD superfamily: cl11508)
CRISPR/Cas system-associated protein Cas9
(CD: cd09643, CD superfamily: cl00083)
HNH endonuclease
(Pf13395, Cl0263)
Treble clef
Colicin-like bacteriocin tRNase domain
(Pf03515, Cl0446) (CD: superfamily: cl04127)
Coiled-coil receptor-binding R-domain of colicin E2
(Pf11570) (CD superfamily: cl13083)
DNase/tRNase domain of colicin-like bacteriocin
(Pf12639, Cl0263) (CD superfamily: cl15861)
Treble clef
HNH endonuclease
(finds no sequence similarity to any other known
protein domains; not annotated by Pfam, UniProt, SCOP)
Treble clef
DUF1364 (is a treble clef)
(Pf07102) (CD superfamily: cl06229)
Mh1 domain
(Pf03165, Cl0263) (CD: cd10491/cd10490/cd10492,
CD superfamily: cl00055)
Treble clef
Mh2 domain
(Pf03166, Cl0357) (CD: cd10985/cd10497/cd10498,
CD superfamily: cl00056)
CIDE-N domain
(Pf02017, Cl0072) (CD: cd06535, CD superfamily: cl02541)
DNA fragmentation factor 40 kDa
(Pf09230) (CD superfamily: cl07748)
Treble clef
Thymidine kinase
(Pf00265, Cl0023) (PRK04296) (PTZ00293)
(COG1435) (CD superfamily: cl21455)
Treble clef
DUF448 (is a treble clef in structure)
(Pf04296) (CD: cd00279, CD superfamily: cl00189)
tRNA synthetase class II core domain (G, H, P, S and T)
(Pf00587, Cl0040) (CD: cd00778, CD superfamily: cl00268)
ProRS Prolyl-anticodon binding domain
(CD: cd00862, CD superfamily: cl00266)
Prolyl-tRNA synthetase, C-terminal
(Pf09180,PF09181)
Anticodon binding domain
(Pf03129, Cl0458)
Aminoacyl-tRNA editing domain
(Pf04073) (CD: cd04335, CD superfamily: cl00022)
Glutathione S-transferase, C-terminal domain
(Pf14497, Cl0497) (CD: cd10309, Cl02776)
tRNA synthetases class I (E and Q), catalytic domain
(Pf00749, Cl0039) (CD: cd00807, CD superfamily: cl00015)
tRNA synthetases class I (E and Q), anti-codon binding domain
(Pf03950) (CD superfamily: cl04341)
WHEP-TRS domain
(Pf00458)(CD: cd00936, CD superfamily: cl00349)
CRISPR-associated protein Cse1 (CRISPR_cse1)
(Pf09481) (CD: cd09669, CD superfamily: cl08044)
Circularly permuted treble clef
Circularly permuted treble clef
(not annotated, does not find sequence similarity to any other
domains in Pfam and CDD)
THUMP domain
(annotated by authors)
putative pseudouridylate synthase
(PRK14554)
NAD-dependent DNA ligase adenylation domain
(Pf01653, Cl0078) (CD: cd00114, CD superfamily: cl03295)
NAD-dependent DNA ligase OB-fold domain
(Pf03120, Cl0021) (CD superfamily: cl03901)
NAD-dependent DNA ligase C4 zinc finger domain
(Pf03119) (CD superfamily: cl03900)
Helix-hairpin-helix domain
(Pf14520/PF12826, Cl0198) (CD superfamily: cl14815)
Bacterial Fe(2+) trafficking
(Pf04362) (PRK05408) (COG2924) (CD superfamily: cl01104)
RNA polymerases N / 8 kDa subunit
(Pf01194) (PRK04016) (PLN00032) (CD superfamily: cl00712)
PUB domain
(Pf09409) (CD: cd10464, CD superfamily: cl15262)
RanBP2-type zinc finger
(annotated by UniProt)
UBA domain found in E3 ubiquitin-protein ligase
RING finger protein 31 and similar proteins
(CD: cd14325, CD superfamily: cl21463)
ClpX C4-type zinc finger
(Pf06689) (CD superfamily: cl05964)
AAA domain (Cdc48 subfamily)
(Pf07724, Cl0023) (CD: cd00009, CD superfamily: cl21455)
C-terminal, D2-small domain, of ClpB protein
(Pf10431) (smart01086) (CD superfamily: cl11083)
ATP-sulfurylase
(CD: cd00517, CD superfamily: cl00015)
ATP-sulfurylase (with a treble clef-like zinc finger)
(Pf01747, Cl0039)
PUA-like domain
(Pf14306, Cl0178)
Adenylylsulphate kinase
(Pf01583, Cl0023) (CD: cd02027, CD superfamily: cl17190)

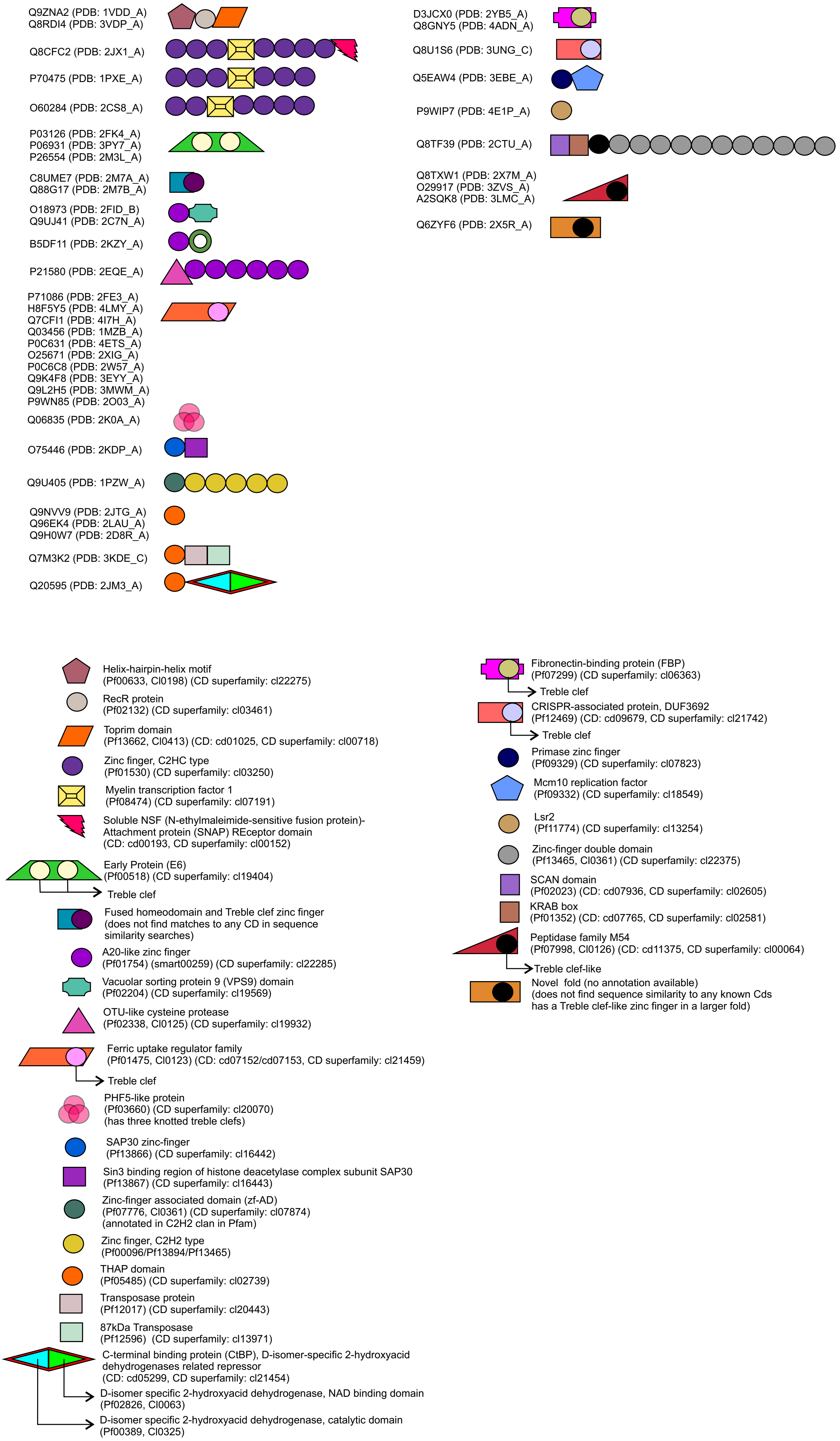
Q9ZNA2 (PDB: 1VDD_A)
Q8RDI4 (PDB: 3VDP_A)
Q8CFC2 (PDB: 2JX1_A)
P70475 (PDB: 1PXE_A)
O60284 (PDB: 2CS8_A)
P03126 (PDB: 2FK4_A)
P06931 (PDB: 3PY7_A)
P26554 (PDB: 2M3L_A)
C8UME7 (PDB: 2M7A_A)
Q88G17 (PDB: 2M7B_A)
O18973 (PDB: 2FID_B)
Q9UJ41 (PDB: 2C7N_A)
B5DF11 (PDB: 2KZY_A)
P21580 (PDB: 2EQE_A)
P71086 (PDB: 2FE3_A)
H8F5Y5 (PDB: 4LMY_A)
Q7CFI1 (PDB: 4I7H_A)
Q03456 (PDB: 1MZB_A)
P0C631 (PDB: 4ETS_A)
O25671 (PDB: 2XIG_A)
P0C6C8 (PDB: 2W57_A)
Q9K4F8 (PDB: 3EYY_A)
Q9L2H5 (PDB: 3MWM_A)
P9WN85 (PDB: 2O03_A)
Q06835 (PDB: 2K0A_A)
O75446 (PDB: 2KDP_A)
Q9U405 (PDB: 1PZW_A)
Q9NVV9 (PDB: 2JTG_A)
Q96EK4 (PDB: 2LAU_A)
Q9H0W7 (PDB: 2D8R_A)
Q7M3K2 (PDB: 3KDE_C)
Q20595 (PDB: 2JM3_A)
D3JCX0 (PDB: 2YB5_A)
Q8GNY5 (PDB: 4ADN_A)
Q8U1S6 (PDB: 3UNG_C)
Q5EAW4 (PDB: 3EBE_A)
P9WIP7 (PDB: 4E1P_A)
Q8TF39 (PDB: 2CTU_A)
Q8TXW1 (PDB: 2X7M_A)
O29917 (PDB: 3ZVS_A)
A2SQK8 (PDB: 3LMC_A)
Q6ZYF6 (PDB: 2X5R_A)
Helix-hairpin-helix motif
(Pf00633, Cl0198) (CD superfamily: cl22275)
RecR protein
(Pf02132) (CD superfamily: cl03461)
Toprim domain
(Pf13662, Cl0413) (CD: cd01025, CD superfamily: cl00718)
Zinc finger, C2HC type
(Pf01530) (CD superfamily: cl03250)
Myelin transcription factor 1
(Pf08474) (CD superfamily: cl07191)
Soluble NSF (N-ethylmaleimide-sensitive fusion protein)-
Attachment protein (SNAP) REceptor domain
(CD: cd00193, CD superfamily: cl00152)
Early Protein (E6)
(Pf00518) (CD superfamily: cl19404)
Treble clef
Fused homeodomain and Treble clef zinc finger
(does not find matches to any CD in sequence
similarity searches)
A20-like zinc finger
(Pf01754) (smart00259) (CD superfamily: cl22285)
Vacuolar sorting protein 9 (VPS9) domain
(Pf02204) (CD superfamily: cl19569)
OTU-like cysteine protease
(Pf02338, Cl0125) (CD superfamily: cl19932)
Ferric uptake regulator family
(Pf01475, Cl0123) (CD: cd07152/cd07153, CD superfamily: cl21459)
Treble clef
PHF5-like protein
(Pf03660) (CD superfamily: cl20070)
(has three knotted treble clefs)
SAP30 zinc-finger
(Pf13866) (CD superfamily: cl16442)
Sin3 binding region of histone deacetylase complex subunit SAP30
(Pf13867) (CD superfamily: cl16443)
Zinc-finger associated domain (zf-AD)
(Pf07776, Cl0361) (CD superfamily: cl07874)
(annotated in C2H2 clan in Pfam)
Zinc finger, C2H2 type
(Pf00096/Pf13894/Pf13465)
THAP domain
(Pf05485) (CD superfamily: cl02739)
Transposase protein
(Pf12017) (CD superfamily: cl20443)
87kDa Transposase
(Pf12596) (CD superfamily: cl13971)
C-terminal binding protein (CtBP), D-isomer-specific 2-hydroxyacid
dehydrogenases related repressor
(CD: cd05299, CD superfamily: cl21454)
D-isomer specific 2-hydroxyacid dehydrogenase, NAD binding domain
(Pf02826, Cl0063)
D-isomer specific 2-hydroxyacid dehydrogenase, catalytic domain
(Pf00389, Cl0325)
Fibronectin-binding protein (FBP)
(Pf07299) (CD superfamily: cl06363)
Treble clef
CRISPR-associated protein, DUF3692
(Pf12469) (CD: cd09679, CD superfamily: cl21742)
Treble clef
Primase zinc finger
(Pf09329) (CD superfamily: cl07823)
Mcm10 replication factor
(Pf09332) (CD superfamily: cl18549)
Lsr2
(Pf11774) (CD superfamily: cl13254)
Zinc-finger double domain
(Pf13465, Cl0361) (CD superfamily: cl22375)
SCAN domain
(Pf02023) (CD: cd07936, CD superfamily: cl02605)
KRAB box
(Pf01352) (CD: cd07765, CD superfamily: cl02581)
Peptidase family M54
(Pf07998, Cl0126) (CD: cd11375, CD superfamily: cl00064)
Treble clef-like
Novel fold (no annotation available)
(does not find sequence similarity to any known Cds
has a Treble clef-like zinc finger in a larger fold)
