# Supplementary material for: Classification of the treble clef zinc finger: noteworthy lessons for structure and function evolution
Source: Sci Rep. 2016 Aug 26;6:32070. doi: 10.1038/srep32070 (PMC4999995; doi:10.1038/srep32070)
Supplement: Supplementary File 3 [file srep32070-s4.pdf]

**Title:**

# Classification of the treble clef zinc finger: noteworthy lessons for structure and function evolution

**Authors:**

Gurmeet Kaur, Srikrishna Subramanian[1]

**Contact Information**

CSIR-Institute of Microbial Technology (IMTECH), Sector 39-A, Chandigarh, 160036, India. Phone: +911726665483; Fax: +91-1722695215; Email: krishna@imtech.res.in

[1] Corresponding author

## Supplementary File 4: Methodology in detail

**Collection of all TC ZF structures**

The dataset of TC ZFs to be classified in the present study was generated using four approaches, all of which combined manual analysis with automated methods. First, the PDB identifier(s) (PDBids) of TC ZFs that were previously classified by Krishna et al., 2003 [1] constituted the initial dataset. SCOP v1.75 [2] and Pfam v27.0 [3] were searched with these PDBids. The PDBids of TC ZFs belonging to the same SCOP fold and same Pfam family and clan that were not present in the initial dataset were added. Additionally, the UniProt identifier linked to each ZF protein on the PDB webserver was searched. All the PDBids for the sequence corresponding to the ZF, but again, which were not already in the dataset, were added. The latter enabled to populate the dataset with all the structures of the same protein; some of which were released after the last stable release of Pfam (v27.0) and SCOP (v1.75) used in this study.

Second, the sequences and structures of non-identical TC ZFs (i.e. with unique UniProt identifiers) in the dataset were fetched from UniProt and PDB, respectively. If a UniProt identifier had more than one structures for the domain, then X-ray structure was preferably chosen over the nuclear magnetic resonance (NMR)/electron microscope (EM) structure; and of the many X-ray structures of a single ZF, the one with the best resolution was chosen. These were used to initiate sequence and structure similarity searches (see Methods below). The PDBids of the matches obtained after performing these searches were added to the dataset.

Third, the weekly release of new structures at the PDB (until 15 August 2014) was simultaneously scanned to fetch TC ZFs in the newly deposited structures, and the PDBids of these were added to the dataset.

Fourth, an automated approach was used to collect all the structures deposited in the PDB that had ‘ZN’ in the ‘HETATM’ record of the PDB structure files. For this, complete

PDB as on 1 May 2013 (90, 206 structures) was downloaded and stored locally. A Perl script was used to find zinc ions in PDB structure files and detect the presence of four metal-chelating residues in a sphere of 3 Å around it. The sequences of unique PDBids obtained after the step above were fetched from the PDB SeqRes records. Poly-nucleotide chains were removed from these sequences. After that, these protein sequences were clustered at 50 percent sequence identity (%ID) and 90 % coverage using cd-hit [4]. Clustering helped reduce the initial set of proteins, many of which were redundant, for example, identical proteins with more than one structures and homologous proteins with high sequence identity and thus, similar structure. The list of PDBids obtained after clustering was visualized in PyMOL and the ZF(s) in each was identified. The PDBids of the bonafide TC ZFs identified in this step were added to the initial dataset list (again, if it was not already present there). PDBids of the protein domains where the zinc ion did not form the core of the fold or others where zinc was a part of the catalytic site in another fold or was present as a consequence of crystallization or as a secondary site were excluded from the study.

**Classification of TC ZFs into families**

The TC ZFs identified in the above step were analysed in detail. The analysis included searching for sequentially and structurally similar proteins, literature survey for determining function and interacting partners, and studying co-occurring domains. For some interesting and novel TC ZFs discovered in this study detailed analysis for their distribution across the different life forms and evolutionary patterns seen for residues involved in zinc-chelation, gene-co-occurrence, etc. were specifically studied. Based on the information gathered, the TCs were grouped into families of homologous domains that are likely to be related in evolution. Evolutionary connections are implied within the members of the same family; however, different families may not be affirmatively related to each other, based on

the presently available protein sequence and structure data, and the tools that were used to assess similarities.

**Structure-based methods**

Structural similarity of TC ZFs in the dataset with each other and to other protein domains in the PDB was assessed using Dali [5], TM-align [6], Fr-TM-align [7] and TopSearch [8] protein structure similarity search tools. Dali [5] and TopSearch [8] were used to find structural matches of the query ZF structures in the PDB. The pairwise-Dali [5], TM-align [6] and Fr-TM-align [7] were used to assess automated pairwise structural similarity. The structures were visually compared and manually superimposed with each other by defining the equivalent regions using the pair fitting command of the molecular visualization program PyMOL.

**Sequence-based methods**

BLAST [9], PSI-BLAST [10] (against the regularly updated PDB and NR; E-value threshold of 0.001), Fold and Function Assignment (FFAS) [11] (against the regularly updated PDB, Pfam v27.0 and SCOP v1.75 databases), JackHMMER from the HMMER3 package [12] (against the regularly updated PDB and NR; E-value threshold of 0.001) and HHpred [13] (against the regularly updated PDB, SCOPe95_2.04 and PfamA_27.0 databases; using MSA generation method HHblits, run for 5 iterations; E-value threshold of 0.001) were used to detect sequence similarity among protein domains and establish sequence-based evolutionary connections. These methods were also used to find any new TC ZFs that were not already present in the dataset. Proteins sequences that matched with an E-value of 1E-5 or better, shared sequence similarity over the complete domain and possessed similar structures, were considered to be true homologs. The threshold E-values and cut-offs were relaxed when required, to allow detection of plausible remote homologs, with other supportive evidence

from structure and function comparison, the presence of conserved functional motifs, co-occurring domain patterns, etc.

For ZF-like sequences and structures retrieved during similarity searches, where the expected zinc-chelating residues were missing/substituted in the sequence and no zinc was bound in the structures, the sequences of homologous proteins in the NCBI NR database or UniProt were retrieved by running PSI-BLAST [10] and/or JackHMMER [12] and analysed thoroughly. To validate them as bonafide ZFs, the presence of zinc-binding residues at expected positions in the sequence as inferred from the available structures, was checked.

**Clustering of protein sequences**

Cd-hit [4] tool was used to perform a sequence-based clustering of the proteins to derive sets of representative sequences of various families that were used in the construction of multiple sequence alignments (MSAs). Cd-hit was also used in particular cases of novel TC ZFs to cluster homologous sequences to generate a representative set for building MSAs and to study other evolutionary patterns. The cut-off %ID for the sequences to be clustered was determined by taking into account the variability in each sequence set and decided empirically in each case. The sequence coverage was kept at 90% during all clustering steps.

**Multiple sequence alignment**

The MSAs of homologs of any particular TC ZF were created using the ClustalW program [14] within the BioEdit software package (version 7.2.2) [15] with default parameters. MSA for each family of the TC ZFs was prepared using PROMALS3D program [16] with default parameters. All the MSAs generated using automated methods were manually adjusted to align only the structurally equivalent regions.

## Neighbouring domains

Conserved domain (CD) search [17] of all TC ZF containing proteins against conserved domain database (CDD) v3.12 (cut-off E-value=0.01) was performed to find the co-occurring domains. This helped to determine plausible evolutionary connections between distantly-related ZFs. Similar searches were simultaneously performed against the Pfam database v27.0 [3] at the Pfam web server with a cut-off E-value=0.01.

## References


1. Krishna, S.S., Majumdar, I. & Grishin, N.V. Structural classification of zinc fingers: survey and summary. *Nucleic acids research* **31**, 532-550 (2003).
2. Murzin, A.G., Brenner, S.E., Hubbard, T. & Chothia, C. SCOP: a structural classification of proteins database for the investigation of sequences and structures. *Journal of molecular biology* **247**, 536-540 (1995).
3. Finn, R.D. et al. Pfam: the protein families database. *Nucleic acids research* **42**, D222-230 (2014).
4. Li, W. & Godzik, A. Cd-hit: a fast program for clustering and comparing large sets of protein or nucleotide sequences. *Bioinformatics* **22**, 1658-1659 (2006).
5. Holm, L. & Rosenstrom, P. Dali server: conservation mapping in 3D. *Nucleic acids research* **38**, W545-549 (2010).
6. Zhang, Y. & Skolnick, J. TM-align: a protein structure alignment algorithm based on the TM-score. *Nucleic acids research* **33**, 2302-2309 (2005).
7. Pandit, S.B. & Skolnick, J. Fr-TM-align: a new protein structural alignment method based on fragment alignments and the TM-score. *BMC bioinformatics* **9**, 531 (2008).
8. Wiederstein, M., Gruber, M., Frank, K., Melo, F. & Sippl, M.J. Structure-based characterization of multiprotein complexes. *Structure* **22**, 1063-1070 (2014).
9. Altschul, S.F., Gish, W., Miller, W., Myers, E.W. & Lipman, D.J. Basic local alignment search tool. *Journal of molecular biology* **215**, 403-410 (1990).
10. Altschul, S.F. et al. Gapped BLAST and PSI-BLAST: a new generation of protein database search programs. *Nucleic acids research* **25**, 3389-3402 (1997).
11. Jaroszewski, L., Rychlewski, L., Li, Z., Li, W. & Godzik, A. FFAS03: a server for profile--profile sequence alignments. *Nucleic acids research* **33**, W284-288 (2005).
12. Finn, R.D., Clements, J. & Eddy, S.R. HMMER web server: interactive sequence similarity searching. *Nucleic acids research* **39**, 18 (2011).
13. Soding, J., Biegert, A. & Lupas, A.N. The HHpred interactive server for protein homology detection and structure prediction. *Nucleic acids research* **33**, W244-248 (2005).
14. Larkin, M.A. et al. Clustal W and Clustal X version 2.0. *Bioinformatics* **23**, 2947-2948 (2007).
15. Hall, T.A. in Nucleic acids symposium series, Vol. 41 95-981999).
16. Pei, J., Kim, B.H. & Grishin, N.V. PROMALS3D: a tool for multiple protein sequence and structure alignments. *Nucleic acids research* **36**, 2295-2300 (2008).
17. Marchler-Bauer, A. & Bryant, S.H. CD-Search: protein domain annotations on the fly. *Nucleic acids research* **32**, W327-W331 (2004).
